# Exploration of immune phenotypes in self-sampling citizens

**DOI:** 10.1016/j.isci.2025.114611

**Published:** 2026-01-03

**Authors:** Leo Dahl, Annika Bendes, María Bueno Álvez, Vincent Albrecht, Hooman Aghelpasand, Sophia Björkander, Simon Kebede Merid, Anja Mezger, Max Käller, Claudia Fredolini, Åsa Torinsson Naluai, Olof Beck, Erik Melén, Stefan Bauer, Magnus Gisslén, Niclas Roxhed, Jochen M. Schwenk

**Affiliations:** 1 SciLifeLab, Department of Protein Science, KTH Royal Institute of Technology, Solna, Sweden; 2 SciLifeLab, Department of Gene Technology, KTH Royal Institute of Technology, Solna, Sweden; 3 Department of Clinical Science and Education, Södersjukhuset, Karolinska Institutet, Stockholm, Sweden; 4 Affinity Proteomics Unit, SciLifeLab Infrastructure, KTH Royal Institute of Technology, Solna, Sweden; 5 Institute of Biomedicine, Sahlgrenska Academy at the University of Gothenburg, Gothenburg, Sweden; 6 Department of Clinical Neuroscience, Karolinska Institutet, Stockholm, Sweden; 7 Sachs’ Children and Youth Hospital, Södersjukhuset, Stockholm, Sweden; 8 TUM Technical University of Munich, Munich, Germany; 9 Department of Infectious Diseases, The Sahlgrenska Academy at University of Gothenburg, Gothenburg, Sweden; 10 Sahlgrenska University Hospital, Gothenburg, Sweden; 11 Public Health Agency of Sweden, Solna, Sweden; 12 Department of Micro and Nanosystems, KTH Royal Institute of Technology Stockholm, Stockholm, Sweden; 13 MedTechLabs, BioClinicum, Karolinska University Hospital, Solna, Sweden

**Keywords:** Health sciences

## Abstract

Blood proteins have provided essential insights into how humans responded to the recent pandemic. To expand our understanding beyond patients seeking medical care, we conducted a citizen-centric survey with 2,000 random residents (age: 18–69 years) from Sweden’s two largest cities in 2021. With self-sampled dried blood spots (DBS) and health information from 437 (22%) volunteers, we performed multi-analyte COVID-19 serology, measured autoantibodies (AAbs) against 22 interferons, and quantified 502 circulating low-abundant immune-related blood proteins. Antibody assays confirmed self-reported infections (26%) and vaccinations (40%), showed timing-dependent discrepancies in the immune response, and revealed anti-type I interferon AAbs co-occurring frequently alongside natural infections. Proteomics data added plausible mechanistic insights into cell-mediated processes: data-driven analyses revealed 24% of participants presented deviating immune phenotypes linked to infections, immunity, respiratory effects, and age. Multi-molecular DBS analysis of random layperson samples captured the broader spectrum of immune system states, adding relevant insights for clinical and public health investigations.

## Highlights

• Survey of blood microsamples with random donors from the general population
• Hundreds of immune system-related proteins were quantified in dried blood microsamples
• Proteins associated with sex, age, infection, and vaccination status
• Data-driven analysis of home-collected microsamples revealed heterogeneous immune phenotypes

## Graphical abstract

**Figure:**
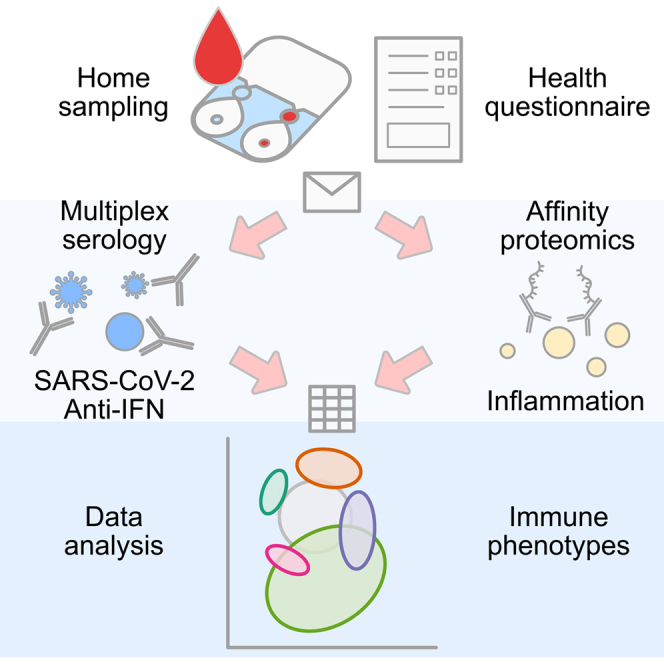

## Introduction

The human immune system has, not least since the recent COVID-19 pandemic, been a focus for monitoring the well-being of those in clinical care due to acute and severe illness or post-acute sequelae. With global infection estimates exceeding 750 million people^1^ (https://covid19.who.int), in Sweden alone, >97% of the adult population and 95% of children <14 years were seropositive in October 2024.^2^ Already in 2021, estimates showed that >30% of the Swedish population were antibody (Ab) positive,^3^ but still, only an overall small fraction of citizens required medical care.

By now, multi-analyte serology approaches are well established to study responses to infection and vaccination,^4^ the repertoire of anti-cytokine autoantibodies (AAbs) has been linked to infections,^5^ and AAbs targeting a wide range of proteins^6^ have been linked to COVID-19, with some emerging alongside infections.^7^ Other AAbs, for example, those against type I interferons (IFNs-I), have been linked to disease severity,^8^ particularly in males,^9^ and in response to infections rather than vaccine.^10^ To learn more about human immune biology, clinical proteomics workflows offered analytical precision, robustness, and depth for revealing wide-ranging health consequences of a person’s current health status,^11^ response to SARS-CoV-2 infections,^12^^,^^13^ cytokine storms,^14^ and immune cell receptor activation.^15^ Still, these approaches were conducted within a hospital or health care setting using venous blood draws.

To close the gap between our clinical and public health understanding of the molecular status and consequences for the population’s immune system, home-sampling has become one valuable approach. With newer devices enabling the self-collection of exact blood volumes from the fingertip,^16^ the generated dried blood spots (DBSs) can be shipped from the user to a laboratory by regular mail. This concept has been proven successful for precise COVID-19 Ab testing^17^^,^^18^^,^^19^; however, studying circulating AAbs or even low abundance inflammatory proteins, such as cytokines or chemokines, has not been possible or evaluated.

Here, we study proteins secreted by the immune systems from over 400 random citizens in self-sampled DBS to elucidate the molecular consequences of SARS-CoV-2 infection and vaccination. Expanding previously established multiplexed assay workflows^18^^,^^20^ into a new sample cohort, studying low abundant inflammatory proteins, and AAbs allows us to explore the molecular heterogeneity among population immune phenotypes.

## Results

### Population of self-sampling citizens

**Figure 1:**
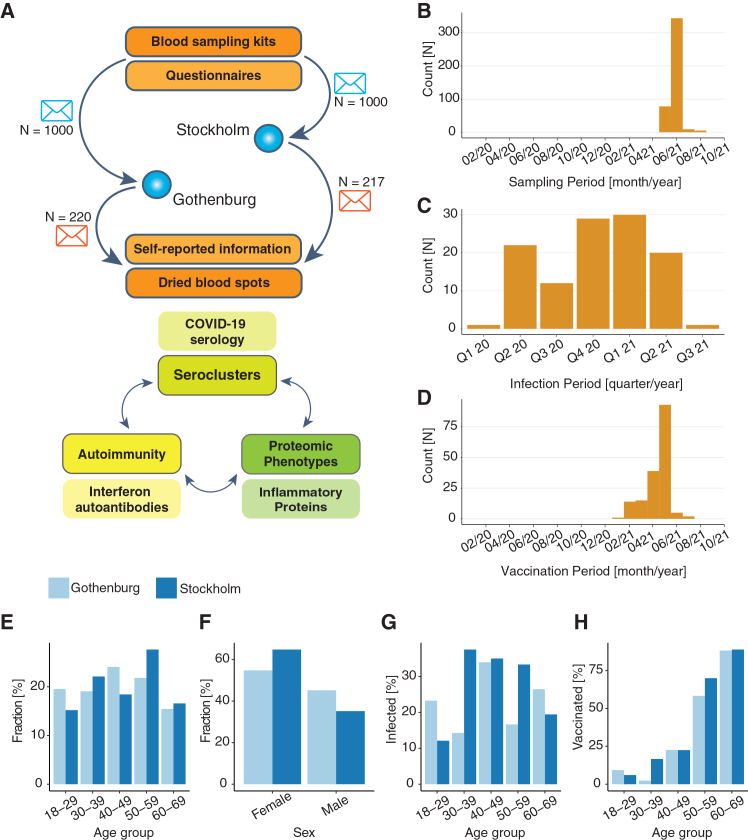
Study design and cohort demographics (A) DBS home sampling kits were sent to 2,000 randomly selected households in Stockholm and Gothenburg. Of the returned kits, 437 were used for downstream analysis. (B) Samples were taken between May and August 2021, peaking around June. (C and D) Self-reported data showed that infection by SARS-CoV-2 was spread out between the beginning of 2020 and Q3 of 2022, (D) while vaccination was concentrated around January 2021 to July 2021. (E) The age of the participants ranged between 18 and 69 years, with fewer participants being in the youngest and oldest age groups (light blue, Gothenburg; dark blue, Stockholm). (F) In both regions, women were more likely to return samples than men. (G and H) Young participants were less likely to have been infected and vaccinated, with vaccination being more common with increasing age.

Two thousand home sampling kits and questionnaires (Data S1) were sent out in April 2021 to randomly selected inhabitants of Gothenburg and Stockholm (1,000 per city) aged 18–69 years of both sexes (Figures 1A and 1B). Our laboratory received 478 (23.9%) DBS cards by postal mail (50% per city), of which, 437 (91.4%) donor samples qualified for further analyses (Table 1) due to complete sampling, questionnaire data, and signed consent. More women than men participated (Figure 1F), and our study contains more donors, aged 40–59 years (Figure 1E) compared to population statistics. In our cohort, 38.4% reported being vaccinated at least once, 26.1% reported a COVID-19 infection before self-sampling (Figures 1C–1G), and 9.4% reported both. Vaccination rate followed the Swedish rollout strategy (Figures 1D–1H), with individuals aged 50–59 and 60–69 years having a higher rate of vaccination (16.0% and 14.2%, respectively) than individuals in the age groups 18–29, 30–39, and 40–49 years (1.37%, 2.06%, and 4.81%, respectively). Overall, these data align well with population statistics in Sweden, including that >30% had been exposed to the virus earlier in 2021,^3^ and nearly 40% had received a vaccine via the national program by early June.^21^

**Table 1:** Self-reported demographics and symptoms

	Gothenburg	Stockholm	Overall
Participants	220 (50.3%)	217 (49.7%)	437 (100%)

**Age group**			

18–29	43 (19.5%)	33 (15.2%)	76 (17.4%)
30–39	42 (19.1%)	48 (22.1%)	90 (20.6%)
40–49	53 (24.1%)	40 (18.4%)	93 (21.3%)
50–59	48 (21.8%)	60 (27.6%)	108 (24.7%)
60–69	34 (15.5%)	36 (16.6%)	70 (16.0%)

**Sex**			

Female	120 (54.5%)	140 (64.5%)	260 (59.5%)
Male	99 (45.0%)	76 (35.0%)	175 (40.0%)
Missing	1 (0.5%)	1 (0.5%)	2 (0.5%)

**Symptoms (loss of)**			

None	178 (80.9%)	170 (78.3%)	348 (79.6%)
Smell	9 (4.1%)	9 (4.1%)	18 (4.1%)
Taste	5 (2.3%)	3 (1.4%)	8 (1.8%)
Smell + taste	28 (12.7%)	35 (16.1%)	63 (14.4%)

**Vaccination**			

Not vaccinated	145 (65.9%)	123 (56.7%)	268 (61.3%)
1 dose	57 (25.9%)	76 (35.0%)	133 (30.4%)
2 doses	18 (8.2%)	17 (7.8%)	35 (8.0%)
Missing	0 (0%)	1 (0.5%)	1 (0.2%)

**Infection**			

No	169 (76.8%)	154 (71.0%)	323 (73.9%)
Yes	51 (23.2%)	63 (29.0%)	114 (26.1%)

### Multi-analyte serology with self-sampled DBS

**Figure 2:**
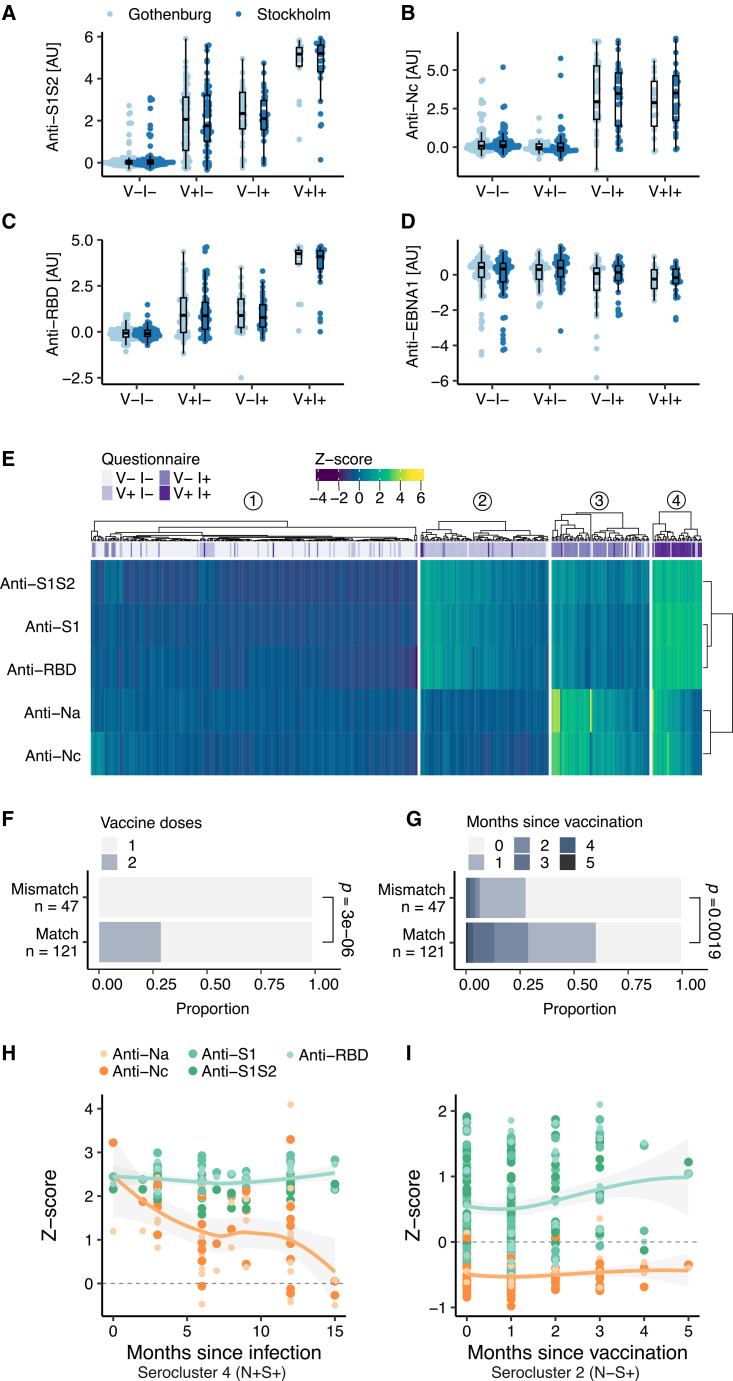
Serological characterization (A–C) Levels of antibodies (Abs) targeting SARS-CoV-2 spike protein were highest in individuals who were both infected and vaccinated (V+ I+). The lowest levels were observed in non-infected, non-vaccinated participants (V- I-), while only infected (V- I+) or only vaccinated (V+ I-) individuals had intermediate levels. For the N protein, V+ I- and V- I+ participants exhibited high levels of Abs, while V+ I- and V- I- stayed low. Light blue, Gothenburg; dark blue, Stockholm. The boxes show the medians (middle line) and first and third quartiles (bottom and top hinges) of each group. Whiskers extend to hinges ±1.5 × interquartile range of the group, at most. (D) Most participants had similar levels of anti-EBNA1 Abs, with lower levels observed in some individuals spread across the immunological states, representing a minority of participants who have not encountered the Epstein-Barr virus. (E) Seroclusters resulting from hierarchical clustering of the anti-SARS-CoV-2 Abs. The clusters match self-reported immunological states well, with some mismatches where Ab levels deviate. The top annotation shows questionnaire response (from light purple to dark purple: V- I-, V+ I-, V- I+, V+ I+). In the heatmap, lighter color means higher signal. (F) Individuals who self-reported only one dose of a vaccine were more likely to be reclassified to seroclusters representing non-vaccinated participants. The *p* value is from Fisher’s exact test. Light, one dose; dark, two doses. (G) The time since vaccination also influences whether individuals with self-reported vaccination status were reclassified to seroclusters representing their vaccination status. The *p* value is from Fisher’s exact test. Lightest, 0 months; darkest, 5 months. (H) The number of months between infection and sampling (taking the average of the 3-month interval in the questionnaire, Data S1, as the time of infection) showed waning levels of anti-N Abs in serocluster 4. (I) Serocluster 2 had stable Ab levels post-vaccination, where those for anti-S Ab levels were higher than for anti-N Ab. Smooth estimates from LOESS regression are displayed for anti-N Abs and anti-S Abs, orange representing anti-N Abs and green anti-S Abs. Shaded areas represent 95% confidence bands.

Based on previous work,^18^^,^^20^ we performed multi-analyte serology of circulating human immunoglobulin G (IgG) antibodies in DBS samples using multiple proteins for spike (S) and nucleocapsid (N) alongside the receptor binding domain (RBD) of S and a data-driven cutoff to classify samples as seropositive. As shown in Table 2, up to 50% of the samples were deemed seropositive for anti-S IgG (denoted S+) and around 20% of the samples contained IgG against N (denoted N+). As shown in Figures 2A–2C for both collection regions, elevated levels of anti-S and anti-RBD were found in samples of donors who reported a natural infection and vaccination, while anti-N levels were elevated in infected participants. We also observed that 23% of infected or vaccinated participants had lower anti-S and anti-RBD levels than expected. Anti-N Abs was detected in 62% of donors who reported being infected and 2.2% of donors reporting no infection. Figure 2D shows the relative IgG response against the Epstein-Barr virus (EBV) antigen EBNA1, which served as a control due to the ∼90% prevalence of anti-EBV Abs.^22^ The DBS serology survey confirms existing knowledge about seroconversion and the possibility to differentiate vaccination and natural infection.

**Table 2:** Multi-analyte SARS-CoV-2 serology

Acronym	SARS-CoV-2 protein	Source	% positive	95% CI
**S1**	spike, S1 domain	KTH	45.3	40.6–50.0
**S1S2**	spike, foldon	KTH	50.3	45.6–55.0
**RBD**	spike, receptor binding domain	KTH	31.1	26.8–35.4
**Na**	nucleocapsid	Acro	20.6	16.8–24.4
**Nc**	nucleocapsid, C-terminal	KTH	19.0	15.3–22.7

SARS-CoV-2 antigens, their sources, and the percent of the samples determined to be seropositive, alongside 95% confidence intervals (CIs). See also Table S1 for anti-IFN AAb frequencies.

### Seroclustering for finer-grained classification of response phenotypes

To obtain even finer-grained insights into the actual Ab response at sampling, beyond self-reports, applying hierarchical clustering revealed four main groups, denoted as seroclusters (Figure 2E). Each serocluster mainly contained one of the four expected combinations of infection (I) and vaccination (V), allowing us to study discrepancies between reported events and measured immune response. As summarized in Table 3, serocluster 1 was the largest cluster (54% of all), with just 2% seropositive samples. Despite our serological results, 39 (16.5%) donors reported vaccination, and 18 (7.6%) reported a previous SARS-CoV-2 infection. In serocluster 2, >90% of the donors self-reported to be vaccinated. Serology data supported this by revealing 95% S+ and no N+ samples. Serocluster 3 contained mostly those who reported an infection. This was supported by 97% being S+ and nearly >70% being N-. Serocluster 4 was the smallest cluster (8% of all), mainly containing participants who reported an infection and vaccination.

**Table 3:** Seroclusters and self-reported demographics

Seroclusters	1	2	3	4	*p* value
Participants	237 (54.2%)	93 (21.3%)	71 (16.3%)	36 (8.2%)	–

**Serology**					

N+ (% cluster)	0 (0%)	0 (0%)	52 (73.2%)	26 (72.2%)	<0.001
S+ (% cluster)	5 (2.1%)	88 (94.6%)	69 (97.2%)	36 (100%)	<0.001

**Age Group**					

18–29	54 (22.8%)	6 (6.5%)	14 (19.7%)	2 (5.6%)	<0.001
30–39	66 (27.8%)	8 (8.6%)	13 (18.3%)	3 (8.3%)	
40–49	56 (23.6%)	9 (9.7%)	20 (28.2%)	8 (22.2%)	
50–59	48 (20.3%)	32 (34.4%)	19 (26.8%)	9 (25.0%)	
60–69	13 (5.5%)	38 (40.9%)	5 (7.0%)	14 (38.9%)	

**Sex**					

Female	131 (55.3%)	68 (73.1%)	40 (56.3%)	21 (58.3%)	0.013
Male	106 (44.7%)	24 (25.8%)	30 (42.3%)	15 (41.7%)	
Missing	0 (0%)	1 (1.1%)	1 (1.4%)	0 (0%)	–

**Region**					

Gothenburg	131 (55.3%)	41 (44.1%)	35 (49.3%)	13 (36.1%)	0.084
Stockholm	106 (44.7%)	52 (55.9%)	36 (50.7%)	23 (63.9%)	

**Symptoms (loss of)**					

None	218 (92.0%)	82 (88.2%)	32 (45.1%)	16 (44.4%)	<0.001
Smell	5 (2.1%)	2 (2.2%)	7 (9.9%)	4 (11.1%)	
Taste	1 (0.4%)	2 (2.2%)	4 (5.6%)	1 (2.8%)	
Smell + Taste	13 (5.5%)	7 (7.5%)	28 (39.4%)	15 (41.7%)	

**Vaccination**					

Not vaccinated	198 (83.5%)	7 (7.5%)	62 (87.3%)	1 (2.8%)	<0.001
1 dose	39 (16.5%)	64 (68.8%)	8 (11.3%)	22 (61.1%)	
2 doses	0 (0%)	22 (23.7%)	0 (0%)	13 (36.1%)	
Missing	0 (0%)	0 (0%)	1 (1.4%)	0 (0%)	

**Infection**					

No	219 (92.4%)	85 (91.4%)	15 (21.1%)	4 (11.1%)	<0.001
Yes	18 (7.6%)	8 (8.6%)	56 (78.9%)	32 (88.9%)	

**IFN AAb positivity**					

No	191 (80.6%)	75 (80.6%)	48 (67.6%)	31 (86.1%)	0.083
Yes	46 (19.4%)	18 (19.4%)	23 (32.4%)	5 (13.9%)	

*p* values are from Fisher’s exact tests.

These response phenotypes generally agreed well with the infection and vaccination statuses in the questionnaires and even allowed us to quantify the improvement achieved by introducing the seroclusters. Predictions of age, sex, and region using Ab levels were expectedly poor, with areas under the curve (AUCs) around 0.6 (hand till average for age), while the AUC was 0.85 for self-reported vaccination and 0.89 for infection. For the seroclusters, the average AUC was 0.99, ranging between 0.95 and 1.0 (Figure S1, Table S2). Hence, stratification by seroclusters allowed for better classification of the individuals.

Differences between the molecular and self-reported data were predominantly linked to subjects self-reporting vaccination, where about 11% of the samples (*N* = 47) showed discrepancies in reported vaccination and immune response. These differences were, as shown in Figures 2F and 2G, due to the number of doses and the time between sampling and vaccine administration. This confirms the utility of seroclustering to capture even subtle differences in seroconversion.

Lastly, we investigated the effect of time between COVID-19-related events and sampling. As shown in Figure 2H for participants from serocluster 4, the levels of anti-S antibodies remained stable over 15 months while levels of anti-N decreased. In comparison, anti-S levels remained elevated and anti-N levels remained low in vaccinated subjects of serocluster 2 (Figure 2I).

In summary, data-driven seroclusters provided a finer-grained description of the molecular immune status at sampling, compensating for effects of time elapsed since exposure.

### AAbs against IFNs co-occur alongside infections

COVID-19 infection has been linked to AAbs against IFNs^23^; hence, their occurrence in the population could reveal relevant relationships between infections and health status. Utilizing the multiplexing capacity of the assays system, we tested the DBS samples for AAbs against 22 full-length IFN proteins. As shown in Table S1, and at 12× standard deviation (SD) above population peak for each IFN, anti-IFN AAbs were detected against all IFNs but IFN-beta with frequencies from 0.2% to 9% (mean of 2.1%; 95% confidence interval [CI]: 1.2–3.0). AAbs against IFN-alpha 17 (IFNA17) were most prevalent (8.9%), followed by IFN-gamma (IFNG, 5.7%) and IFN-omega (IFNW) 1 (4.8%). Across all samples, 92 donors were AAb+ for at least one of the 22 IFN (21%), and while the highest percentage of AAb+ samples (6.2%) were found in serocluster 3 (>80% self-reported infections). This suggests that recent natural infections rather than vaccinations influenced the occurrence of anti-IFN AAbs.

To corroborate this observation, we also analyzed the AAb frequencies by sex, age, symptoms, and seropositivity, and as detailed in Figure S2, frequencies were higher in women (*p* < 0.05) for IFNG. AAb frequencies did not differ between age ranges (Figure S3); however, women below 40 years had a higher, albeit not statistically significant, prevalence of AAbs against IFNW1, IFNA17, and IFNG (10.3%) compared to women above 40 years of age or men (Figure S4). AAb frequencies were linked to N+ across many IFNA subfamily members (Figure S5) and were more prominent than AAb frequencies related to S+ samples (Figure S6), but none of the highly prevalent AAbs (IFNA17, IFNG, and IFNW1) were linked to seropositivity. Anti-IFN Abs against the IFN A family showed patterns of co-occurrence using hierarchical clustering (Figure S7A). As shown in Figure S7B, Fisher’s exact tests revealed a dominant association of AAbs against members of the IFNA subfamily in N+ samples.

This analysis found elevated reactivity levels of AAbs against the IFNA subfamily in primarily infected (N+) participants and in women under 40.

### Profiling the circulating proteome reveals distinct health and immune phenotypes

The serological and AAb analyses classified donors based on their Ab-centric immune response to natural infections and vaccinations. To gain further insights into how a wider range of processes of the immune systems are influenced by the exposures, we studied >500 mostly low abundant inflammation-related proteins in the DBS samples of our cross-sectional cohort.

### Assessment and validation of proteomics data derived from complementary assays

First, we used two complementary affinity proteomics methods branded to cover 368 and 250 circulating proteins described to be involved in inflammation. Out of all 618 protein assays, there were 502 unique protein targets of which, 110 overlapped between the platforms (Figure S8G). This offered us an immediate possibility to validate the data, and as shown in Figure S8H, comparative analysis revealed 60 protein profiles (54.5%) to correlate between the two methods (rho >0.5) and showed a clear trend for proteins above the limit of detection (LOD; see Table S3). Both platforms delivered highly precise data, with 508 protein assays (82.2%) detected above LOD in >50% of the samples and coefficients of variation (CVs) <10% for 51% (median 9.9%) of the targets (Figure S8E). These 508 protein assays, targeting 435 unique proteins, were used for global analysis to identify outliers. This revealed two outlier samples, one of which was detected in both platforms (Figures S8A and S8B), while principal-component analysis (PCA) did not reveal any imminent clusters that could have biased the downstream analyses (Figure S8C).

Checking the variance of protein levels across all samples (Figure S8F), we found the widely expressed adaptor protein CRKL was the most stable protein, while the levels of the monocyte protease CTSS, or cathepsin S, varied the most across the DBS samples, likely due to its bimodal distribution. The second most variable protein was the cytokine interleukin-36-gamma (IL-36G), which, according to the Human Protein Atlas,^24^ is expressed in respiratory epithelial cells, skin, and tonsils.

This evaluation confirms previous investigations with plasma samples^20^ and the utility of both methods to detect low abundant immune system proteins in DBS analysis. Large cross-platform comparisons are of growing interest but require other omics data for a detailed assessment;^25^ hence, we limited our downstream analysis to proteins with >50% sample detection rates above LOD.

### Associations of circulating proteins with Abs against SARS-CoV-2 and IFN antigens

Next, we determined the relationships between circulating protein levels and individual SARS-CoV-2 and IFN antigens (Table S4), limiting this analysis to protein assays showing concordant nominal cross-platform associations.

For SARS-CoV-2, several immune-related proteins were associated with S and RBD and, as shown in Figure S9, includes positive associations with triggering receptor expressed on myeloid cells 2 (TREM2), a protein regulating the balance between anti-inflammatory and pro-inflammatory responses.^26^ Similar trends were observed for the inflammation-promoting chemokine (CXCL8) and the immune-cell activating chemokine (CXCL9) (chemokine ligand 9, also known as monokine induced by gamma interferon (MIG). A negative association trend was found between S/RBD and the acute phase, neutrophil-attracting chemokine ligand 1 (CXCL1), suggesting that most samples were likely obtained after the acute phase of the infection. This aligns with the negative association trend with the inhibitory receptor CD200R1 (cell surface transmembrane glycoprotein CD200 receptor 1), which has reduced levels despite higher levels of anti-SARS-CoV-2 Abs.

For IFN AAbs, the association analysis with proteomics revealed eight proteins, most related to cytokine signaling. As shown in Figure S9, this included associations with more than one AAb against IFN alpha family members. For example, the protease inhibitor cystatin-F (CST7), the T cell development cytokine IL-7, and the regulatory growth factor transforming growth factor β 1 had a positive association trend with several type I IFNs. IFNA17, the target with the highest AAb frequency, was only associated with increasing levels of histocompatibility leukocyte antigen (HLA)-DRA, the alpha subunit of HLA class II histocompatibility antigen DR, a major histocompatibility complex (MHC) class II receptor found on antigen-presenting cells. The T cell activating receptor tumor necrosis factor receptor superfamily, member 4 was negatively associated with anti-IFN AAbs. For other tested IFNs, only negative associations of anti-IFNLR1, AAbs, and IRAK4 (IL-1 receptor-associated kinase 4), a protease activating the innate immune response, were observed and platform consistent.

The analysis of circulating immune system proteins in connection with antibodies against foreign and self-antigens revealed additional processes linked to cellular immune regulation and activation.

### Circulating proteins associate with immune phenotypes from seroclusters

Making use of the previously described seroclusters, which reflect some of the immune response at the time of sampling, we investigated possible links between this phenotype classification and circulating proteins. There were 18 (3.5% of 508) proteins significantly associated false discovery rate (FDR <0.05) with seroclusters (Figure S10, Tables S5 and S6), including the above-mentioned CXCL9 and TREM2. The endothelial leucine aminopeptidase 3 (LAP3), involved in protein degradation, had increased levels in seroclusters related to infection and/or vaccination. The protein Hepatitis A virus cellular receptor 1 (HAVCR1), also called KIM-1, was elevated in vaccinated seroclusters, aligning with its involvement in various biological processes, such as a host cell virus receptor, immune regulation, and kidney injury. Inversely, the extracellular protein matrix extracellular phosphor-glycoprotein (MEPE) and the endocrine neuropeptide galanin (GAL) decreased similarly in these seroclusters. Other noteworthy observations concerned differences between serocluster 2 (vaccinated but uninfected) and other seroclusters, reflecting different molecular processes. For example, lower levels were found for agouti-related protein (AGRP), an endocrine hormone in food and energy control; cytokine receptor-like factor 1 (CRLF1), a cytokine secreted by smooth muscles, different fibroblasts, and brain cells for cell growth and differentiation; and secretogranin III (SCG3), a protein secreted by brain cells and endocrine tissue to form secretory granules. Higher levels in serocluster 2 were found for CXCL9, the gastrointestinal regulator protein motilin (MLN), and chemokine ligand 17 (CXCL17), a chemokine secreted by salivary and respiratory epithelium and glands in the stomach. These associations confirm that plausible immune system-related changes of low abundant proteins can be determined in self-sampled DBS.

### Utility of circulating proteins for predicting donor- and health-related traits

Knowing that person-specific factors may influence the levels of the circulating proteome and confound disease-specific investigations,^27^ we tested age and sex associations (Figure S11, Table S7) in the seronegative population. This pointed to plausible links between seropositivity and individual immune response. However, due to the vaccine rollout strategy in Sweden, there is an intrinsic link between age and vaccination. LAP3, CRLF1, and PON3 showed associations with seroclusters but not with age.

**Figure 3:**
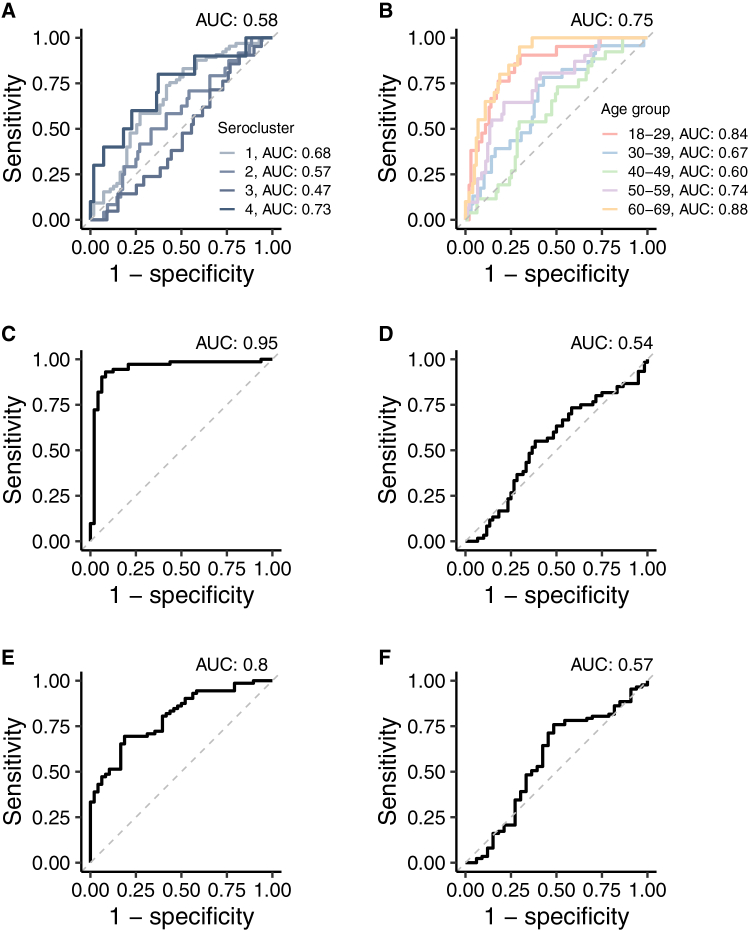
Predicting traits using circulating proteins (A–F) Receiver operating characteristic (ROC) curves for prediction of (A) serocluster (the darker the color the higher the serocluster number), (B) age (pink, 18–29; blue, 30–39; green: 40–49; purple: 50–59; orange: 60–69 years old), (C) sex, (D) region, (E) vaccination, and (F) infection using circulating proteins as predictors in penalized multinomial regression. For serocluster and age prediction with more than two classes, the area under the curve (AUC) shown at the top of the plot is the average AUC from the Hand Till method. See Figure S8 for proteomics quality controls.

To further test the utility and reliability of the proteomics data, we performed a multivariate Lasso regression analysis to predict donor- and health-related traits from circulating protein data (Figure 3, Table S8). Prediction performed best for vaccinated and infected groups while being poorer for other seroclusters (Figure 3A; AUC 0.47 to 0.73), and as expected, this was outperformed by serology (AUC >0.85). Proteomics data as expected outperformed serology, (AUC <0.7) as predictors of age, with the youngest and oldest age groups showing higher predictive performance than the intermediate age groups (Figure 3B; AUC 0.60 to 0.88). Given the broad influence of sex on the human proteome, sex of the donor could be predicted with high accuracy (Figure 3C; AUC 0.95). In contrast, the geographical location of the self-sampling donor could not be predicted (Figure 3D; AUC 0.54). Proteomics data were a weak predictor for self-reported infection (Figure 3E; AUC 0.57), likely due to the low number of current or recent infections, while self-reported vaccination could be predicted moderately well (Figure 3F; AUC 0.80), as these occurred more recently. However, vaccination was biased toward elderly and prioritized individuals; hence, adjusting the data for age reduced the AUC to 0.5.

These investigations confirm the utility of proteomics data and reveal how well-established traits, such as age and sex, influence protein levels to a higher degree than the serology or autoimmune data.

### Data-driven analysis reveals heterogeneous health and immune phenotypes

Thus far, our analyses categorized the proteomics data based on other available data. We hypothesize that COVID-unrelated processes, such as other diseases or medical history, may influence protein levels and drive our classifications and predictions. Consequently, we applied a proteomics data-driven approach to determine immune states and health phenotypes from the perspective of the circulating proteome.

Initially, we reduced the number of variables originating from the 500+ protein-level dimensions by performing Weighted Gene Correlation Network Analysis (WGCNA). This resulted in five distinct WGCNA modules, denoted by the colors blue, brown, green, turquoise, and yellow. Each module contained between 30 and 72 proteins (from 32 to 83 assays), while a sixth (gray) module contained the remaining uncorrelated 167 proteins (182 assays) (Figures S12A and S12B, Table S9). Of 73 proteins measured with two methods, 54 (74%) had both assays assigned to the same module (Figure S12C).

We then annotated the underlying biology of the WGCNA module proteins by using protein associations with 28 traits from a large-scale proteomics UK Biobank (UKB) study^27^ (Figure S13, Table S10). This revealed that the blue module was enriched in proteins associated with the respiratory system, such as smoking and matrix metallopeptidase 10 (MMP10), alveolar macrophages (agouti-related protein or AGRP), lung-specific proteins such as SCGB1A1 (secretoglobin family 1A member 1, also called CC16), and kidney and liver function. The brown module was rich in proteins associated with age and many other health-related aspects tested in the UKB study. Inversely, the green and yellow modules contained fewer proteins associated with sex and health phenotypes, including smoking and lower respiratory infection. The turquoise module was enriched in proteins associated with acute lower respiratory infection. To investigate deeper links with immune function, we determined the immune cell enrichment of proteins in each module (Figure S14). The turquoise module was enriched in cells such as neutrophils, eosinophils, and dendritic cells while not in T cells, suggesting that the module is connected to the innate immune system. The STRING database (v.12.0) was used to add a functional note to each module (Figure S15). The blue module was found to be linked to T cell function, the brown, turquoise, and gray modules to cell migration, chemotaxis, and cytokine response, and the yellow module to intracellular signaling through pathways such as the mitogen-activated protein kinase (MAPK) and phosphoinositide 3-kinase pathways. The green module was linked to cell signaling (MAPK) and immune response (nuclear factor κB), as well as recognition of pathogen DNA (via Toll-like receptor 9), suggesting a role in recent infection by pathogens other than SARS-CoV-2.

Using these five distinct and physiologically plausible WGCNA modules, we computed the module eigengenes (MEs), which are the first principal components of the constituent proteins. These five MEs were then used for clustering, revealing 14 protein-based phenotypes, denoted proteotypes. Stability analysis using the mean Jaccard index (MJI) shortlisted five stable clusters that met the criteria of containing *N* > 10 individuals and an MJI > 0.5. These five proteotypes represented 24% (*N* = 95) of all donors and were labeled (1–5) in order of decreasing size (*N* = 30, 20, 18, 16, and 11). The less stable proteotypes were aggregated into proteotype 0 with 76% of all participants (*N* = 300).

**Figure 4:**
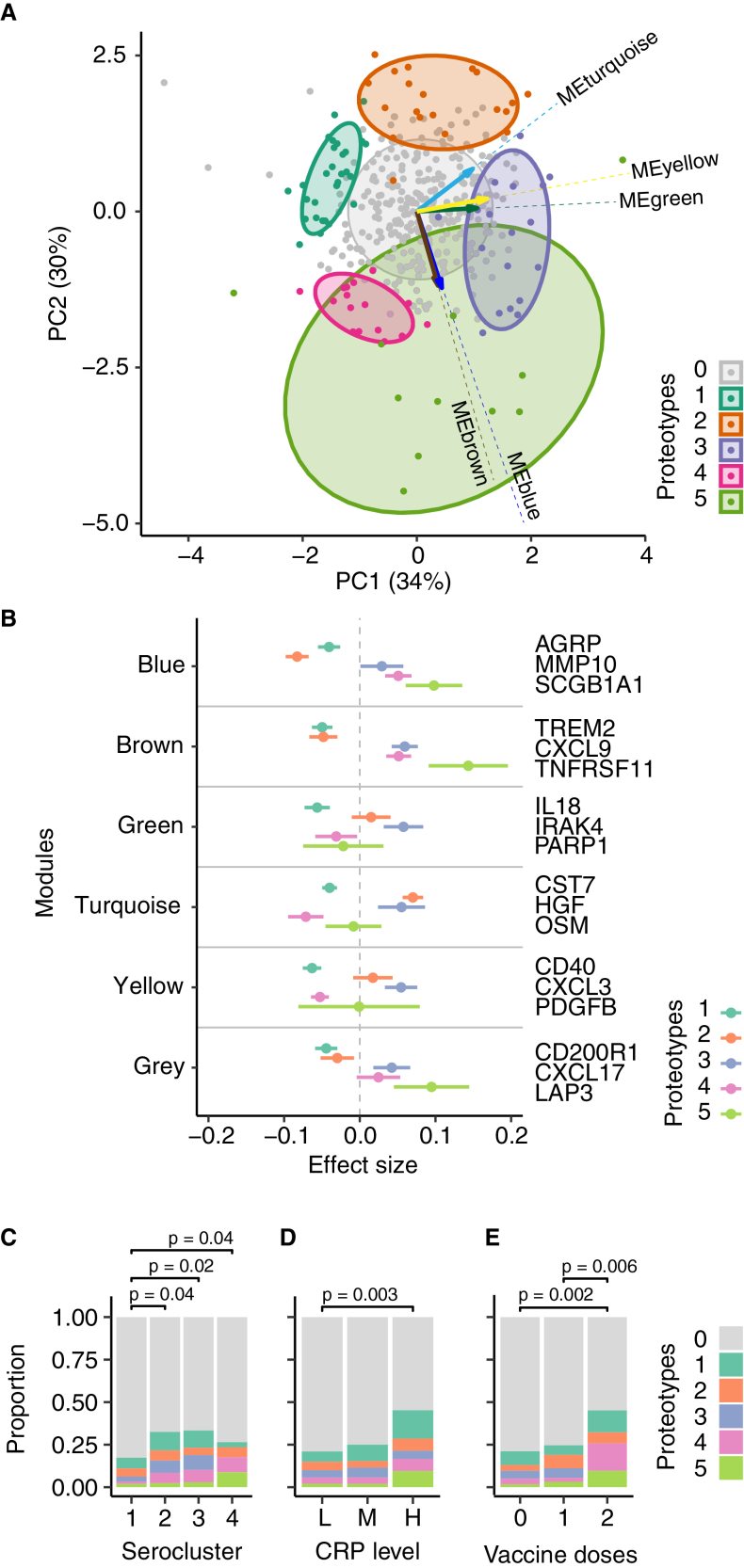
Clustering of samples based on WGCNA protein modules (A) Principal-component analysis (PCA) biplot of the Weighted Gene Correlation Network Analysis (WGCNA) module eigengenes (MEs), colored by proteotype membership (dark green, 1; orange, 2; blue, 3; pink, 4; light green, 5; gray, 0). The ellipses represent 68% of each group assuming normal distributions. (B) Comparison of module effect sizes of the proteotype. The effect sizes were measured as the difference in ME mean between each proteotype 1–5 and the remaining proteotypes. The horizontal bars show the 95% confidence intervals. Three examples of proteins from each module are shown to the right. (C) Distributions of proteotypes within each serocluster (*N* = 208, 83, 69, and 34 for seroclusters 1, 2, 3, and 4, respectively). (D) Distributions of proteotypes across low (L, *N* = 300), medium (M, *N* = 52), and high (H, *N* = 42) C-reactive protein (CRP) levels. (E) Distributions of proteotypes across the number of vaccine doses (*N* = 237, 126, and 31 for 0, 1, and 2 doses, respectively). *p* values are from Fisher’s exact test. See Table S9 for module memberships of proteins, and Figures S12–S16 for module protein numbers, protein-trait associations, immune cell enrichment, pathway enrichment, and protein principal-component analysis.

Using a PCA biplot showed that the five stable proteotypes clustered around the perimeter of the unstable proteotype in PC1-PC2 space (Figure 4A), but less so for PC3 and PC4 (Figure S16). To exemplify how representative proteins contributed to this classification, Figure 4B compares the distinct WGCNA module profiles of proteotypes 1–5 using standardized effect sizes. While effect sizes for proteotype 1 were negative across all modules, proteotype 3 was characterized by positive effect sizes across all modules. Proteotype 2 showed positive effect sizes for the turquoise module, while those for the blue and brown modules remained negative. Effect sizes of proteotype 4 were positive for the blue and brown modules and negative for all others. Proteotype 5 showed the largest positive effect sizes for the blue and brown modules and the gray module. This analysis also showed that some proteotypes overlapped in the trends and magnitudes of effect sizes, for example, proteotypes 3 and 4 were similar for the blue and brown modules but different for the green, turquoise, and yellow ones.

To classify the proteotypes, we first studied their differences in questionnaire information (Table 4), and in contrast to serology, found no significant differences in age, sex, or self-reported symptoms. The influence of infection, vaccination, and seroclustering, however, remained observable but more evenly distributed across these proteomic phenotypes. Still, seronegative individuals from serocluster 1 had a lower proteotype heterogeneity than seropositive clusters (Figure 4C), pointing to the effect of infections and vaccinations on the levels of immune-related proteins. There was no significant co-occurrence of anti-IFN AAbs with the proteotypes, nor an association with the levels of the well-established but unspecific inflammation marker C-reactive protein (CRP). Categorizing the proteotypes by tiers of high, medium, and low CRP levels revealed high proportions of individuals with elevated CRP levels in proteotypes 1 and 5, indicating ongoing infections or inflammation-related processes (Figure 4D). In contrast, proteotype 4 had high proportions of infected and vaccinated donors but low CRP levels, suggesting a waning proteomics immune response, confirming the serological data (Figure 4E).

**Table 4:** Self-reported and serological demographics of proteomics phenotype

Proteotypes	0	1	2	3	4	5	*p* value
Participants	300 (75.9%)	30 (7.6%)	20 (5.1%)	18 (4.6%)	16 (4.1%)	11 (2.8%)	–

**Age group**							

18–29	52 (17.3%)	8 (26.7%)	3 (15.0%)	5 (27.8%)	2 (12.5%)	0 (0%)	0.24
30–39	60 (20.0%)	3 (10.0%)	3 (15.0%)	3 (16.7%)	3 (18.8%)	4 (36.4%)	
40–49	64 (21.3%)	9 (30.0%)	7 (35.0%)	1 (5.6%)	3 (18.8%)	0 (0%)	
50–59	80 (26.7%)	5 (16.7%)	3 (15.0%)	6 (33.3%)	3 (18.8%)	3 (27.3%)	
60–69	44 (14.7%)	5 (16.7%)	4 (20.0%)	3 (16.7%)	5 (31.3%)	4 (36.4%)	

**Sex**							

Female	183 (61.0%)	17 (56.7%)	10 (50.0%)	10 (55.6%)	11 (68.8%)	6 (54.5%)	0.86
Male	117 (39.0%)	13 (43.3%)	10 (50.0%)	8 (44.4%)	5 (31.3%)	5 (45.5%)	

**Infection status**							

0	225 (75.0%)	22 (73.3%)	16 (80.0%)	12 (66.7%)	8 (50.0%)	4 (36.4%)	0.025
1	75 (25.0%)	8 (26.7%)	4 (20.0%)	6 (33.3%)	8 (50.0%)	7 (63.6%)	

**Vaccination status**							

0	187 (62.3%)	19 (63.3%)	8 (40.0%)	11 (61.1%)	8 (50.0%)	4 (36.4%)	0.005
1	95 (31.7%)	7 (23.3%)	10 (50.0%)	7 (38.9%)	3 (18.8%)	4 (36.4%)	
2	17 (5.7%)	4 (13.3%)	2 (10.0%)	0 (0%)	5 (31.3%)	3 (27.3%)	
Missing	1 (0.3%)	0 (0%)	0 (0%)	0 (0%)	0 (0%)	0 (0%)	

**Symptoms (loss of)**							

None	245 (81.7%)	25 (83.3%)	15 (75.0%)	14 (77.8%)	10 (62.5%)	6 (54.5%)	0.14
Smell	8 (2.7%)	1 (3.3%)	2 (10.0%)	2 (11.1%)	1 (6.3%)	1 (9.1%)	
Taste	6 (2.0%)	0 (0%)	0 (0%)	0 (0%)	0 (0%)	1 (9.1%)	
Smell + Taste	41 (13.7%)	4 (13.3%)	3 (15.0%)	2 (11.1%)	5 (31.3%)	3 (27.3%)	

**Serocluster**							

1	172 (57.3%)	13 (43.3%)	10 (50.0%)	6 (33.3%)	3 (18.8%)	4 (36.4%)	0.025
2	56 (18.7%)	9 (30.0%)	5 (25.0%)	6 (33.3%)	5 (31.3%)	2 (18.2%)	
3	47 (15.7%)	7 (23.3%)	3 (15.0%)	6 (33.3%)	5 (31.3%)	2 (18.2%)	
4	25 (8.3%)	1 (3.3%)	2 (10.0%)	0 (0%)	3 (18.8%)	3 (27.3%)	

**C-reactive protein (CRP) level category**							

Low	238 (79.3%)	18 (60.0%)	15 (75.0%)	13 (72.2%)	11 (68.8%)	6 (54.5%)	0.045
Medium	39 (13.0%)	5 (16.7%)	2 (10.0%)	3 (16.7%)	2 (12.5%)	1 (9.1%)	
High	23 (7.7%)	7 (23.3%)	3 (15.0%)	2 (11.1%)	3 (18.8%)	4 (36.4%)	

**IFN AAb positivity**							

No	237 (79.0%)	23 (76.7%)	19 (95.0%)	13 (72.2%)	12 (75.0%)	9 (81.8%)	0.5
Yes	63 (21.0%)	7 (23.3%)	1 (5.0%)	5 (27.8%)	4 (25.0%)	2 (18.2%)	

*p* values are from Fisher’s exact tests.

The data-driven immune phenotype analysis highlighted the heterogeneity in this population study, attributed to a combination of COVID-related and other pre-existing health conditions.

## Discussion

We performed multi-modal serological, autoimmune, and proteomic analyses on home-sampled dried blood in a general population to elucidate the features of the immune response against natural infection and vaccination. Our study expands the suitability of self-sampling as a strategy for multi-molecular and extracellular analysis, comparable to clinical studies using venous blood draws.^18^^,^^19^^,^^20^ While little was known about the effects of the pandemic exposures on the general population, employing easy-to-perform quantitative sampling kits for random individuals allowed us to gain valuable insights into the citizen’s immune system during the pandemic. A reduced bias compared to self-enrollment studies was achieved, while maintaining a low sampling failure rate in an untrained population. The timing of sampling, scheduled during a time when only a subset of individuals had been exposed to the virus or vaccinated against COVID-19, makes our study set unique.

Our serological study confirmed the expected immune biology and allowed us to infer the infection and vaccination status of individuals by the differences in immune response, as shown previously.^28^ The investigation focused on persisting IgG-type antibodies, lacking insights into acute, shorter-term response mediated by immunoglobulin M (IgM). However, the multi-analyte data-driven seroclustering provided time-resolved molecular insights into the humoral response, allowing us to classify the individuals by their circulating Ab response with high predictive precision (AUC >0.99) and reporting the waning of anti-N antibodies or an incomplete development of anti-S response due to recent vaccinations.

The multiplexed platform allowed us to add assays for autoreactive IgG against human IFNs alongside the SARS-CoV-2 antigens. These anti-IFN AAbs were enriched to co-occur in infected donors. Earlier discussions about anti-SARS-CoV-2 antibodies suggested cross-reactivity may occur *in vivo* and cause disease pathologies^29^; however, it remains controversial whether AAbs to IFNs existed before or only after infection. A recent study supports our observations, suggesting that mucosal antibodies against IFNs can appear temporarily, even in milder cases.^30^ Despite the need for further investigations, it is reasonable to suggest that we could detect anti-IFN AAbs in DBS due to the presence of hematopoietic cells or interstitial fluid (in addition to the liquid plasma fraction). Still, we cannot exclude the possibility that anti-N antibodies cross-react with the immobilized IFN-I in our assay and future studies should attempt to validate this observation. Our data also allowed us to study nine IFN proteins alongside their matching AAbs; however, there was no association between the two data types. Despite the co-occurrence with natural infections, it is worth noting that frequencies of some of the anti-IFN AAbs detected in our general population study were closer to those reported for severe disease.^31^ As shown in Data S2, and taking advantage of the multiplexed assay readout, a possible reason for these higher numbers could be due to the narrower (homogeneous) distribution of the background or a less upward-skewed distribution. Even at a 12 SD cutoff above the population peak, our procedure labeled samples as AAbs+ when the detected reactivity levels are closer to the background than for other antigens. Hence, each antigen was judged individually and independently, and in our case, those with a wider (heterogeneous) background distribution revealed fewer AAb+ samples. Ultimately, alternative and pre-validated assays together with paired DBS and plasma samples from AAb+ donors are needed to confirm such observations. Preferably, we suggest using resampling as a strategy to gain certainty about the consistency of an observation, allowing also studying the same sample type by the same assay. Using multiplexed assays, the comparison of anti-COVID Abs in DBS with plasma has been shown to reveal consistent results.^18^ Hence, our study suggests continuous development of unifying the criteria that determine cutoff levels across assays with different background and performance characteristics. Moreover, our data add some relevant insights to the currently still incomplete understanding of anti-IFN AAbs in COVID-19.

Profiling the circulating proteome has given important insights into acute and severe COVID-19,^32^ and studies continue to emerge, providing more knowledge about post-COVID-19 sequelae. We chose two complementary affinity proteomics platforms^33^^,^^34^ to determine and validate extracellular immune-related proteins in DBS. This expands previous work^20^ into much lower protein abundances and toward immune system proteins revealing more acute and shorter-lived signatures. The observed platform differences match those made in plasma samples,^34^^,^^35^ highlighting assay sensitivity as a key for reliable detection of low abundant proteins. Still, the remaining discrepancies and impressions call for further investigation with orthogonal methods to increase confidence in technology-dependent observations.^11^

Adding proteomics to Ab data provided more fine-grained insights into molecular processes of the immune system. For example, the generally low levels of well-known acute-phase proteins, such as CRP, IL-6, TNF, or IFNG, suggested that most samples were taken after the acute phase of the infection. The stability of the molecular features appeared as a major differentiator of circulating proteins and anti-SARS-CoV-2 Abs. Since such anti-COVID Abs did not exist before infection, they must have been acquired during the pandemic. Certain circulating protein profiles, however, are also known to be stable and person-specific,^36^^,^^37^ hence existed before this peri-pandemic sampling. Despite proteins changing in response to infection or vaccination,^38^ our study acknowledges that each data type can provide a different view on the human immune response: anti-SARS-CoV-2 Abs are limited to COVID-19; anti-IFN AAbs could be acquired or pre-existing and relate to disease vulnerability or autoimmune conditions, while the pleiotropy of circulating proteins can reveal immune-related as well as a wider range of “unrelated” phenotypes.

We also evaluated the reduction of the 500+ proteomics data features into informative dimensions. WGCNA revealed select sets of proteins that allowed us to identify different plausible phenotypes and provide clinically informative health projections and insights into cell-mediated immune processes. The protein modules revealed important COVID-19 pathologies, such as respiratory distress as represented by MMP10,^39^ found in respiratory epithelial cells; the lung-specific SCGB1A1^40^; interactions with viruses through renal receptor HAVCR1^41^; inflammation via CRP or pro-inflammatory chemokines including CXCL9 or immune cell receptors such as TREM2.^42^ Our study also included other pleiotropic inflammation blood biomarkers, such as IL-6, TNF, and IFNG, which are known to be elevated in acute disease when studying plasma samples.^12^^,^^43^ However, these were not shortlisted as informative features in our modules likely due to the lower number of acute or active cases. Despite the unique aspect of focusing on the immune system, and support from clinical studies,^43^^,^^44^^,^^45^ physiologically interconnected processes such as coagulation or organ damage can influence the molecular phenotypes indirectly. As discussed elsewhere,^20^ the use of dried samples for multi-molecular analysis of extracellular biomarkers has just started. Hence, analytical and sample-related factors connected to the drying and leakage of intracellular proteins can influence the assay performance of any analyte, including those postulated by other studies.

This study highlights the importance of understanding population-level immune responses in the context of underlying health conditions. These may, depending on age, sex, socio-economic, and many other factors, affect a large proportion of the population. Taking smoking as an example, 5% of Swedish adults report smoking daily, and 6% report smoking occasionally.^46^ The effect of this could be observed in the blue protein module, containing MMP10 and SCGB1A1, and as such, support the development of scalable tools to monitor and manage public health trajectories and resources effectively. These could also benefit studies of long-COVID since several of the presented markers, such as CRP, CXCL9, anti-IFN AAbs, and CST7, have been shortlisted.^47^

Several statistical methods were used in this study, many assuming independence of observations, which is fulfilled through cross-sectional sampling of random individuals. Some non-parametric tests were used as to not assume normality of the data; however, at > 400 samples, the central limit theorem means that normality would likely not be an issue. Given the inflammation focus of the protein panel in the study, many proteins are likely to co-vary and correlate. While Lasso can deal with multicollinearity, this could present an issue for the other regression analyses. Finally, these linear regression methods assume linear relationships, which may not be the case for all proteins.

Population self-sampling has provided precise insights into the molecular heterogeneity of the immune profiles during SARS-CoV-2 exposures and vaccination. Confirming expected and discovering deviating health phenotypes provides in a new sample type adds valuable data for risk stratification, remote patient management and outreach to ensure equitable health surveillance.

### Limitations of the study

Our study has limitations shared with other population proteomics efforts.^48^ Compared to a first wave study,^18^ fewer members of the public participated (20% vs. 55%), attributed to a wider range of COVID-19 countermeasures and adaptation to the pandemic situation. Like during the first wave study, the participation rate in the current study is higher for women than men. While adjusted for in the proteomic analyses, this could cause issues with the generalizability of AAb analyses where there are known sex differences. The age-dependent rollout of vaccines in Sweden meant that many proteins associated with serostatus were also associated with the age of the participant. Still, some proteins showed no age association and several of the identified proteins have previously been reported in the context of COVID-19. Our study appears underpowered compared to other large-scale population proteomics efforts, such as the UK Biobank project.^27^ Still, coordinated sampling of random individuals from two geographically distant locations outside of the healthcare system has limited collider bias of our enrollment. Population studies using DBS and proteomics readout systems have seen a growing interest, but possibilities to replicate observations elsewhere remain rare. Previous or other infections, chronic diseases or health conditions, socioeconomic factors, misinterpretations or correctness of the provided information can influence our study. Other infections are likely to affect levels of inflammation-related proteins, complicating the immune phenotype interpretation. Due to the anonymity of the donors in our citizen survey, clinically important donor-related information about the overall health status, BMI, genetics, lifestyle, smoking, co-morbidities or medication remained unavailable to further interpret and define the immune phenotypes.

## Resource availability

### Lead contact

Further information and requests for resources in the current study should be directed to and will be addressed by the lead contact, Jochen M. Schwenk (jochen.schwenk@scilifelab.se).

### Materials availability

DBS samples cannot be shared due to the limited available material.

### Data and code availability

• Data supporting the findings of this study are available at SciLifeLab Data Repository (figshare https://doi.org/10.17044/scilifelab.28902911). The data are under restricted access because they represent sensitive individual-level human data. Via the lead contact, access to the normalized data can be granted for non-commercial validation purposes upon reasonable request to the corresponding authors, which contain the name of principal investigator and host organization (legal entity), contact details, and the scientific purpose of the data access request. We require that requests include commitments to acknowledge us when the data have been used in future publications and that the provided data will be stored securely, not hosted publicly elsewhere, or shared with other organizations other than the one requesting access.
• Codes used for the analysis and visualizations are deposited and publicly available on GitHub and can be accessed and cited via Zenodo (https://doi.org/10.5281/zenodo.15356855).
• Any additional information required to reanalyze the data reported in this paper is available from the lead contact upon request.

## Acknowledgments

First, we thank all anonymous volunteers who provided blood samples and answered the questionnaire for our study. We thank Matilda Dale, the Schwenk and Nilsson Labs members for support and fruitful discussions. We thank Ben Murrell (Karolinska Institutet) for providing help and code for normalizing the serology data, and August Jernbom Falk for comments on the manuscript. We acknowledge the tremendous support from the SciLifeLab infrastructure units for Affinity Proteomics in Stockholm and Uppsala and the Autoimmunity and Serology Profiling unit. We thank the KTH node of Protein Production Sweden (PPS), a national research infrastructure funded by the Swedish Research Council), the Human Secretome Project at the 10.13039/501100020625Wallenberg Center for Protein Research, and everyone at the Human Protein Atlas (HPA). The authors acknowledge support from the 10.13039/100009892National Genomics Infrastructure funded by 10.13039/501100009252Science for Life Laboratory, the 10.13039/501100004063Knut and Alice Wallenberg Foundation and the Swedish Research Council, and NAISS/Uppsala Multidisciplinary Center for Advanced Computational Science for assistance with massively parallel sequencing and access to the UPPMAX computational infrastructure. We thank the teams at Olink Proteomics and Alamar Bioscience. Funding was provided by the SciLifeLab National COVID-19 Research Program, financed by the 10.13039/501100004063Knut and Alice Wallenberg Foundation (2020.0182, 2020.0241); Sweden’s innovation agency Vinnova (2020-04451); SciLifeLab’s Pandemic Laboratory Preparedness program (VC-2022-0028); The 10.13039/100007436Erling Persson Foundation (20210125); and 10.13039/501100004359Swedish Research Council (2022-06340). We acknowledge support from the 10.13039/501100004063Knut and Alice Wallenberg Foundation for funding the Human Protein Atlas. This work was partially supported by the Wallenberg AI, Autonomous Systems and Software Program (WASP) funded by the 10.13039/501100004063Knut and Alice Wallenberg Foundation.

## Author contributions

Conceptualization, O.B., A.T.N., M.G., N.R., and J.M.S.; methodology, J.M.S. and N.R.; investigation, A.B. and H.A.; data curation, L.D., A.B., and V.A.; formal analysis, L.D., A.B., M.B.A., V.A., S. Björkander., S.K.M., and S. Bauer; visualization, L.D., A.B., M.B.A., and J.M.S.; resources, M.K., A.M., and C.F.; supervision, J.M.S. and N.R.; writing – original draft, L.D. and J.M.S.; writing – review and editing, all authors.

## Declaration of interests

O.B. is a co-founder and shareholder of Capitainer AB. E.M. has, outside the submitted study, received advisory board fees from ALK and AstraZeneca and lecture fees from Chiesi and Sanofi. N.R. is a co-founder and shareholder of the microsampling companies, including Capitainer AB and Samplimy Medical AB, and an inventor of several patents on microsampling solutions. J.M.S. is scientific advisor for ABC Labs and has, unrelated to this work, received travel and/or speaker support from Olink Bioscience, Alamar Bioscience, Illumina, Oxford Nanopore, and Luminex, and via KTH conducted contract research for Capitainer and Luminex.

## STAR★Methods

### Key resources table

**Table:** 

REAGENT or RESOURCE	SOURCE	IDENTIFIER
**Chemicals, peptides, and recombinant proteins**		

cOmplete™ Protease Inhibitor Cocktail	Roche	Cat# 04693116001
TWEEN® 20	Sigma-Aldrich	Cat# P1379
Phosphate Buffered Saline (PBS)	Thermo Fisher Scientific	Cat# 11330439
Bovine Serum Albumin, Cohn fraktion V (pH 7)	Saveen-Werner	Cat# B2000-500
Skim milk powder for microbiology	Sigma Aldrich	Cat# 70166-500G
F(ab')2-Goat anti-Human IgG Fc Secondary Antibody, RPE conjugated	Thermo Fisher Scientific/Invitrogen	Cat# H10104
Sodium phosphate monobasic, BioReagent, for molecular biology, anhydrous, ≥98%	Sigma	Cat# S3139-250G
Blocking Reagent for ELISA	Roche	Cat# 11112589001
ProClin™ 300, pkg of 50 mL	Sigma	Cat# 48912-U
MES hydrate (2[N-Morphalino]ethanesulfonic acid)	Sigma	Cat# M2933-25G
Ethylene Dichloride (EDC), customized 65mg/vial	Proteochem	Cat# c100-65mg
500 MG SULFO-NHS 500MG	Thermo Fisher Scientific	Cat# 10391314
SARS-CoV-2 Spike S1, domain (S1)	KTH, The Protein Factory	DOI: https://doi.org/10.1002/cti2.1312^4^
SARS-CoV-2 Spike S, foldon (S1S2)	KTH, The Protein Factory	DOI: https://doi.org/10.1002/cti2.1312^4^
SARS-CoV-2 Spike S1, receptor-binding domain (RBD)	KTH, The Protein Factory	DOI: https://doi.org/10.1002/cti2.1312^4^
SARS-CoV-2 Nucleocapsid (Na)	Acro Biosystems	Cat# NUN-C5227
SARS-CoV-2 Nucleocapsid, C-terminal (Nc)	KTH, The Protein Factory	DOI: https://doi.org/10.1002/cti2.1312^4^
Epstein–Barr nuclear antigen 1 (EBNA1)	Abcam	Cat# ab138345
Goat anti-human IgA	Immune Systems Ltd	Cat# GA-80A
Rabbit anti-human IgG	Jackson ImmunoResearch	RRID:AB_2339630
Goat anti-human IgM	Jackson ImmunoResearch	RRID:AB_2337543
IFNA1	KTH, The Protein Factory	DOI: https://doi.org/10.1016/j.nbt.2020.05.002^49^
IFNA2	KTH, The Protein Factory	DOI: https://doi.org/10.1016/j.nbt.2020.05.002^49^
IFNA3	KTH, The Protein Factory	DOI: https://doi.org/10.1016/j.nbt.2020.05.002^49^
IFNA4	KTH, The Protein Factory	DOI: https://doi.org/10.1016/j.nbt.2020.05.002^49^
IFNA5	KTH, The Protein Factory	DOI: https://doi.org/10.1016/j.nbt.2020.05.002^49^
IFNA6	KTH, The Protein Factory	DOI: https://doi.org/10.1016/j.nbt.2020.05.002^49^
IFNA7	KTH, The Protein Factory	DOI: https://doi.org/10.1016/j.nbt.2020.05.002^49^
IFNA8	KTH, The Protein Factory	DOI: https://doi.org/10.1016/j.nbt.2020.05.002^49^
IFNA10	KTH, The Protein Factory	DOI: https://doi.org/10.1016/j.nbt.2020.05.002^49^
IFNA14	KTH, The Protein Factory	DOI: https://doi.org/10.1016/j.nbt.2020.05.002^49^
IFNA16	KTH, The Protein Factory	DOI: https://doi.org/10.1016/j.nbt.2020.05.002^49^
IFNA17	KTH, The Protein Factory	DOI: https://doi.org/10.1016/j.nbt.2020.05.002^49^
IFNA21	KTH, The Protein Factory	DOI: https://doi.org/10.1016/j.nbt.2020.05.002^49^
IFNAR1	KTH, The Protein Factory	DOI: https://doi.org/10.1016/j.nbt.2020.05.002^49^
IFNAR2	KTH, The Protein Factory	DOI: https://doi.org/10.1016/j.nbt.2020.05.002^49^
IFNG	KTH, The Protein Factory	DOI: https://doi.org/10.1016/j.nbt.2020.05.002^49^
IFNGR2	KTH, The Protein Factory	DOI: https://doi.org/10.1016/j.nbt.2020.05.002^49^
IFNL1	KTH, The Protein Factory	DOI: https://doi.org/10.1016/j.nbt.2020.05.002^49^
IFNL2	KTH, The Protein Factory	DOI: https://doi.org/10.1016/j.nbt.2020.05.002^49^
IFNL3	KTH, The Protein Factory	DOI: https://doi.org/10.1016/j.nbt.2020.05.002^49^
IFNLR1	KTH, The Protein Factory	DOI: https://doi.org/10.1016/j.nbt.2020.05.002^49^
IFNW1	KTH, The Protein Factory	DOI: https://doi.org/10.1016/j.nbt.2020.05.002^49^

**Biological samples**		

Dried blood spots	This study	NA

**Critical commercial assays**		

Olink Explore 384 Inflammation Panel I	Olink	Lot 23405
NULISAseq™ Inflammation Panel 250	Alamar Biosciences	Cat# 800103

**Deposited data**		

Proteomics data	This study	Figshare: https://doi.org/10.17044/scilifelab.28902911

**Software and algorithms**		

R	The R Project for Statistical Computing	Version 4.3.2
Julia	The Julia Project	Version 1.7.2
Analysis code	This study	Zenodo: https://doi.org/10.5281/zenodo.15356855

### Experimental model and study participant details

Home-sampling kits (MM18-01-001, Capitainer AB, Sweden) were sent out to 2000 randomly chosen individuals (ages 18-69) from metropolitan Stockholm and Gothenburg (1000 each) as previously described.^18^^,^^20^ Anonymous participants were invited to volunteer in capillary blood sampling (following the provided instructions) by pricking a finger and collecting blood drops on a quantitative dried blood spot (DBS) sampling card. Participants also filled out a questionnaire with health-related questions and a consent form. Sampling cards, questionnaires, and consent forms were returned by regular mail, and the cards were stored at room temperature until extraction. For the analyses, 437 cards were used, with age and sex distributions of the donors shown in Table 1 and Figure 1. Sampling frequency was higher among female donors. The study was approved by the regional ethical board (EPN Stockholm, Dnr 2015/867-31/1) and the Swedish Ethical Authority (EPM, Dnr 2021-01106 and Dnr 2023-03016-02).

### Method details

#### Sample preparation

The DBS cards were eluted to obtain samples for analysis as previously described.^18^^,^^20^ In brief, the cards were first inactivated by heating in an oven (UN55m, Memmert GmbH) at 56°C for 60 minutes. The inactivated discs containing blood were placed in a flat-bottom 96-well plate (#734-2327, VWR) and treated with 100 μL PBS with 0.05% Tween20 (#97062-332, VWR) and protease inhibitor cocktail (#04693116001, Roche). The plates were shaken gently at 170 rpm at room temperature for 60 min and then centrifuged (2095 rcf, Allegra X-12R, Beckman Coulter Inc) at 3000 rpm for 3 min before collecting 70 μL of supernatant in a PCR plate (#732-4828, VWR). The eluates were stored at -20°C until analysis.

#### Serological assays

Serological assays were performed on samples from 437 participants who successfully completed the blood spot collection and questionnaire. IgG antibodies against the nucleocapsid (N), spike (S and RBD) proteins of the SARS-CoV-2 virus, as well as against several human interferons, were measured using suspension bead arrays (SBAs). The antigens that were used are shown in Table 2 and the Key Resources Table. The assays were performed as previously described.^18^^,^^20^ In brief, target proteins were covalently linked to colour-coded magnetic beads (MagPlex, Luminex Corp.) with NHS/EDC coupling, and the beads were combined to form an antigen bead array. DBS eluates were diluted 2.5x with assay buffer (PBST with 3% BSA and 5% milk powder), and 35 μL of each was combined with a 5 μL antigen bead array and incubated at room temperature for 60 min. The beads were analysed using a Luminex FlexMap 3D instrument (Luminex Corp) with anti-human IgG-R-PE (Jackson ImmunoResearch) for detection. Measurements were reported as median fluorescence intensity (MFI) values per antigen and sample, where each data point had at least 32 events per bead ID.

#### Autoreactive antibody assays

In addition to the COVID-19 antigens, we included 22 human interferons (IFNs) in the bead mixture of the SBAs, see Table S1. The IFNs were produced as described elsewhere.^50^ Protein coupling, analysis, and readout were conducted as described for SARS-CoV-2 proteins above. Since IFN assays report the levels of auto-reactive antibodies (AAbs) against different interferons, we conducted the data analysis separately. The eluates were stored at -80°C until further analysis.

#### Proteomics assays

To measure a large number of low-abundant and disease-relevant circulating inflammatory proteins for comparison with the serology, samples were sent to SciLifeLab’s Affinity Proteomics Unit (Uppsala, Sweden) for the proximity extension assay (PEA) using Olink Explore,^33^ and to Alamar Biosciences Inc. (Fremont, California, USA) for the NULISAseq assay.^34^

#### Proximity extension assay

The samples were processed with Olink Explore 384 Inflammation panel I (Lot B23405, Olink Proteomics AB, Uppsala, Sweden), which is based on Proximity Extension Assay (PEA)^33^ and allows for simultaneous detection of up to 368 proteins using 1 μL of DBS eluate. In brief, two matched antibodies labelled with unique complementary oligonucleotides bind to a target protein in solution, which brings the oligonucleotides in proximity and allows them to hybridise and extend to form a double-stranded DNA barcode unique to the target protein. The subsequent library preparation adds sample indices and sequencing adapters, enabling multiplexing and readout with next-generation sequencing. The manufacturer’s instructions were followed, except that Olink and Science for Life Laboratory in Stockholm collaborated to enable the Agilent Bravo (Agilent Technologies, Santa Clara, CA, USA) automated liquid handler to be used in the workflow. The libraries were sequenced on a NovaSeq 6000 (Illumina, San Diego, CA, USA) at the Science for Life Laboratory in Uppsala, Sweden, according to Olink instructions. The data are reported as Normalised Protein eXpression (NPX) values, semi-quantitative arbitrary units (AU) on a log2 scale.

#### NULISAseq assay

NULISAseq assays were performed at Alamar Biosciences, Fremont, USA, as described previously.^34^ Briefly, DBS samples were shipped on dry ice, stored at -80°C, thawed on ice, and centrifuged at 10,000g for 10 mins. Then, 10 μL supernatant samples were plated in 96-well plates and analysed with Alamar’s Inflammation Panel 250 (#800103), targeting mostly inflammation and immune response-related cytokines and chemokines. A Hamilton-based automation instrument was used to perform the NULISAseq workflow, starting with immunocomplex formation with DNA-barcoded capture and detection antibodies, followed by capturing and washing the immunocomplexes on paramagnetic oligo-dT beads, then releasing the immunocomplexes into a low-salt buffer, which were captured and washed on streptavidin beads. Finally, the proximal ends of the DNA strands on each immunocomplex were ligated to generate a DNA reporter molecule containing target-specific and sample-specific barcodes. DNA reporter molecules were pooled and amplified by PCR, purified, and sequenced on Illumina NextSeq 2000.

Sequencing data were processed using the NULISAseq algorithm (Alamar Biosciences). The sample- (SMI) and target-specific (TMI) barcodes were quantified, and up to two mismatching bases or one indel and one mismatch were allowed. Intraplate normalisation was performed by dividing the target counts for each sample well by that well’s internal control counts. Inter-plate normalisation was then performed using inter-plate control (IPC) normalisation, wherein counts were divided by target-specific medians of the three IPC wells on that plate. Data were then rescaled, add 1 and log2 transformed to obtain NULISA Protein Quantification (NPQ) units for downstream statistical analysis.

### Quantification and statistical analysis

Data processing, analysis and visualisation was performed with the R (version 4.3.2)^51^ and Julia (version 1.7.2)^52^ programming languages and the tidyverse (version 2.0.0),^53^ targets (version 1.4.1),^54^ and tarchetypes (version 0.7.12)^55^ R packages. Plots were created using either the ggplot2 package (version 3.5.1)^56^ together with the patchwork (version 1.3.0.9000),^57^ RColorBrewer (version 1.1.3),^58^ ggh4x (version 0.2.8),^59^ ggnewscale (version 0.4.10),^60^ ggbeeswarm (version 0.7.2),^61^ ggrepel (version 0.9.5),^62^ ggsignif (version 0.6.4),^63^ ggbiplot (version 0.6.2),^64^ GGally (version 2.2.1)^65^ and ggvenn (version 0.1.10) packages,^66^ or the ComplexHeatmap (version 2.18.0)^67^ and circlize (version 0.4.15)^68^ packages. The table1 package (version 1.4.3)^69^ was used to generate some tables. A False Discovery Rate (FDR) value of 0.05 was used as threshold for statistical significance if not stated otherwise. Tests used and group sizes are stated in figures and associated legends and supplementary tables.

#### Serological data analysis

As described previously,^18^ data from the serological assay were normalised to remove correlation with negative control samples using a mixed model programmed in Julia. The model was fit on log2-transformed data and consisted of a linear and uniform distribution model to account for distributions where most samples were at low baseline levels while a few were at higher positive levels. The resulting relative log2 values were scaled and centred per protein to put them on the same scale. An overview of the antibody data is given in Data S2.

Seropositivity of each antibody was determined using a population-based threshold at 6 SD (for anti-SARS-CoV-2 Abs) or 12 SD (for IFN AAbs) of the estimated negative proportion (uninfected individuals for N and IFNs, uninfected and unvaccinated individuals for S, RBD) above the gaussian population peak as described previously.^18^ Attained seropositivity classifications for IFN AAbs were associated with questionnaire data and anti-SARS-CoV-2 Ab classifications using the Fisher exact test.

Hierarchical clustering was used on the N, S, and RBD MFI data to stratify samples based on serology. The resulting clustering was cut into four seroclusters that were compared to vaccination and infection status from the questionnaire. Mismatch in questionnaire data and seroclusters regarding vaccination were tested using the Fisher exact test. The clustering was evaluated with Lasso regularised logistic or multinomial regression using the collection of functions in the tidymodels package (version 1.1.1.)^70^ to predict questionnaire traits and seroclusters using anti-SARS-CoV-2 Ab levels. 70% of the samples were used as a training set, and the remaining 30% as a hold-out test set. The penalty was tuned via five-fold cross-validation in the training set. The test set was used to evaluate the area under the curve (AUC) of the receiver operating curve (ROC) for each trait.

#### Proteomics data analysis

Normalised protein measurements were received from Olink and Alamar. To reduce sample-to-sample variation stemming from sample handling, protein measurements were normalised with the ProtPQN method using the ProtPQN package (version 1.0.1).^71^ Deviating samples were detected by plotting sample medians and interquartile ranges (IQRs). Samples falling outside of 3 SDs of either axis were excluded from downstream analysis. Confounding from sex differences was removed using a linear model. An overview of the proteomics data is given in Data S3. The data were plotted using the principal component analysis (PCA) dimensionality reduction technique to visually check for trends in the data.

To compare circulating proteins with serology, proteins were associated with seropositivity classifications of anti-SARS-CoV-2 Abs and IFN AAbs using logistic regression with the seropositivity as an outcome. The proteins were also associated with the seroclusters using the Kruskal-Wallis test and, if FDR <0.05, the Wilcoxon rank-sum test for pair-wise comparisons. Furthermore, to investigate the predictive power of proteins on age, sex, region, and immune status defined by the questionnaire and the seroclustering, Lasso regularised logistic or multinomial regression was performed similarly to the serology.

Clustering was performed on the proteomics data to find protein-centric sample groups. Unlike the low-dimensional serology data analysis, the proteomics data dimensions were reduced using weighted gene correlation network analysis (WGCNA) using the WGCNA R package (version 1.72-5).^72^ The pickSoftThreshold function was used to pick a soft threshold of 2 where the scale-free topology R^2^ reached a plateau above 0.8 for a scale-free distribution, and WGCNA was performed using the blockwiseModules function with default parameters. The resulting protein module eigengenes (MEs, apart from the grey module containing non-correlated proteins) were used to cluster the samples with hierarchical clustering where the gap statistic was used to determine the optimal number of clusters (cluster package version 2.1.4^73^). The cluster stability was evaluated by calculating the mean Jaccard index for each cluster with the clusterboot function of the fpc package (version 2.2-11).^74^ Any clusters with a mean Jaccard index below 0.5 or a size below 10 were aggregated into a noise cluster. The resulting clusters, henceforth called proteotypes, were numbered from 0 (noise) to 5.

Associations between WGCNA protein modules and population traits and diseases were investigated by integrating previously reported UK Biobank associations.^27^ The proportions of significantly associated proteins for each module and trait were compared to those in the rest of the samples using the Fisher exact test. For HPA, the proportions of proteins from each module were plotted for each immune cell type.

## Supplemental information

Document S1. Figures S1–S16

Data S1. The questions sent out together with sampling devices for health information, related to STAR Methods

Data S2. Boxplots showing the distributions of relative antibody titers from multianalyte serology assays with seropositive samples marked in blue

Data S3. Boxplots showing the distributions of normalized protein levels from affinity proteomics assays
