# Supplementary material for: Exploration of immune phenotypes in self-sampling citizens
Source: iScience. 2026 Jan 3;29(2):114611. doi: 10.1016/j.isci.2025.114611 (PMC12860695; doi:10.1016/j.isci.2025.114611)
Supplement: Document S1. Figures S1–S16 [file mmc1.pdf]

iScience, Volume *29*

Supplemental information

Exploration of immune phenotypes in self-sampling citizens

Leo Dahl, Annika Bendes, María Bueno Álvez, Vincent Albrecht, Hooman Aghelpasand, Sophia Björkander, Simon Kebede Merid, Anja Mezger, Max Käller, Claudia Fredolini, Åsa Torinsson Naluai, Olof Beck, Erik Melén, Stefan Bauer, Magnus Gisslén, Niclas Roxhed, and Jochen M. Schwenk

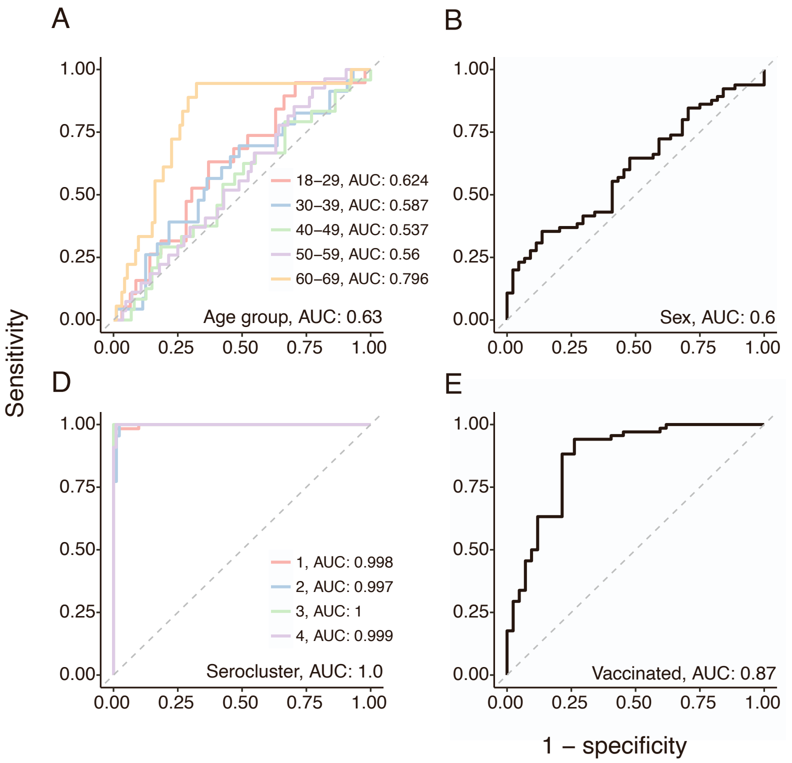

Fig S1: Prediction of self-reported traits and seroclusters from serology data. (A) Age (pink: 18-29; blue: 30-39; green: 40-49; purple: 50-59; orange: 60-69 years old), **(B)** sex, **(C)** sampling region, **(D)** serocluster membership (pink: serocluster 1; blue: 2; green: 3; purple: 4), **(E)** vaccination (binary), and **(F)** infection were predicted using levels of circulating anti-SARS-CoV-2 antibodies. Receiver operating characteristic (ROC) curves and area under the curves (AUCs) are obtained from Lasso logistic (binary outcome) or multinomial regressions using anti-SARS-CoV-2 Ab levels. The AUC displayed for age and seroclusters are the Hand Till average AUCs. See Table S2 for variable importances.

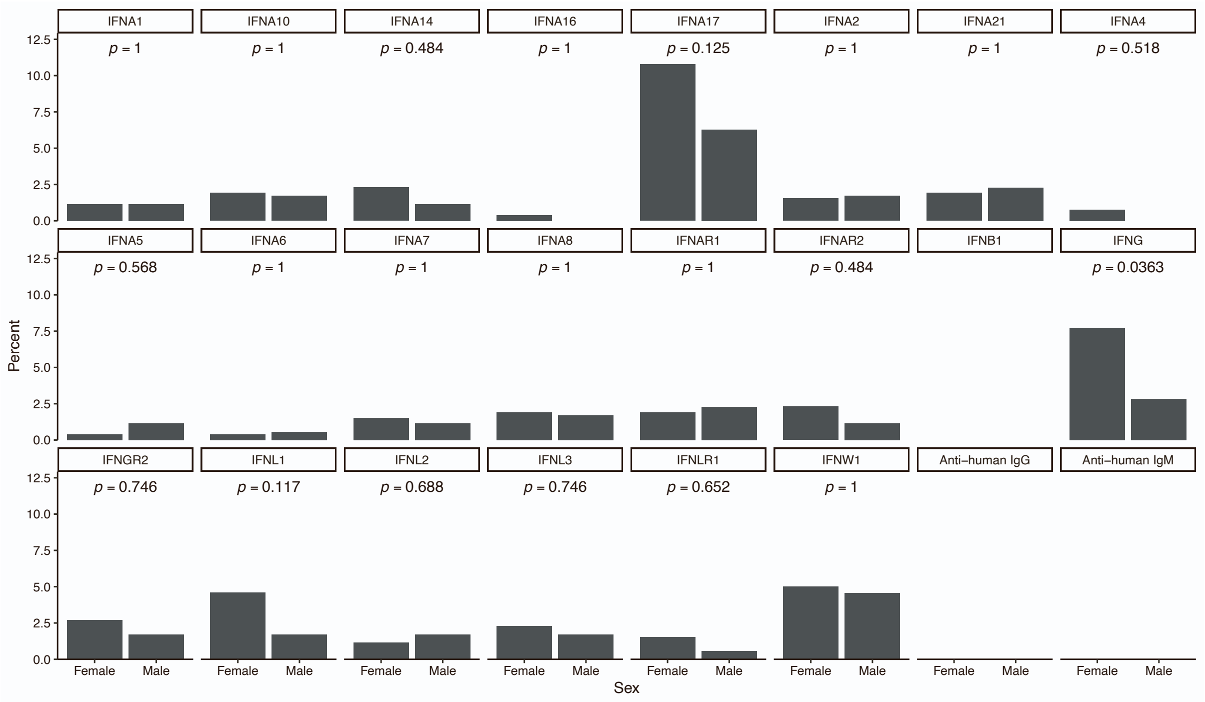

Fig S2: Sex differences in anti-IFN autoantibody (AAb) frequencies. Percentages of female and male individuals that are positive for different anti-IFN AAbs and control anti-human IgG and IgM. *P*-values are from Fisher exact tests. Group sizes are 260 (Female) and 175 (Male) (Table 1).

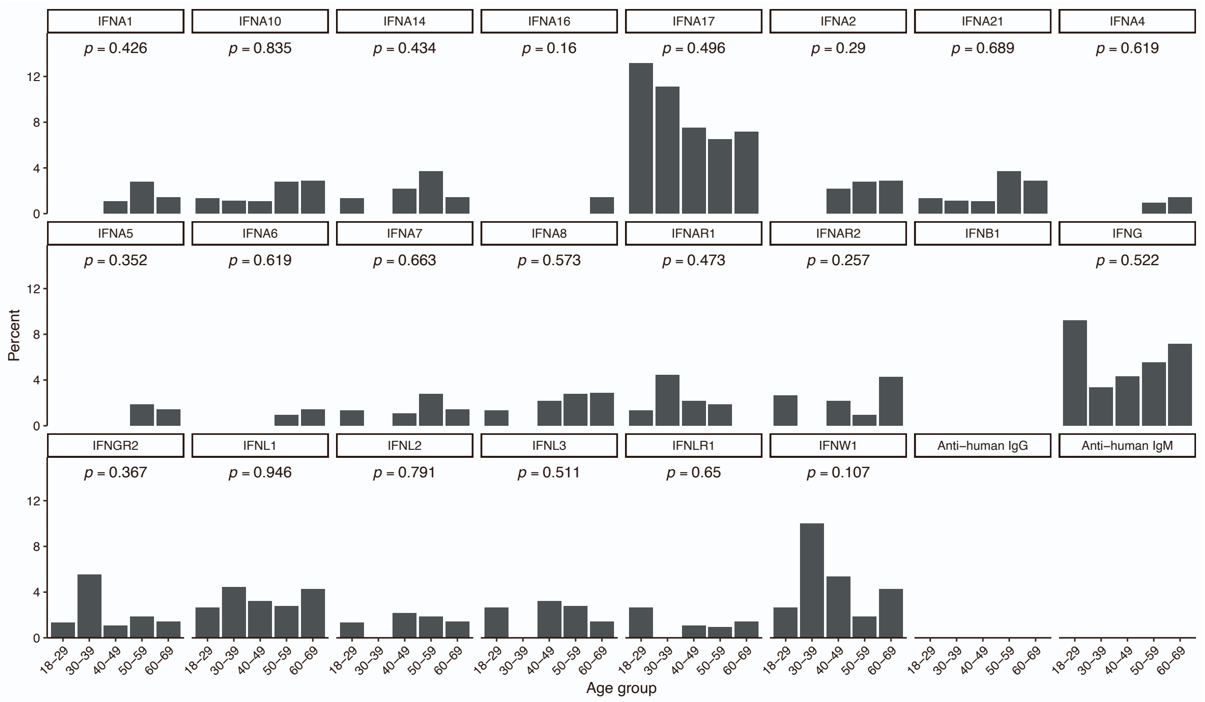

Fig S3: Age differences in anti-IFN autoantibody (AAb) frequencies. Percentages of samples from different age groups that are positive for different anti-IFN AAbs and control anti-human IgG and IgM. *P*-values are from Fisher exact tests. Group sizes are 76 (18-29), 90 (30-39), 93 (40-49), 108 (50-59), and 70 (60-69) (Table 1).

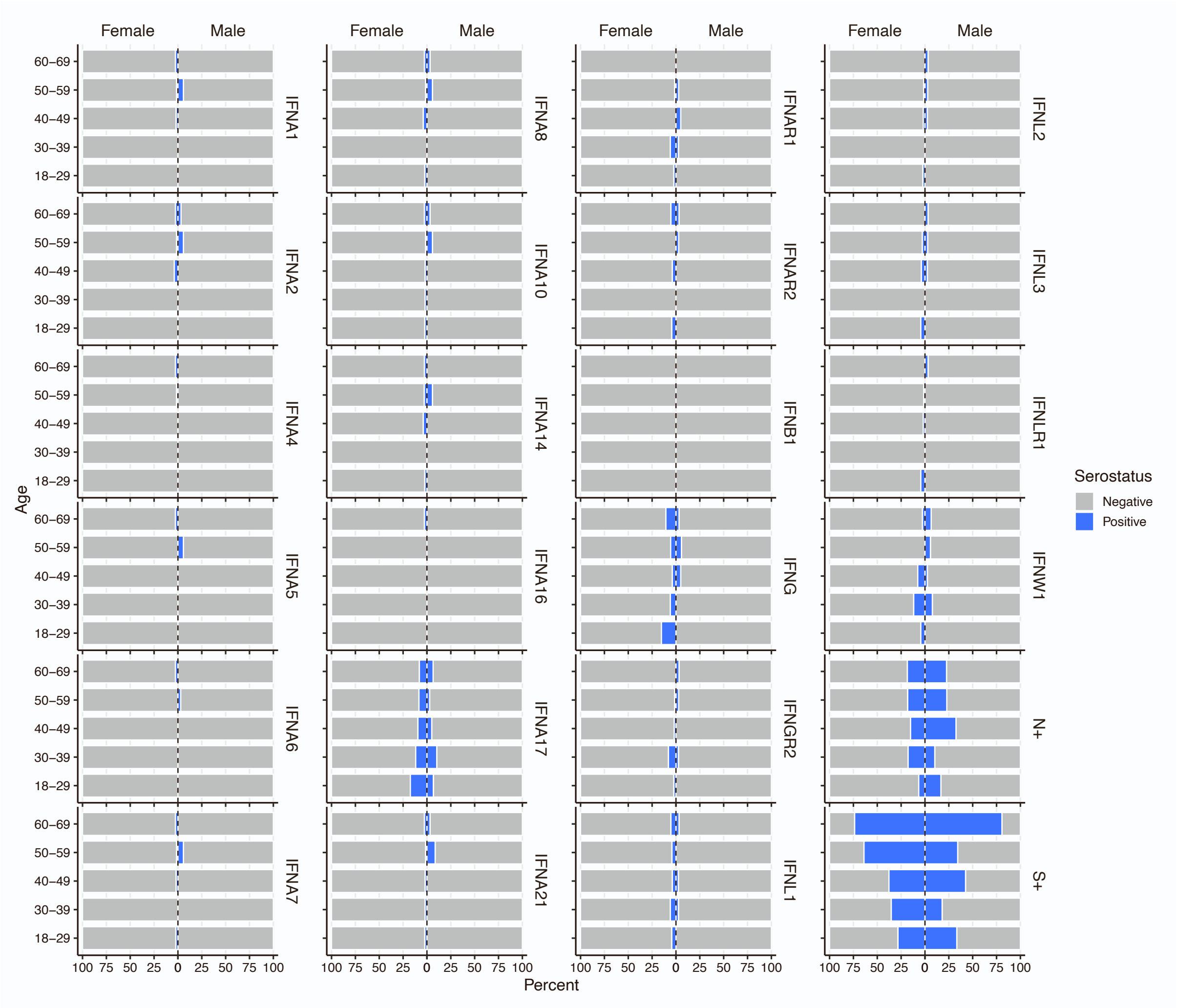

Fig S4: Differences in anti-IFN autoantibody (AAb) and anti-SARS-CoV-2 Ab frequencies by age and sex. Percentages of female and male individuals in each age group that are positive for different anti-human IFN AAbs or anti-SARS-CoV-2 Abs (blue: positive; grey: negative).

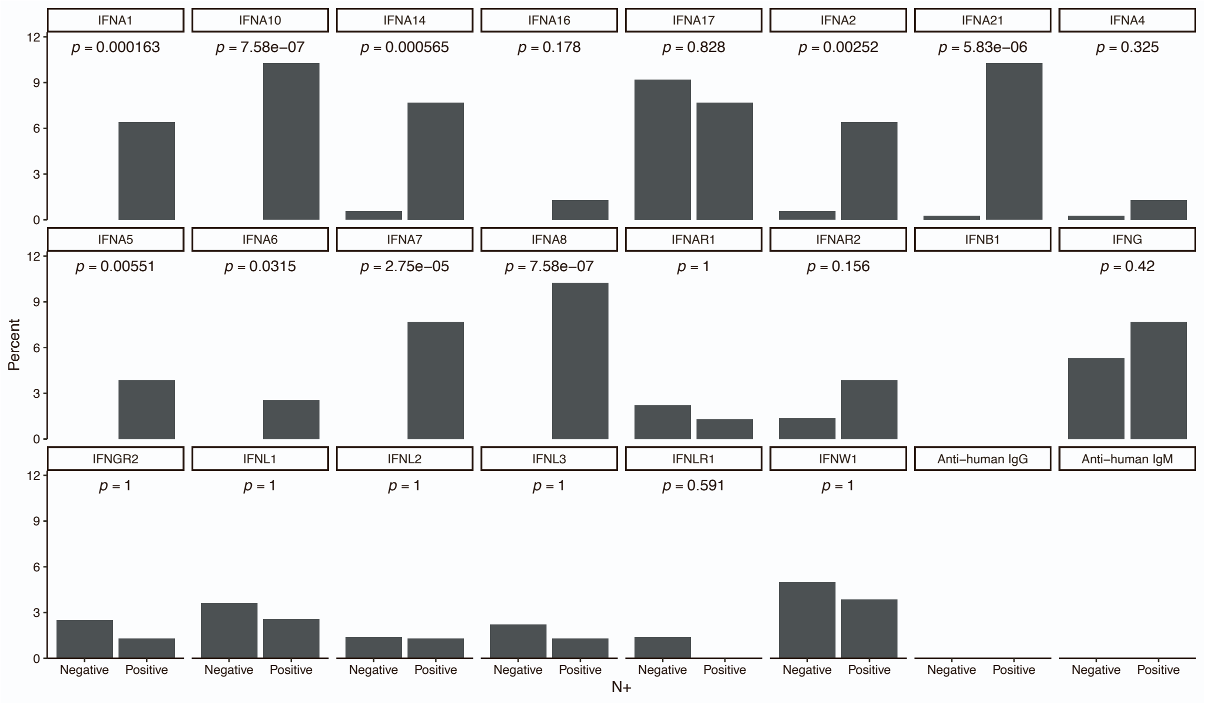

Fig S5: Differences in anti-IFN autoantibody (AAb) frequencies per N-seropositivity. Percentages of N+ and N- samples that are positive for different anti-IFN AAbs and control anti-human IgG and IgM. *P*-values are from Fisher exact tests. Group sizes are 359 (N-) and 78 (N+).

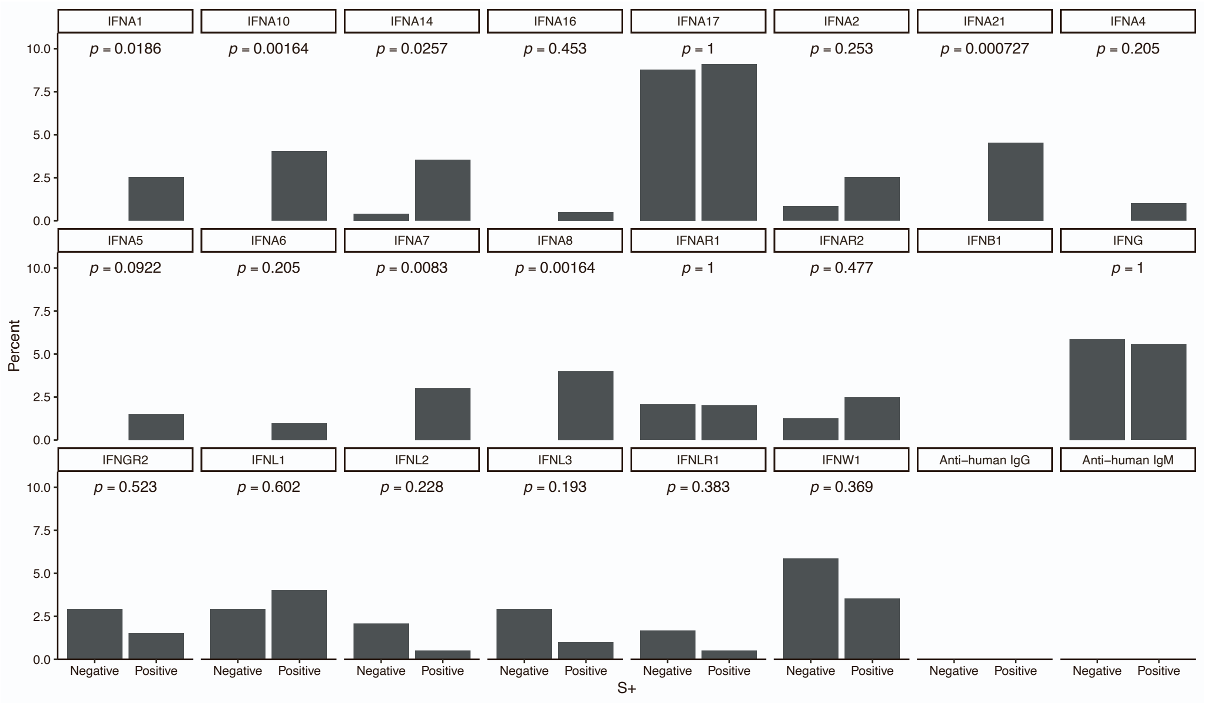

Fig S6: Differences in anti-IFN autoantibody (AAb) frequencies per S-seropositivity. Percentages of S+ and S- samples that are positive for different anti-IFN AAbs and control anti-human IgG and IgM. *P*-values are from Fisher exact tests. Group sizes are 239 (S-) and 198 (S+).

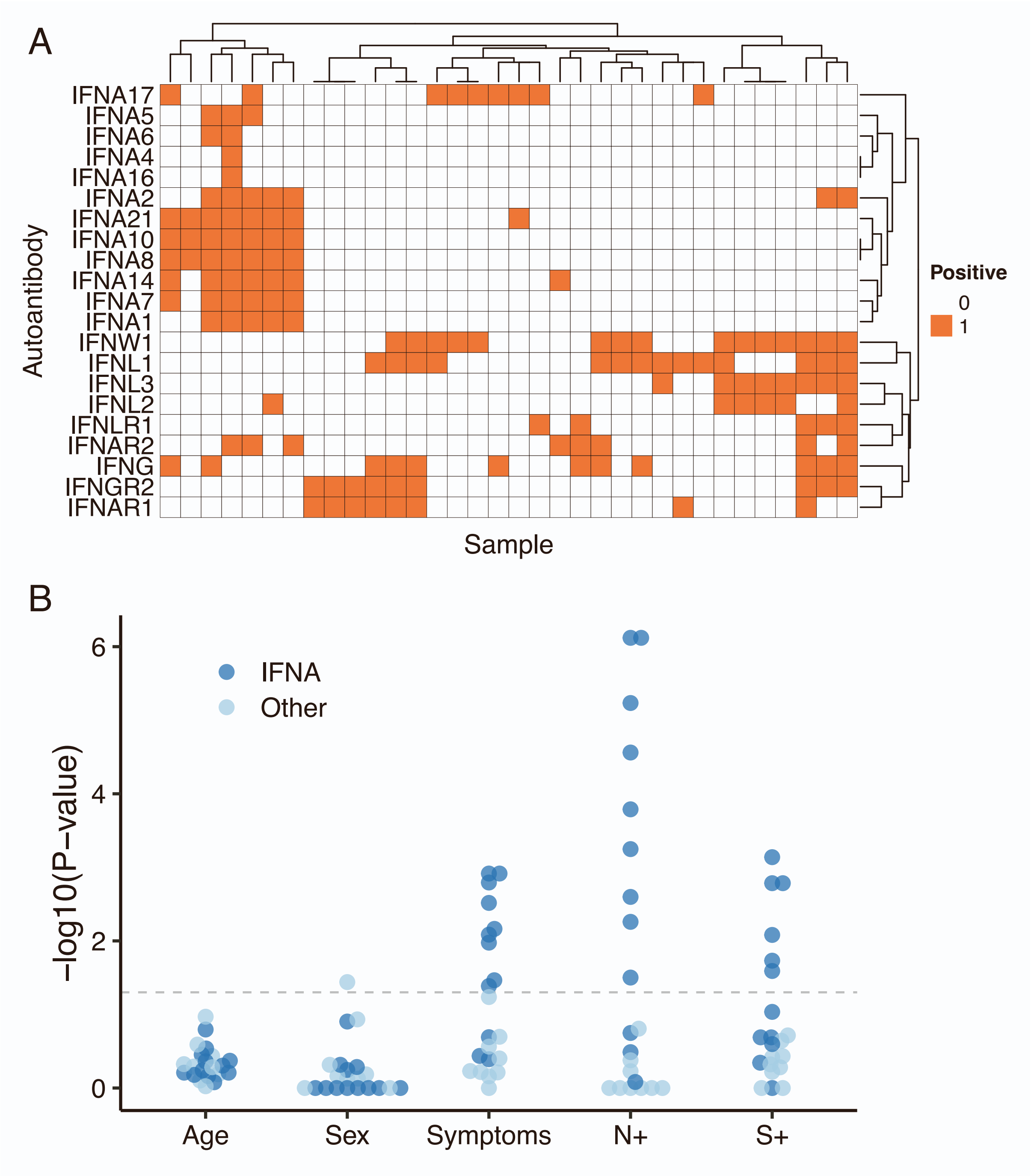

Fig S7: Anti-IFN autoantibody (AAb) co-occurrence and differences in frequencies across traits and proteins. (A) Anti-IFN AAb positivity patterns were clustered for samples positive for more than one anti-IFN AAb (red: seropositive; white: seronegative). IFNs from the interferon A family frequently co-occurred with each other. IFNL1, IFN2, and IFN3 were often together, along with IFNW. **(B)** Associations between interferon AAb positivity and age, sex, symptoms, and S and N seropositivity were tested using Fisher's exact test. Each point represents the -log10(*P-value)* of a test for a trait and an interferon. The point is dark blue if the antigen is of the interferon A family. The grey dashed line marks a P-value of 0.05. Groups and group sizes are shown in figures S2, S3, S5, and S6 and their legends, as well as table 1.

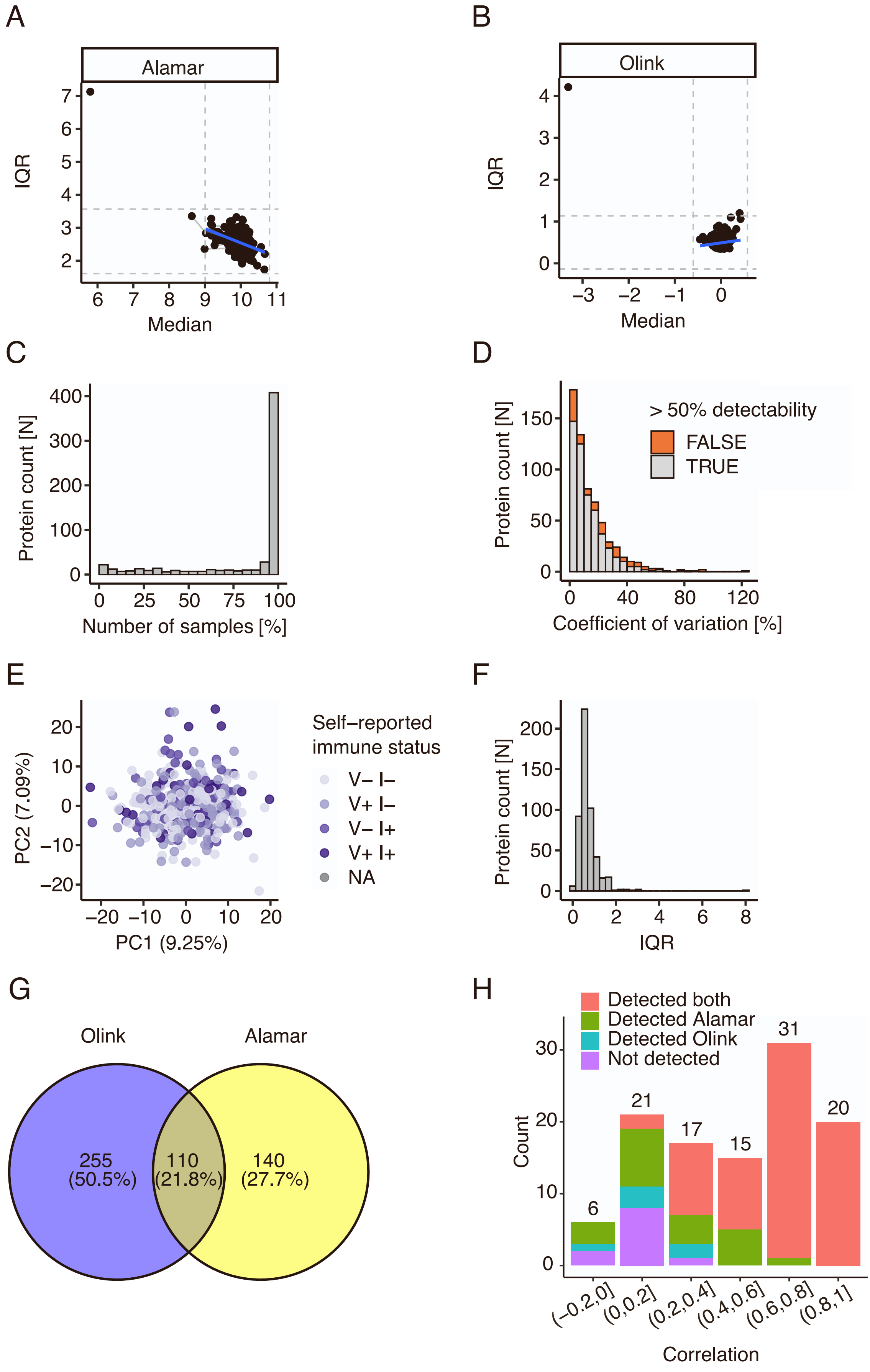

A
Alamar
IQR
Median
B
Olink
IQR
Median
C
Protein count [N]
Number of samples [%]
D
> 50% detectability
FALSE
TRUE
Protein count [N]
Coefficient of variation [%]
E
Self−reported immune status
V− I−
V+ I−
V− I+
V+ I+
NA
PC2 (7.09%)
PC1 (9.25%)
F
Protein count [N]
IQR
G
Olink
Alamar
255 (50.5%)
110 (21.8%)
140 (27.7%)
H
Detected both
Detected Alamar
Detected Olink
Not detected
Count
Correlation
(−0.2,0]
(0,0.2]
(0.2,0.4]
(0.4,0.6]
(0.6,0.8]
(0.8,1]

Fig S8: Quality control and comparison of the proteomics data, related to Fig 3**. (A-B)** Outlier sample detection in **(A)** Alamar and **(B)** Olink data by plotting the median and interquartile range (IQR) of each sample. A threshold is set at 3 standard deviations from the mean of the median and the IQR. The top left sample is the same on both platforms. The blue line shows the linear trends. **(C)** Protein detectability is plotted as the percent of samples where the measured protein level is above the limit of detection (LOD). **(D)** Coefficient of variation (CV%) for each protein. The proteins detected above LOD in > 50% of the samples are grey, while those detected less are coloured red. **(E)** Principal component analysis **(**PCA) of the proteomics data. Each point is a sample and is coloured by the self-reported vaccination and infection status. **(F)** IQR distribution of the proteins. **(G)** Venn diagram showing the number of proteins that overlap between Olink (blue) and Alamar (yellow) data. **(H)** Correlation between Olink and Alamar proteins that overlapped. A protein is marked as detected if its values are above the LOD in >50% of the samples (red: detected in both; green: detected in Alamar; turquoise: detected in Olink; purple: not detected in either). Generally, proteins that are well detected in both platforms correlate better.

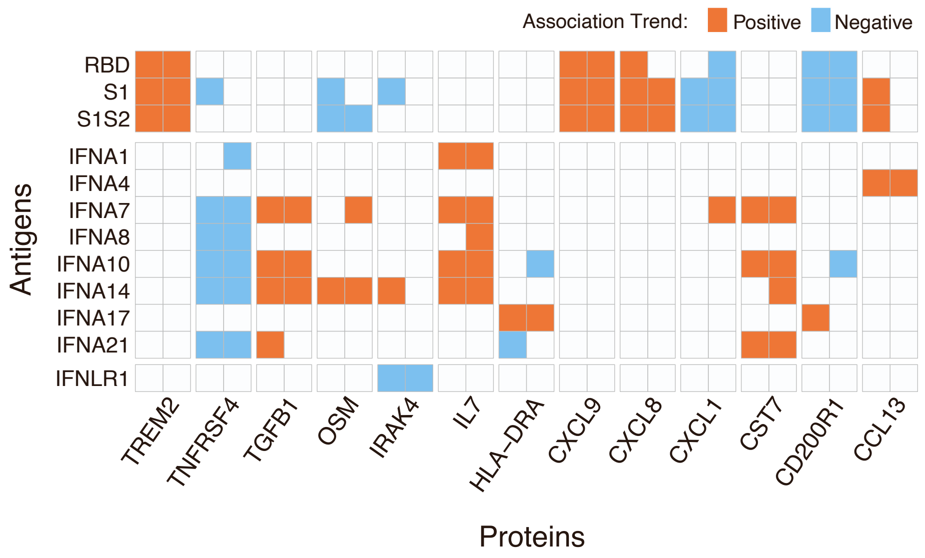

Fig S9. Relationships between circulating proteins and antibodies. Proteomics data from both platforms was used to associate circulating proteins with antibodies against SARS-CoV-2 antigens and human interferons. From logistic regression analyses, only association pairs with concordant trends and nominal *P*-values < 0.05 are described. Red cells refer to positive association trends, meaning higher protein levels in the antibody-positive groups, while blue cells refer to negative associations. Exact *P*-values for all proteins as well as numbers of seropositive and seronegative samples per antibody can be found in table S4.

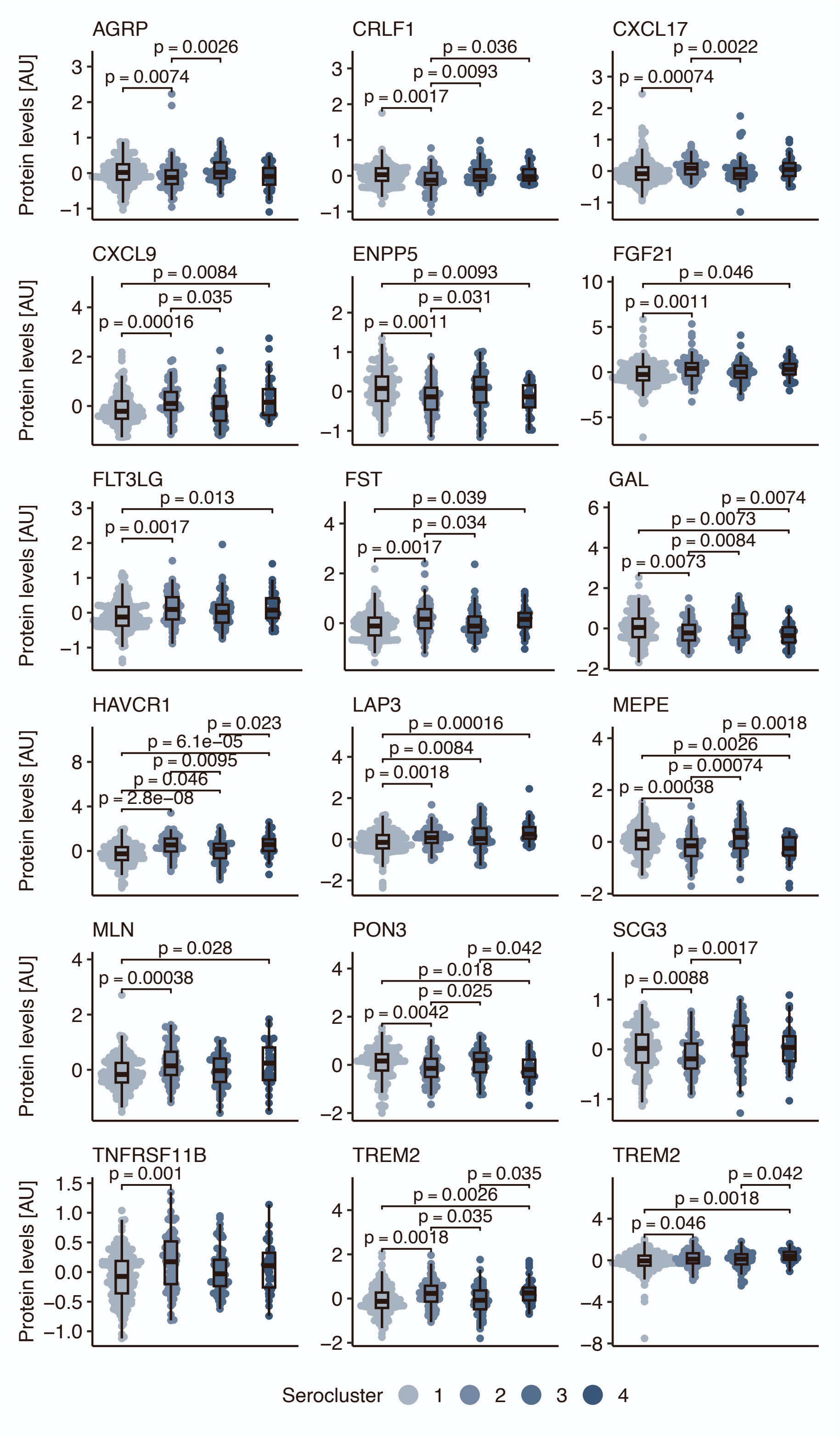

Fig S10. Linear associations between seroclusters and proteins. Proteins with significantly different levels between the seroclusters are shown. Seroclusters are depicted in shades of blue. The displayed *P*-values were obtained from the Wilcoxon rank-sum test and have been adjusted using the False discovery rate method. The center line in the boxes represents the median value and whiskers 1.5*IQR below and above the 25th and 75th percentiles, respectively. Group sizes are 208 (serocluster 1, light purple), 83 (cluster 2), 70 (cluster 3), and 34 (cluster 4, dark purple).

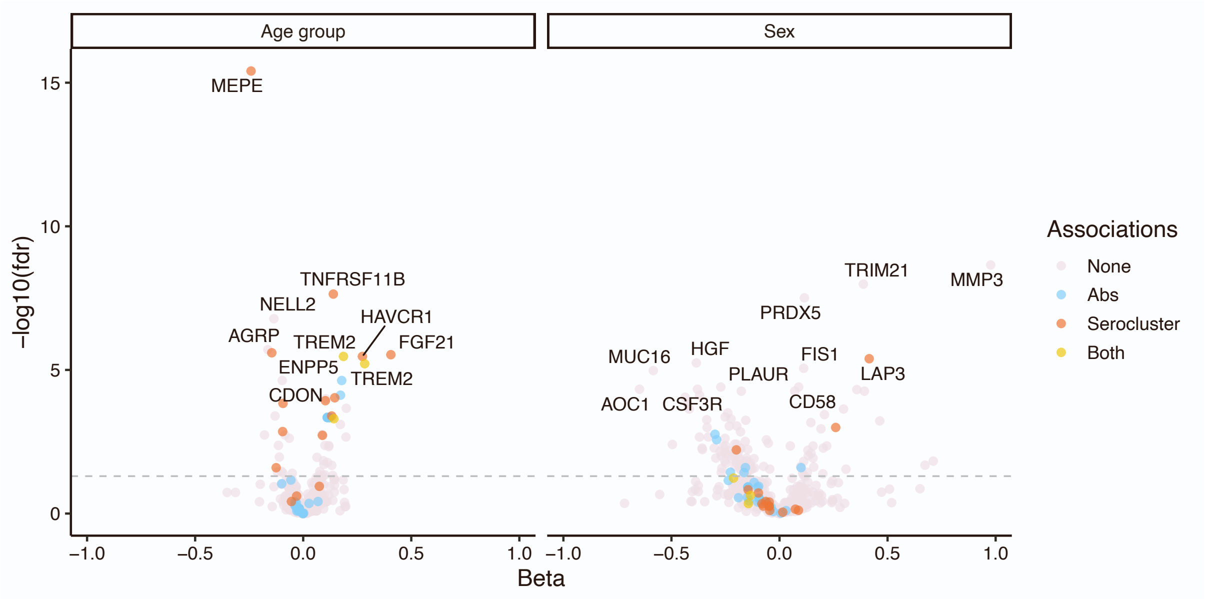

Fig S11: Age and sex association of proteins in the seronegative cluster. Volcano plots displaying the false discovery rate (FDR)-adjusted p-values for the linear associations between protein levels and age group or sex. The analysis was limited to individuals in serocluster 1 to reduce confounding of infection and vaccination. The 10 proteins with the lowest FDR values are labelled for each variable. Proteins are coloured by their associations with seroclusters (red, Fig S10) and concordant nominal associations with anti-SARS-CoV-2 Abs (blue, Fig S9) (or yellow for both). All nominal and adjusted *P*-values can be found in table S7.

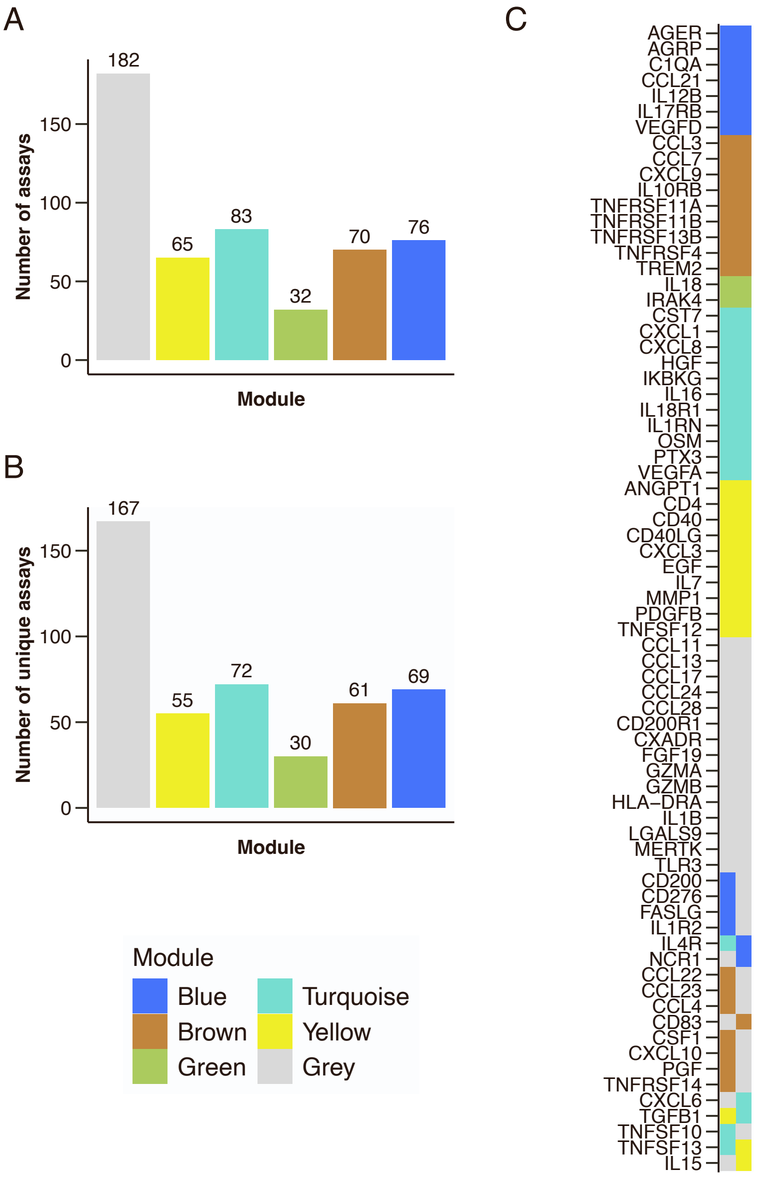

Fig S12: Weighted Gene Correlation Network Analysis (WGCNA) module protein numbers and proteins assigned to cross-platform protein assays, related to fig 4. **(A)** The number of assays assigned to each WGCNA module (coloured by their names, i.e. blue, brown, green, turquoise, yellow, and grey). **(B)** The number of unique assays assigned to each module. **(C)** Module assignment agreement for proteins measured by two platforms. Out of 73 proteins measured using two platforms, 54 (74%) were assigned to the same WGCNA module and 19 (26%) were assigned to different modules.

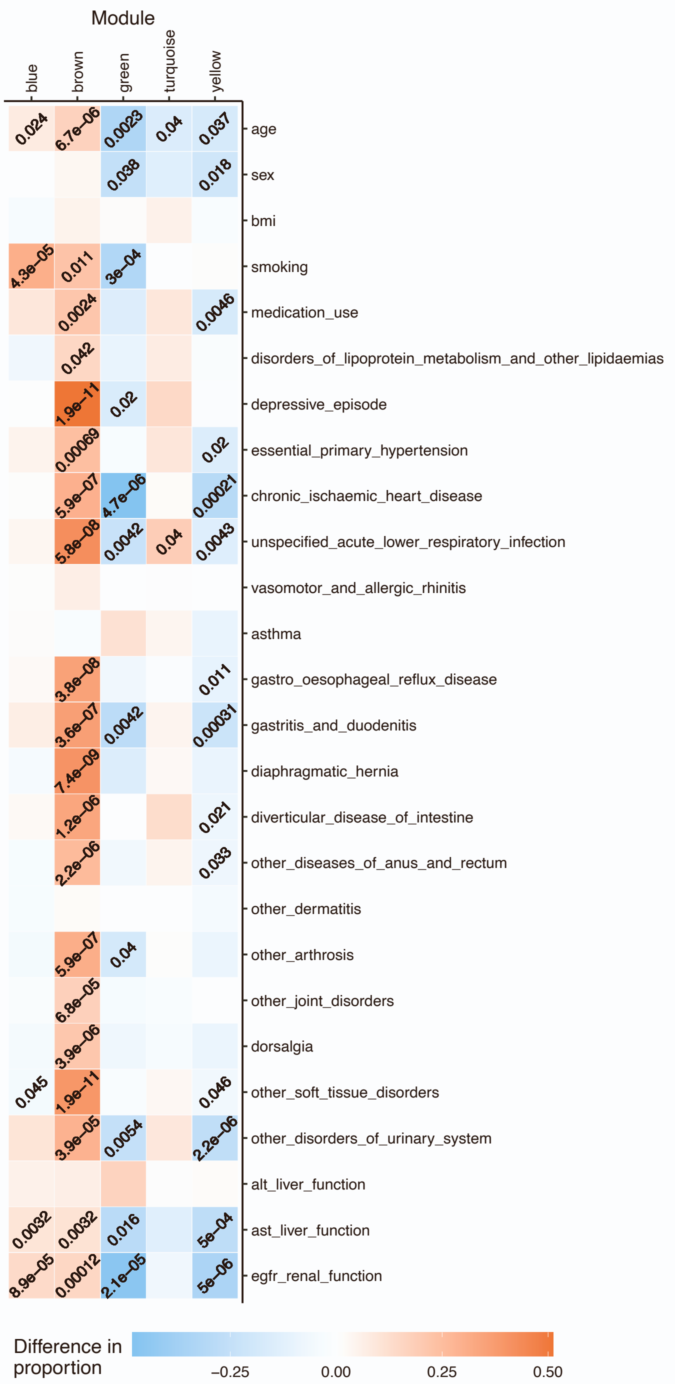

Fig S13: Enrichment (or depletion) of proteins with significant protein-trait associations in the UK Biobank plasma study [27], related to fig 4. Each Weighted Gene Network Correlation Analysis (WGCNA) module was compared to the remaining modules using the Fisher exact test on the numbers of proteins that are significant or not for each trait (colours represent the difference in significant proportion, blue being lower and red higher). Comparisons with False Discovery Rate (FDR) values < 0.05 are displayed in the relevant cells. *P*-values, FDR-values and group sizes can be found in table S10.

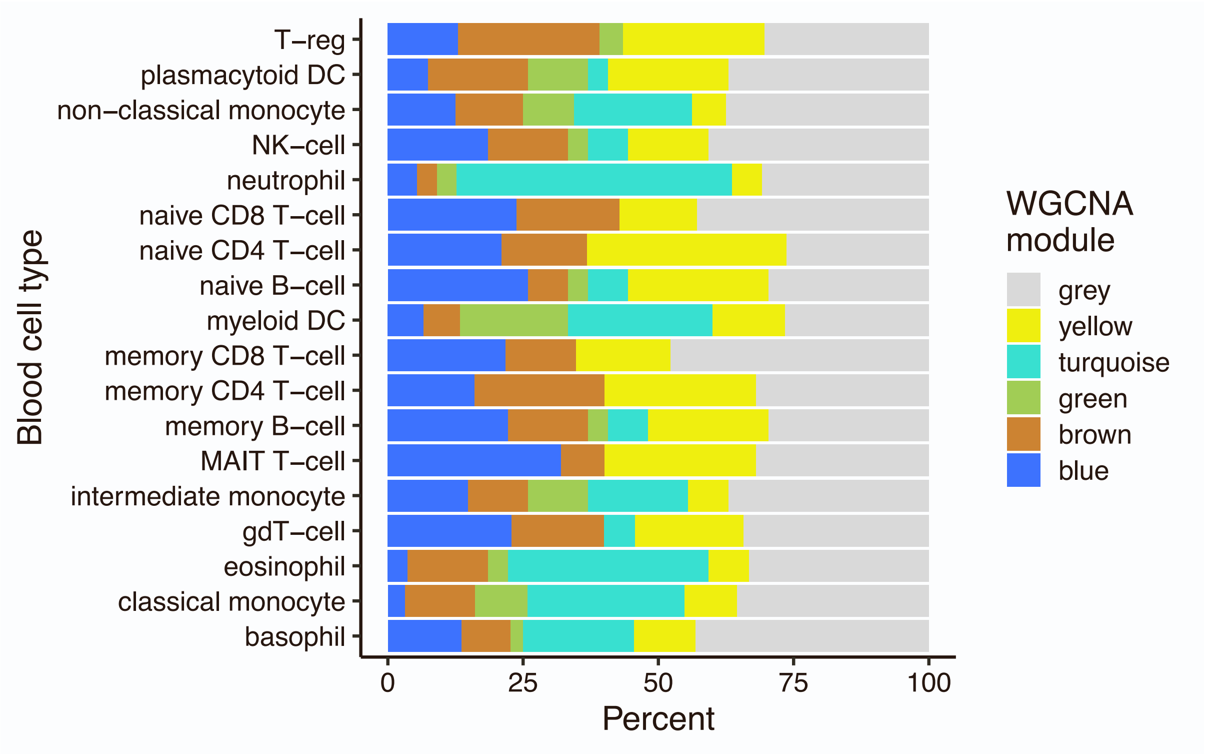

Fig S14: Immune cell enrichment of Weighted Gene Correlation Network Analysis (WGCNA) modules, related to Fig 4. Annotations from version 24 of the Human Protein Atlas [S1] are used to display how many percent of immune cell-enriched proteins that are assigned to the different WGCNA modules (coloured by their names, i.e. blue, brown, green, turquoise, yellow, and grey).

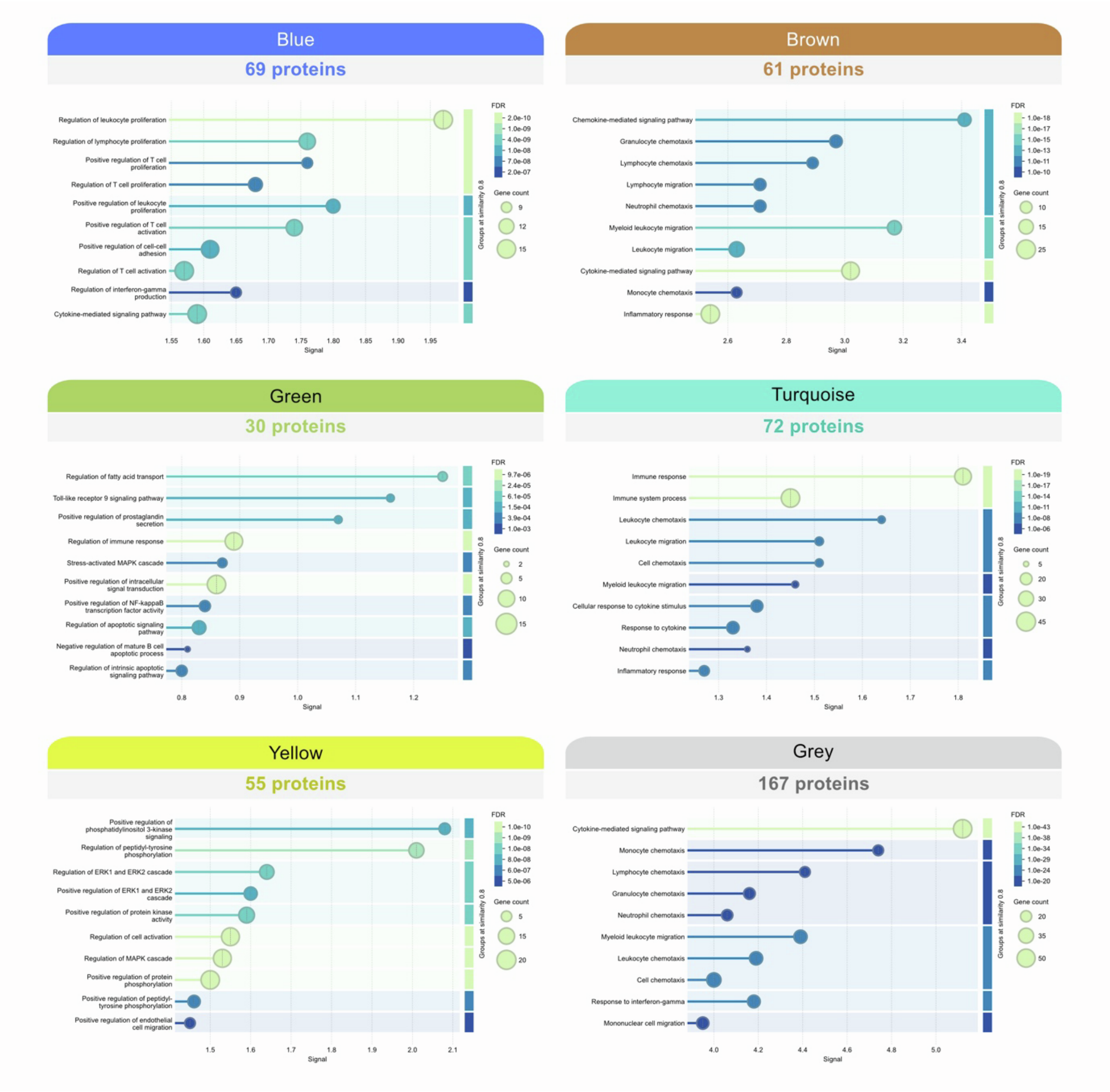

Fig S15: Enrichment of Gene Ontology Biological Process (GO-BP) terms in modules of Weighted Gene Correlation Network Analysis (WGCNA), related to Fig 4. The proteins included in each module were investigated using the STRING database (version 12.0). The plots show the 10 terms with the highest signal from GO-BP. A lighter colour represents a lower false discovery rate (FDR), while the circle sizes represent gene counts. The static links for the query results are provided below:
Blue: https://version-12-0.string-db.org/cgi/network?networkId=bUkLWxExr4Uh;
Brown: https://version-12-0.string-db.org/cgi/network?networkId=bvtY986SSH4t;
Green: https://version-12-0.string-db.org/cgi/network?networkId=btdudWJo2cCf;
Turquoise: https://version-12-0.string-db.org/cgi/network?networkId=bxtMO881SM1S;
Yellow: https://version-12-0.string-db.org/cgi/network?networkId=bVq2IBDglYBF;
Grey: https://version-12-0.string-db.org/cgi/network?networkId=bBkyAqFexLbe.

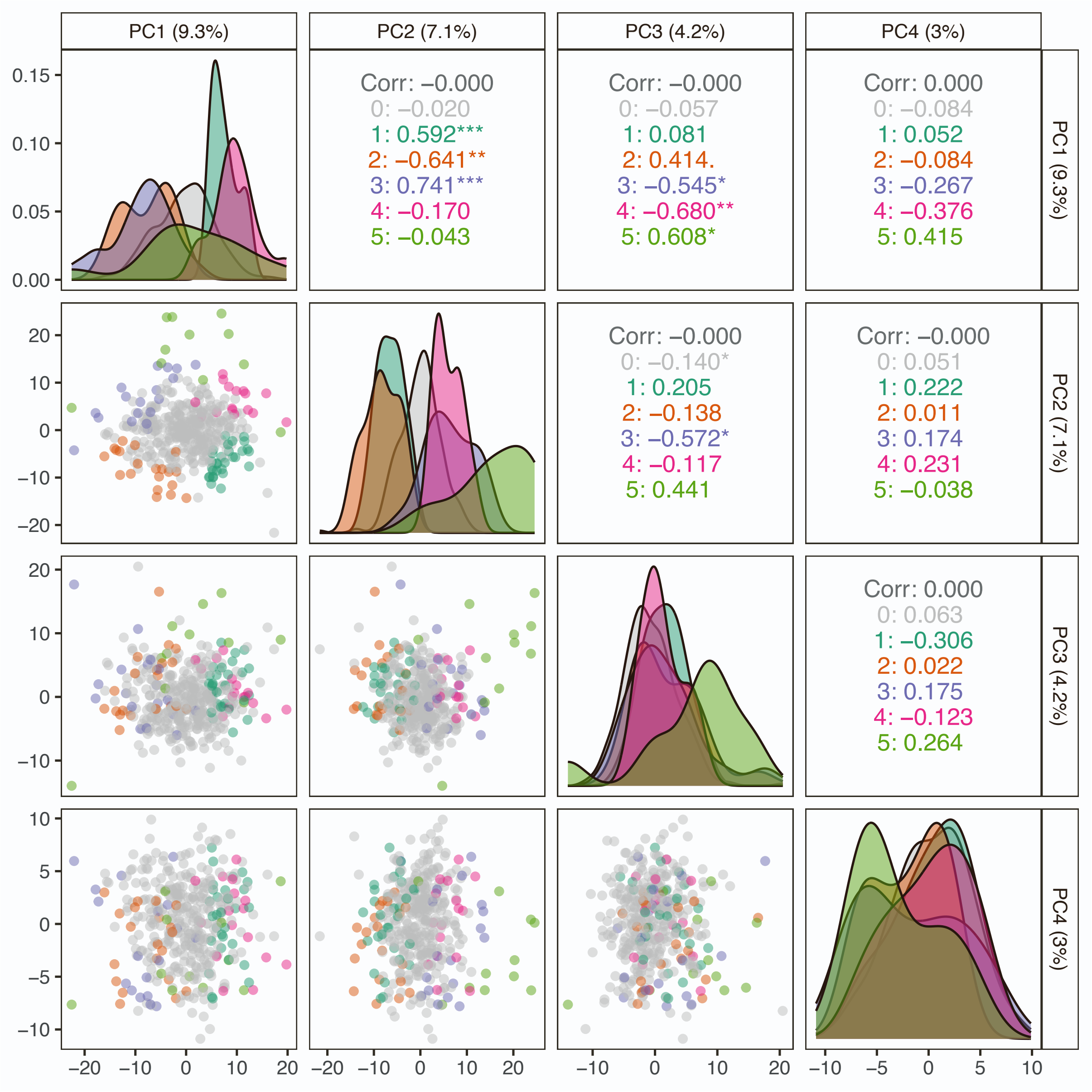

Fig S16: The first four principal components (PCs) of the proteomics data, coloured by proteotype, related to Fig 4. The diagonal shows the distributions of each proteotype along each PC (grey: proteotype 0; dark green: 1; orange: 2; blue: 3; pink: 4; light green: 5). Plots above the diagonal show the Pearson correlation of two PCs as well as the correlation of the two PCs for samples in a proteotype, while the plots below the diagonal show scatter plots of the two PCs.

Supplementary tables S1-S10 (separate file)

Table S1. Frequencies of autoreactive anti-IFN antibodies per serocluster, related to Table 2.

Table S2. Variable importance from Lasso regression using antibody levels to predict different traits, related to Fig S1.

Table S3. Protein correlation and detectability in different platforms, related to Fig S8.

Table S4. Protein-antibody associations (linear (logistic) regression), related to Fig S9.

Table S5. Protein-serocluster associations (Kruskal-Wallis test), related to Fig S10.

Table S6. Protein-serocluster associations (Pairwise Wilcoxon rank-sum test), related to Fig S10.

Table S7. Protein-questionnaire associations in serocluster 1 (the seronegative population) (linear regression), related to Fig S11.

Table S8. Variable importance from Lasso regression using protein levels to predict different traits, related to Fig 3.

Table S9. Protein WGCNA module memberships and their contributions to the module eigengenes, related to Fig 4.

Table S10. WGCNA module associations with UK Biobank (UKB) traits (Fisher exact test), related to Fig S13.

Supplementary references

[S1] The Human Protein Atlas, version 24. https://v24.proteinatlas.org/.
