# Supplementary material for: Exploration of immune phenotypes in self-sampling citizens
Source: iScience. 2026 Jan 3;29(2):114611. doi: 10.1016/j.isci.2025.114611 (PMC12860695; doi:10.1016/j.isci.2025.114611)
Supplement: Data S1. The questions sent out together with sampling devices for health information, related to STAR Methods [file mmc2.pdf]

| Corresponding variable in main text | Question | Possible responses | Comment |
|---|---|---|---|
| Age group | 1. To which age group do you belong? | 18-29<br>30-39<br>40-49<br>50-59<br>60-69 | |
| Sex | 2. What is your sex? | Female<br>Male<br>Other | |
| Infection | 3a. Have you been diagnosed with COVID-19? | Yes<br>No | Counted as infected if any of 3a, 4a, and 5a is Yes |
| | 3b. If yes, when? | January-March 2020<br>April-June 2020<br>July-September 2020<br>October-December 2020<br>Janaury-March 2021<br>April-June 2021 | Only answered if 3a is Yes |
| | 3c. If yes, have you received care for COVID-19 at a hospital? | Yes<br>No | Only answered if 3a is Yes |
| Infection | 4a. Have you tested positive against COVID-19 with a PCR test? | Yes<br>No | Counted as infected if any of 3a, 4a, and 5a is Yes |
| | 4b. If yes, when? | January-March 2020<br>April-June 2020<br>July-September 2020<br>October-December 2020<br>Janaury-March 2021<br>April-June 2021 | Only answered if 4a is Yes |
| Infection | 5a. Have you tested positive for antibodies against the corona virus? | Yes<br>No | Counted as infected if any of 3a, 4a, and 5a is Yes |
| | 5b. If yes, when? | January-March 2020<br>April-June 2020<br>July-September 2020<br>October-December 2020<br>Janaury-March 2021<br>April-June 2021 | Only answered if 5a is Yes |
| Symptoms (loss of) | 6. Have you experienced loss of sense of smell or taste during the last year? | Yes, smell<br>Yes, taste<br>No | Can select yes for both smell and taste |
| Vaccination | 7a. Have you been vaccinated against the corona virus? | Yes<br>No | May be listed as vaccination status (0: no, 1: one dose, 2: two doses) |
| | 7b. If yes, how many doses have you received? | One dose<br>Two doses | Only answered if 7a is Yes |
| | 7c. Which vaccine did you receive? | Pfizer/BioNTech<br>Moderna<br>AstraZeneca<br>Don't know | Only answered if 7a is Yes |
| | 7d. What month did receive your latest dose? | January<br>February<br>March<br>April<br>May | Only answered if 7a is Yes |
