# Supplementary figures and images for: Exploration of immune phenotypes in self-sampling citizens
Source: iScience. 2026 Jan 3;29(2):114611. doi: 10.1016/j.isci.2025.114611 (PMC12860695; doi:10.1016/j.isci.2025.114611)

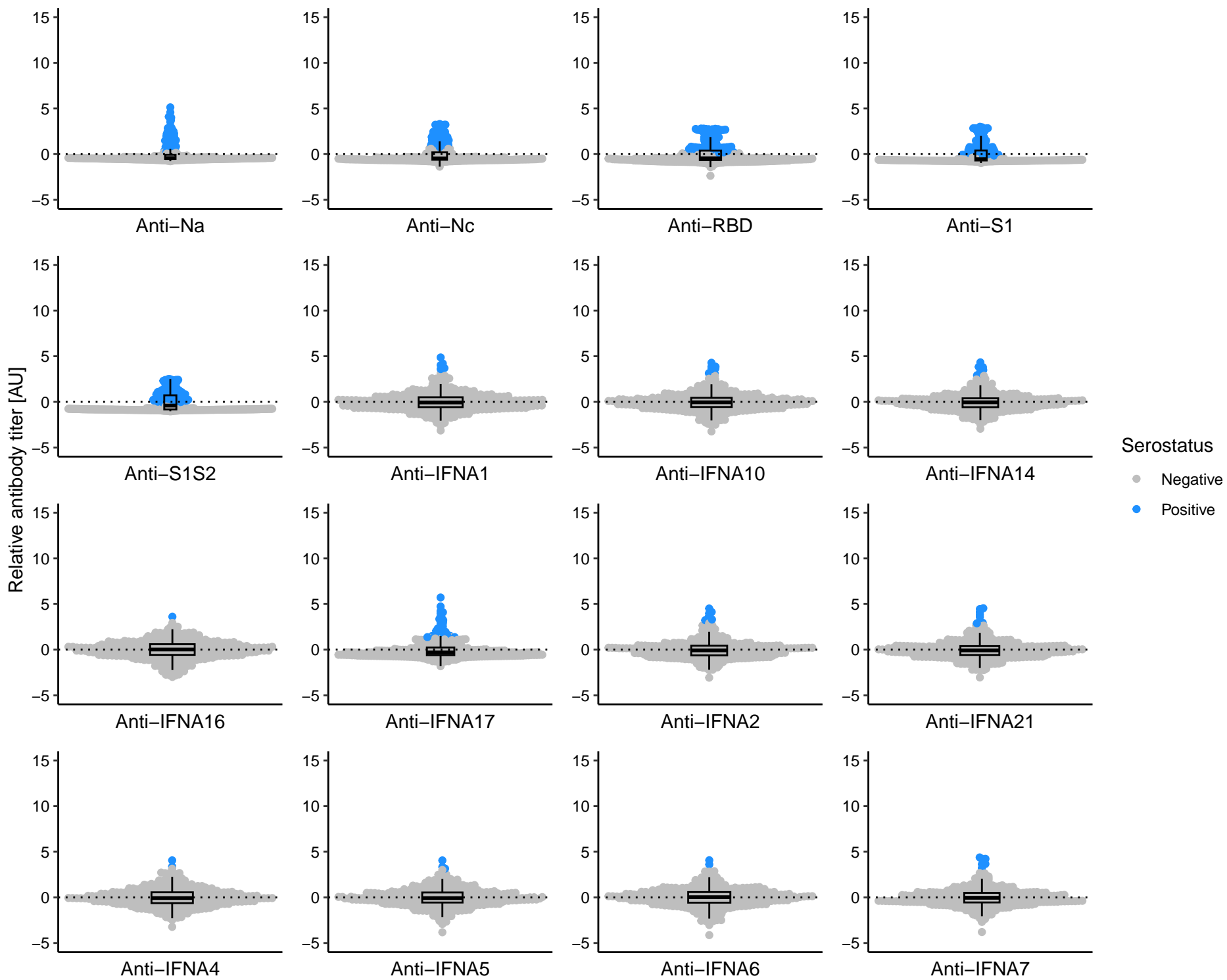

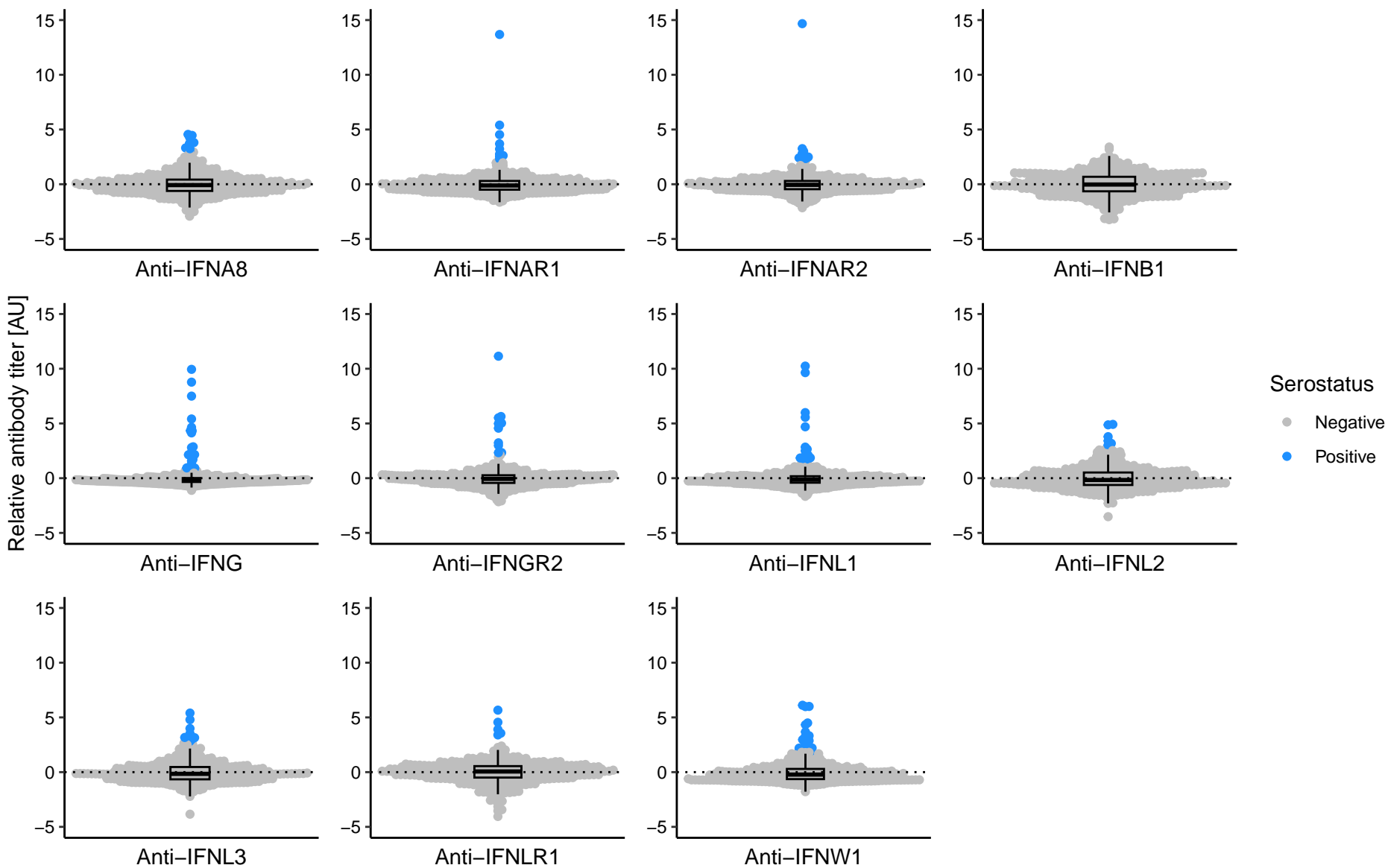

Supplement: Data S2. Boxplots showing the distributions of relative antibody titers from multianalyte serology assays with seropositive samples marked in blue [file mmc3.pdf]

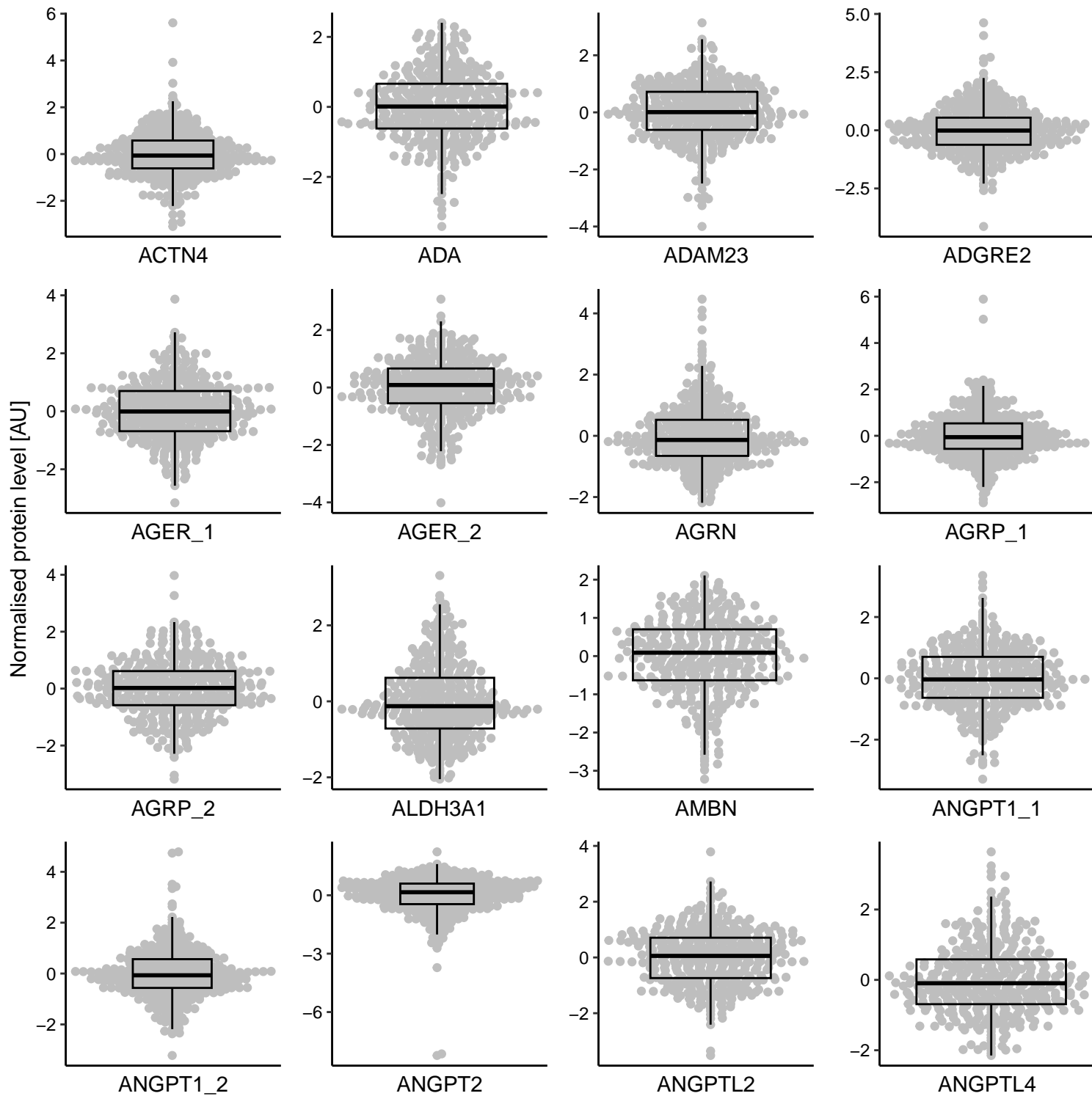

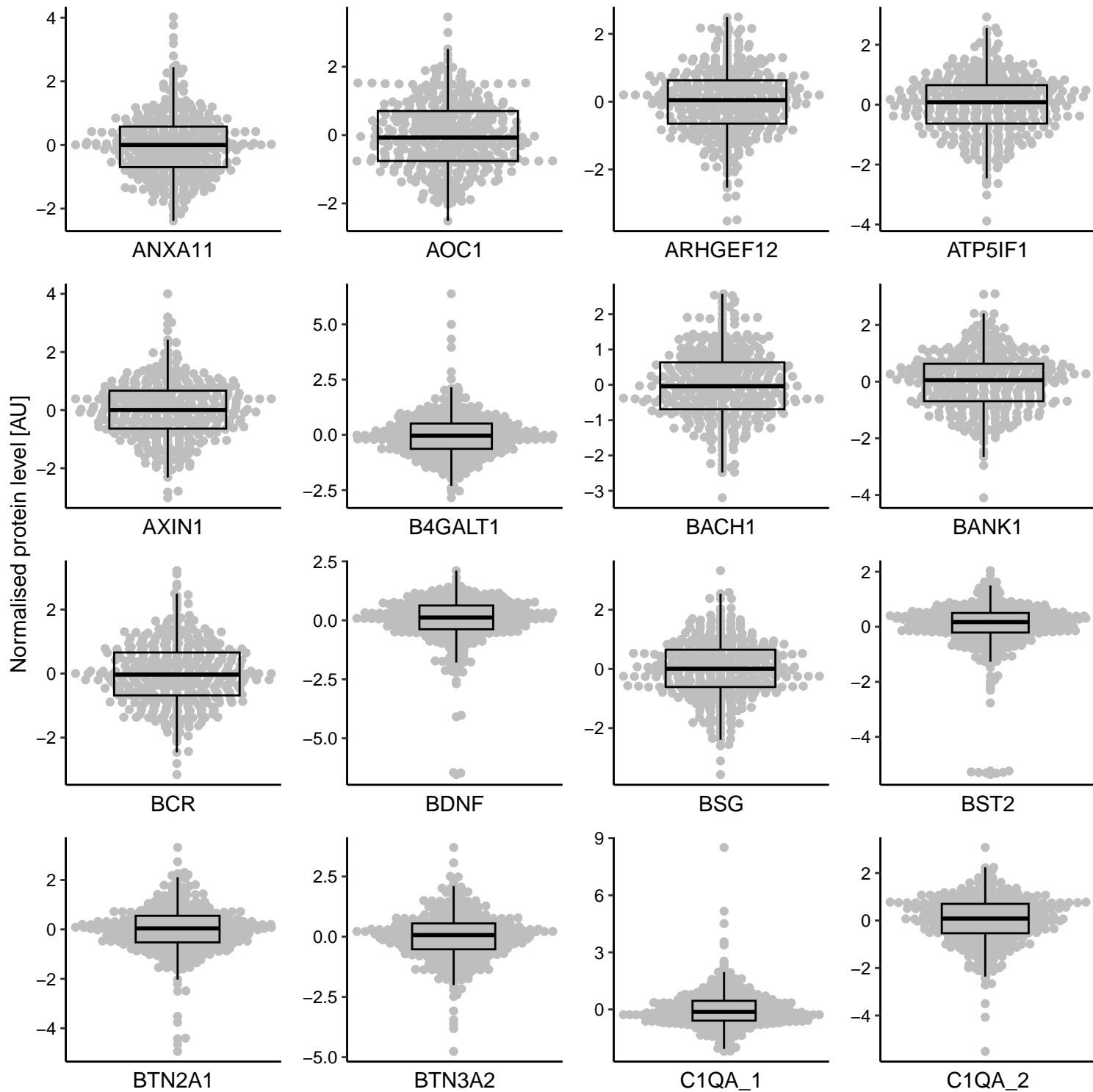

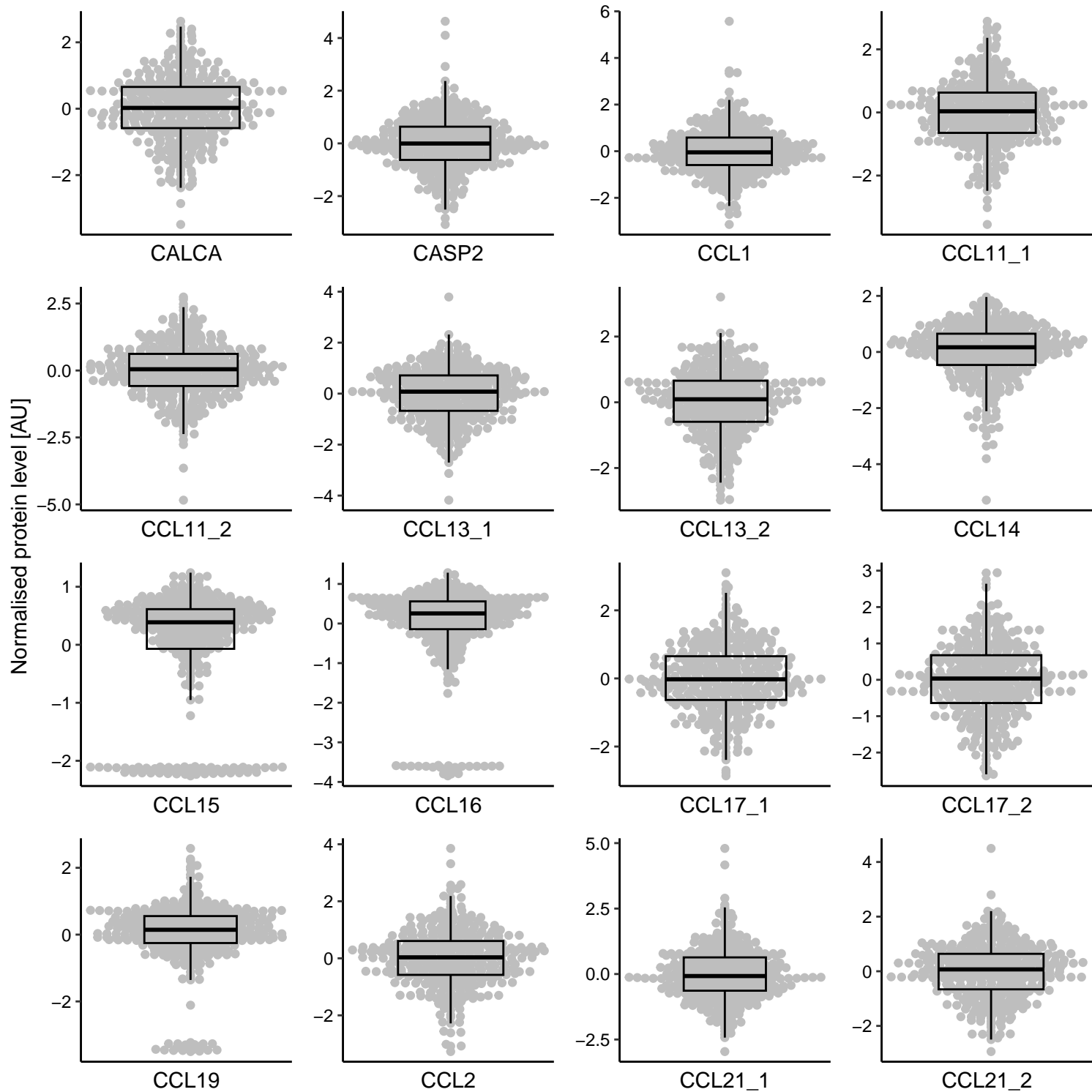

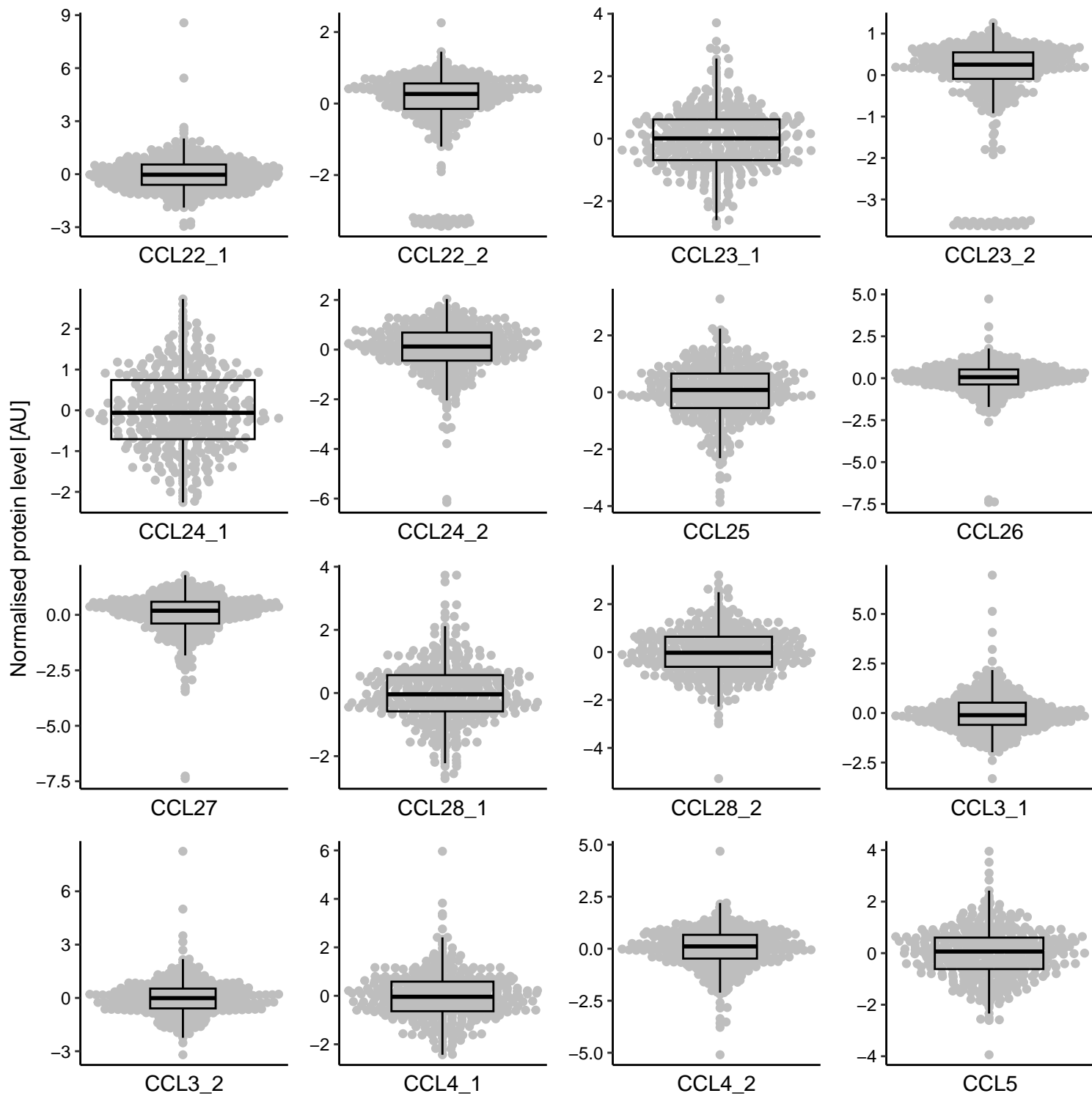

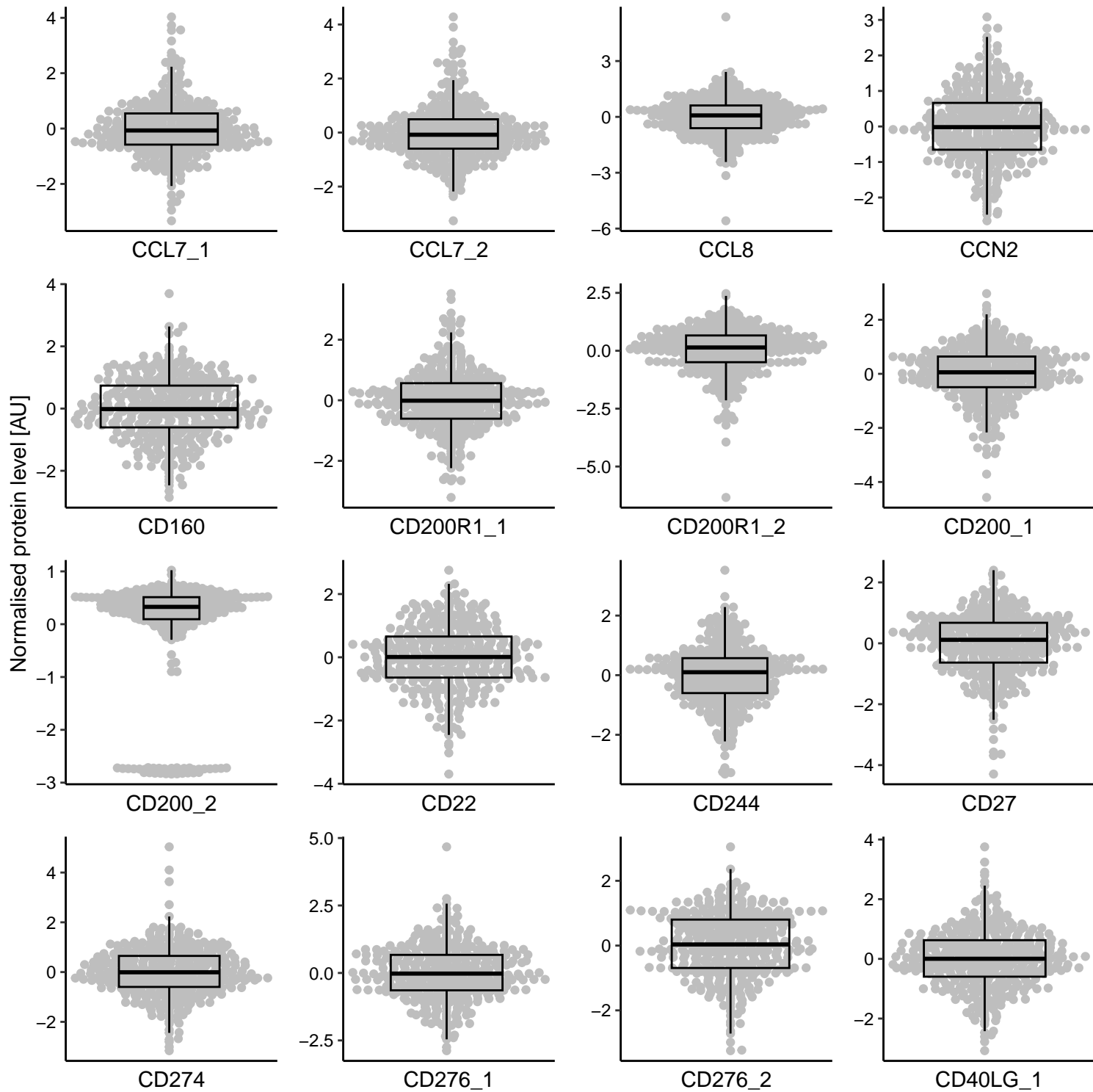

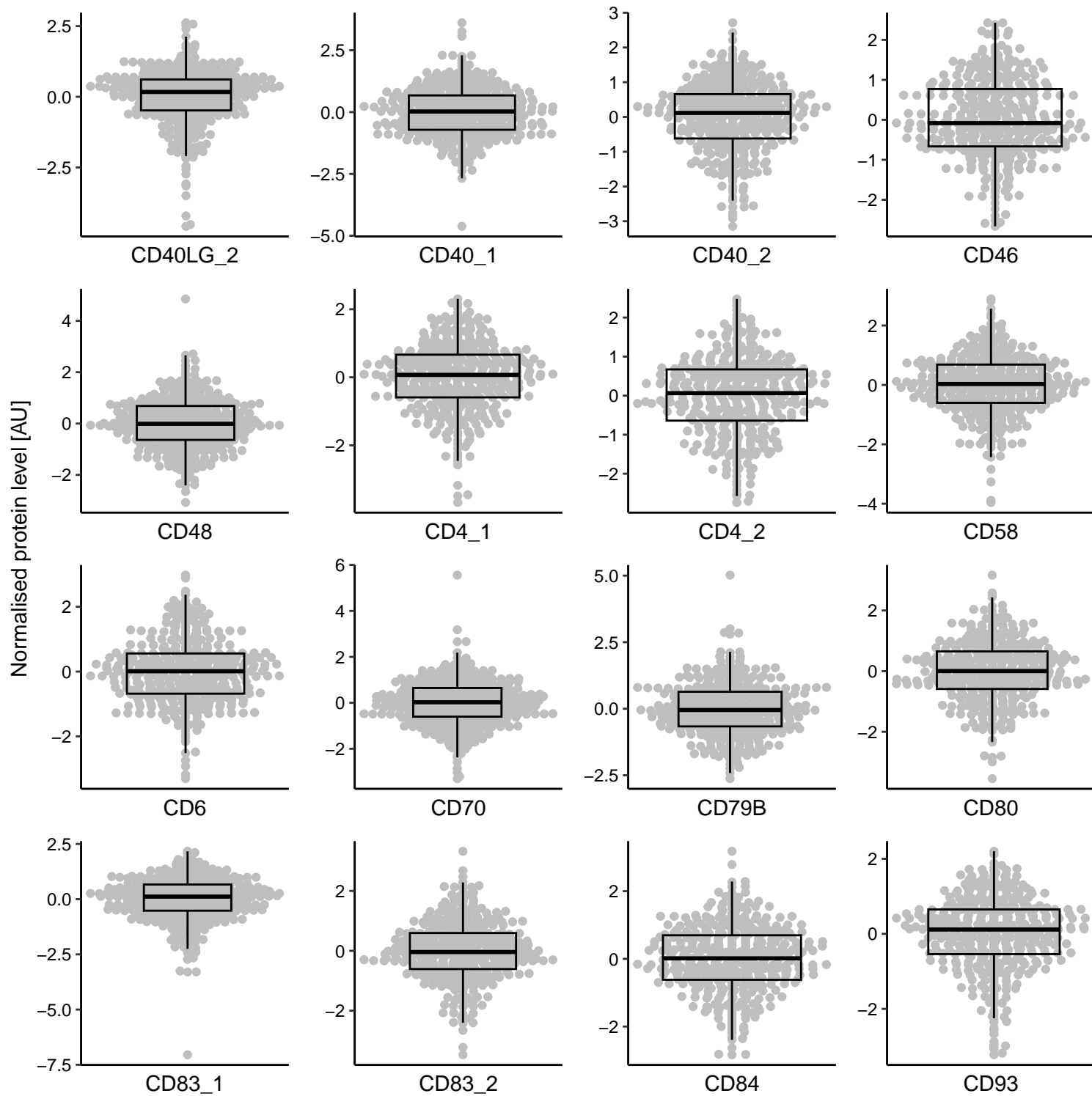

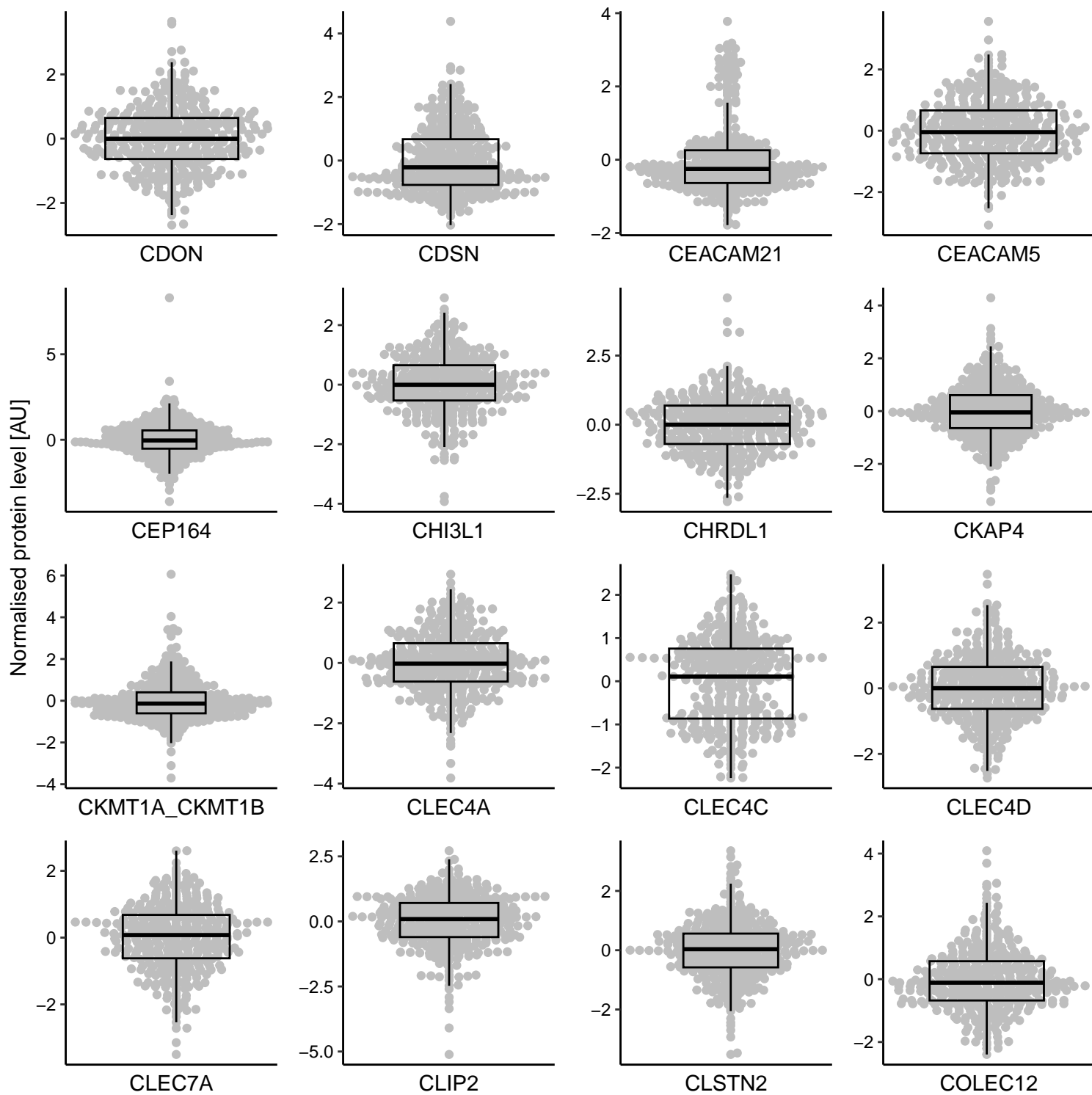

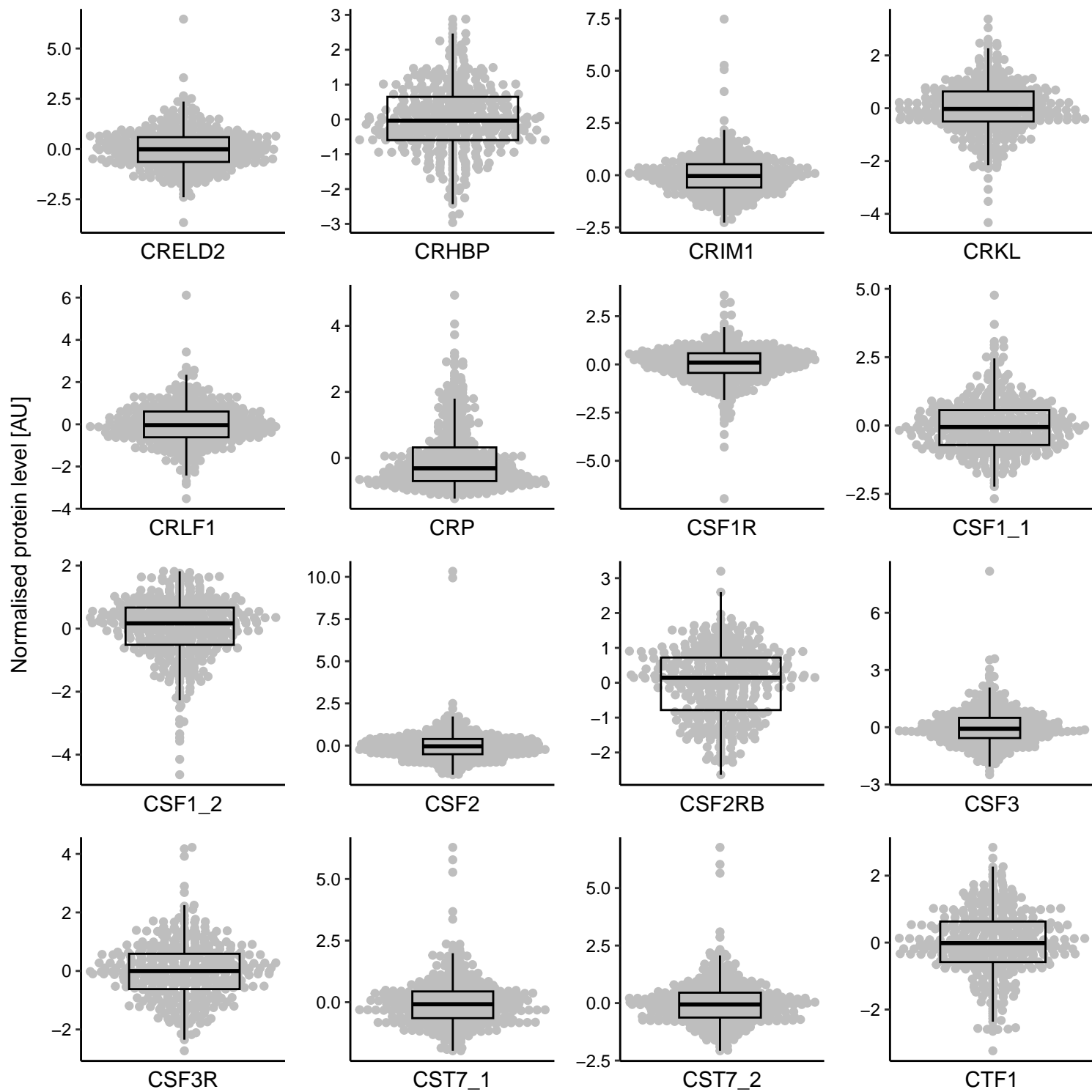

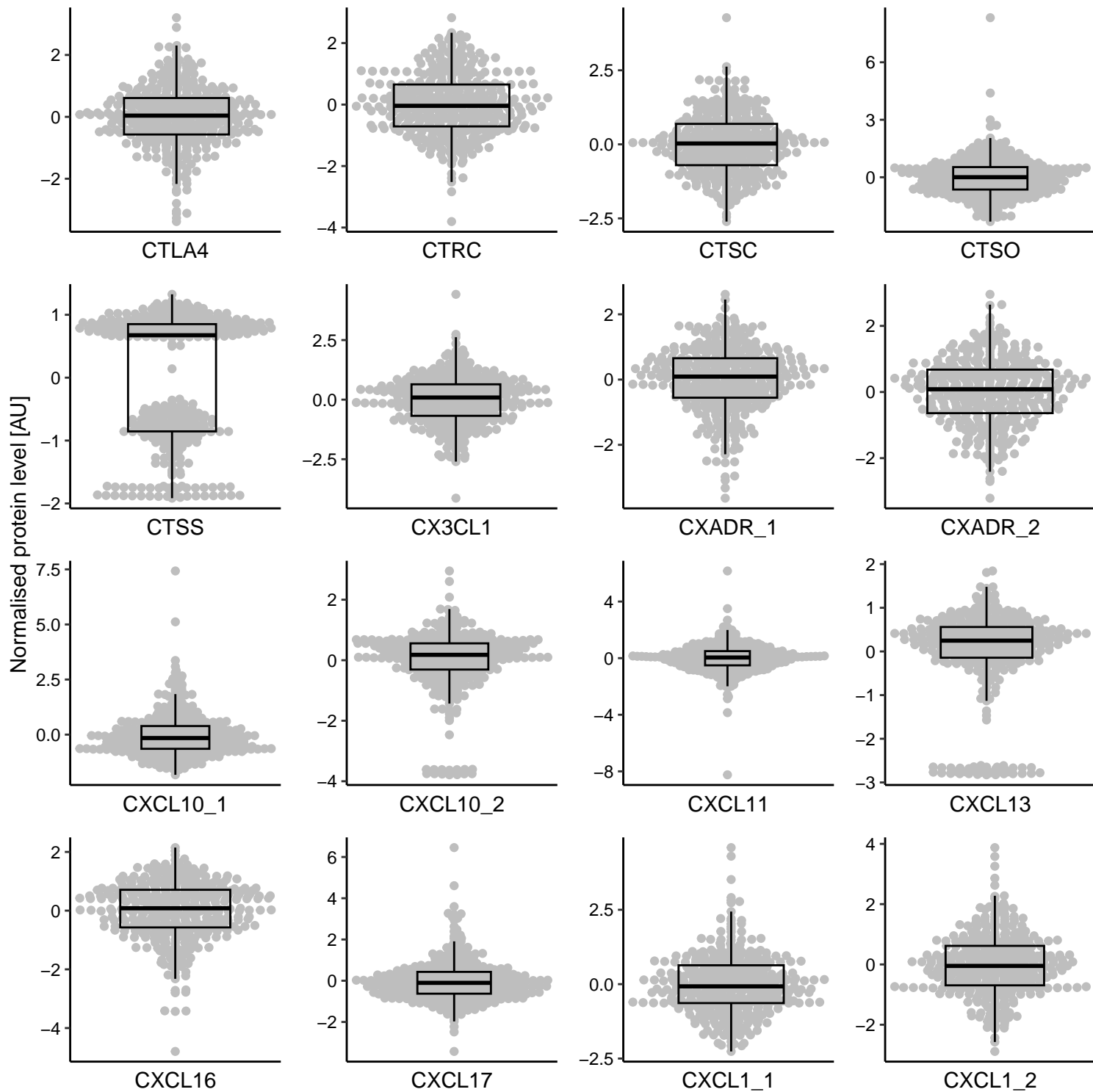

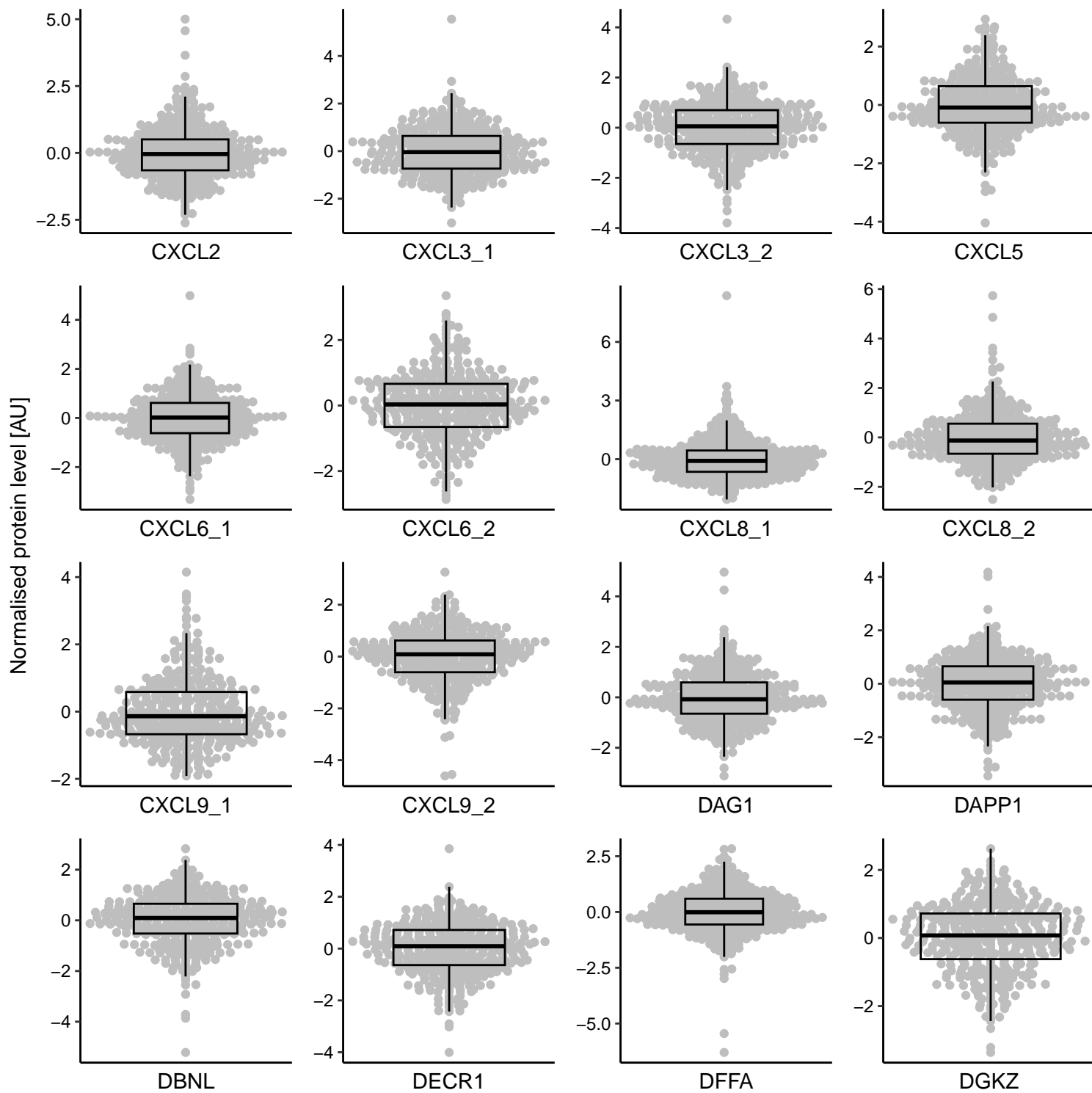

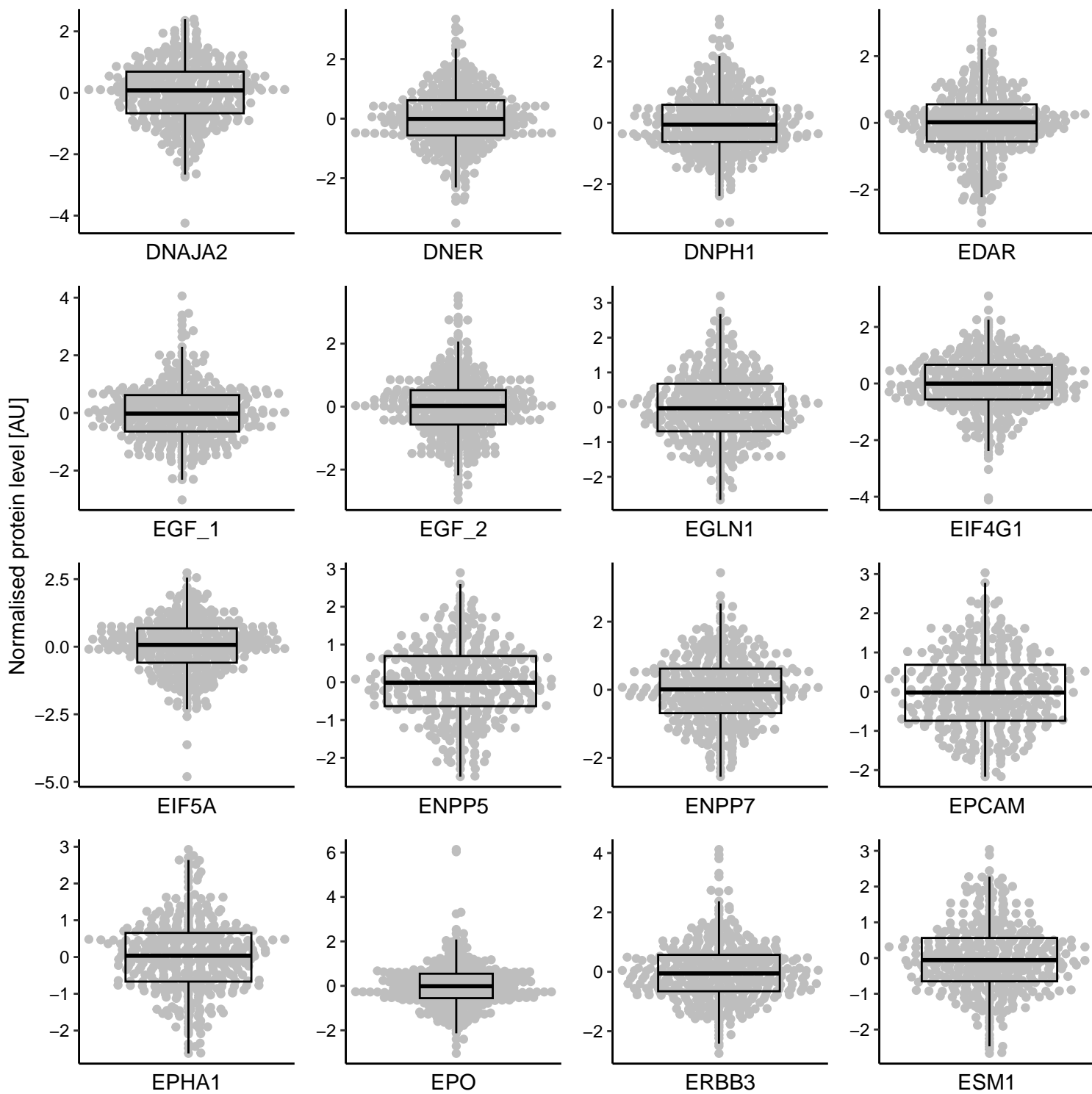

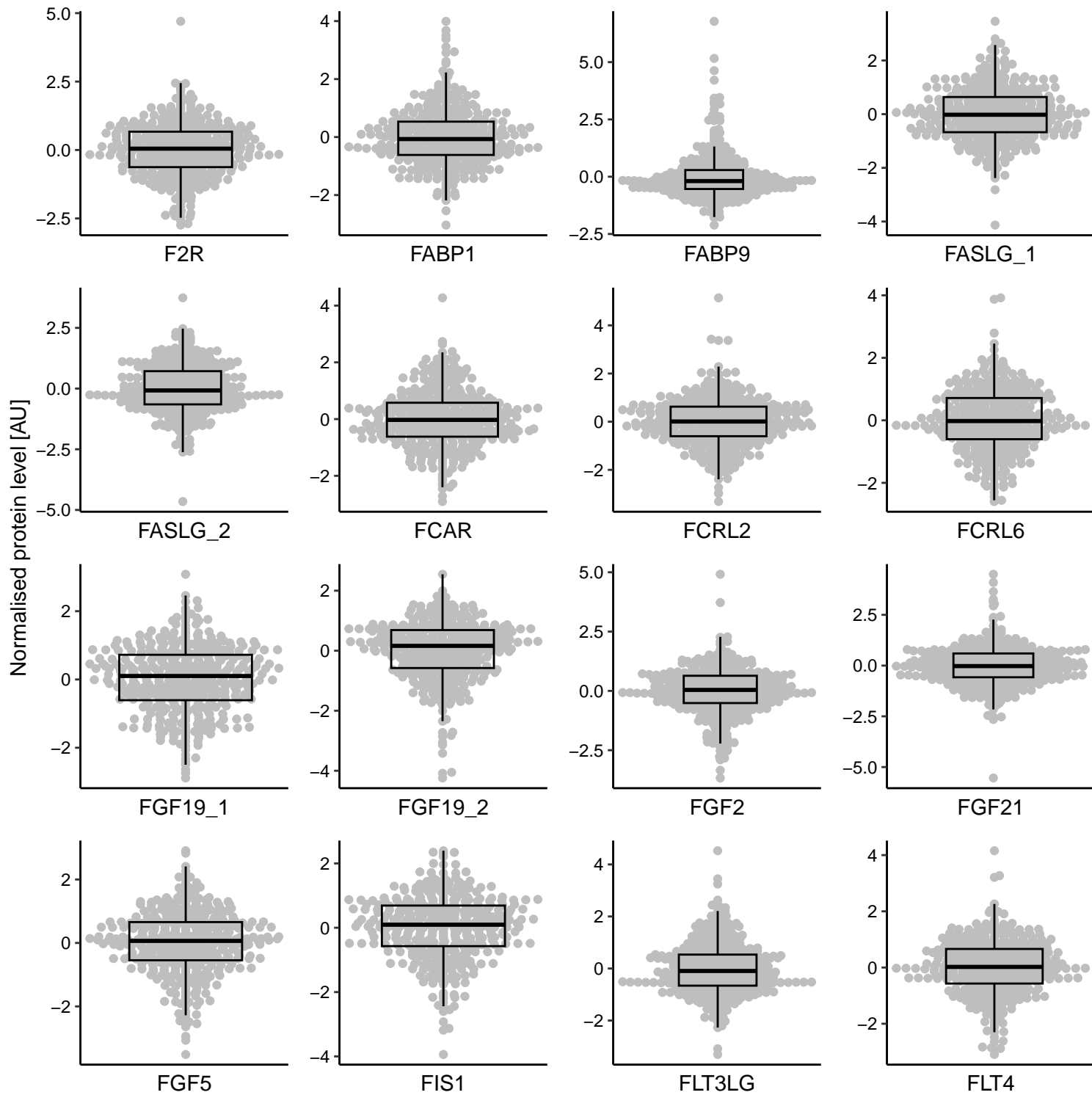

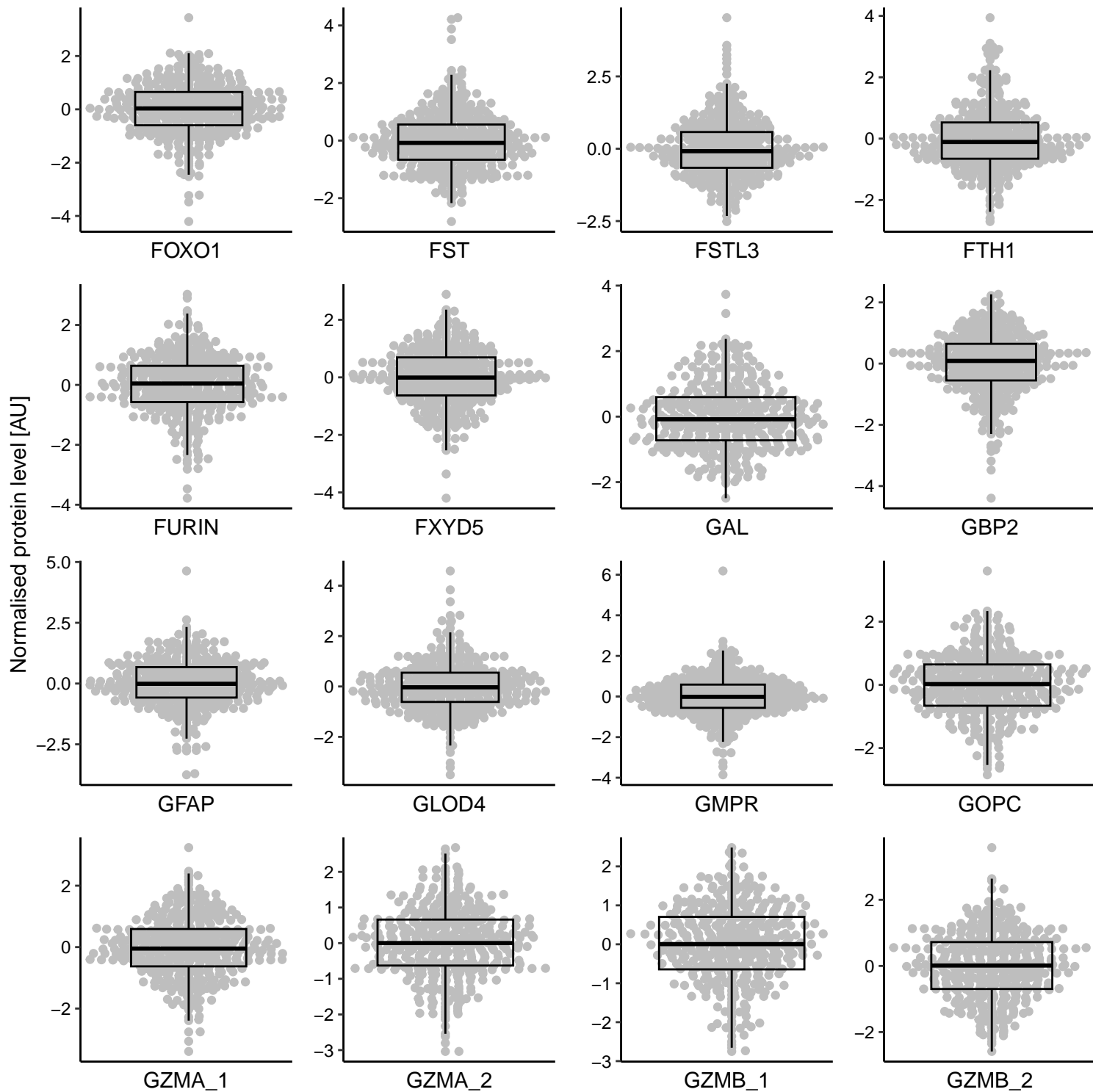

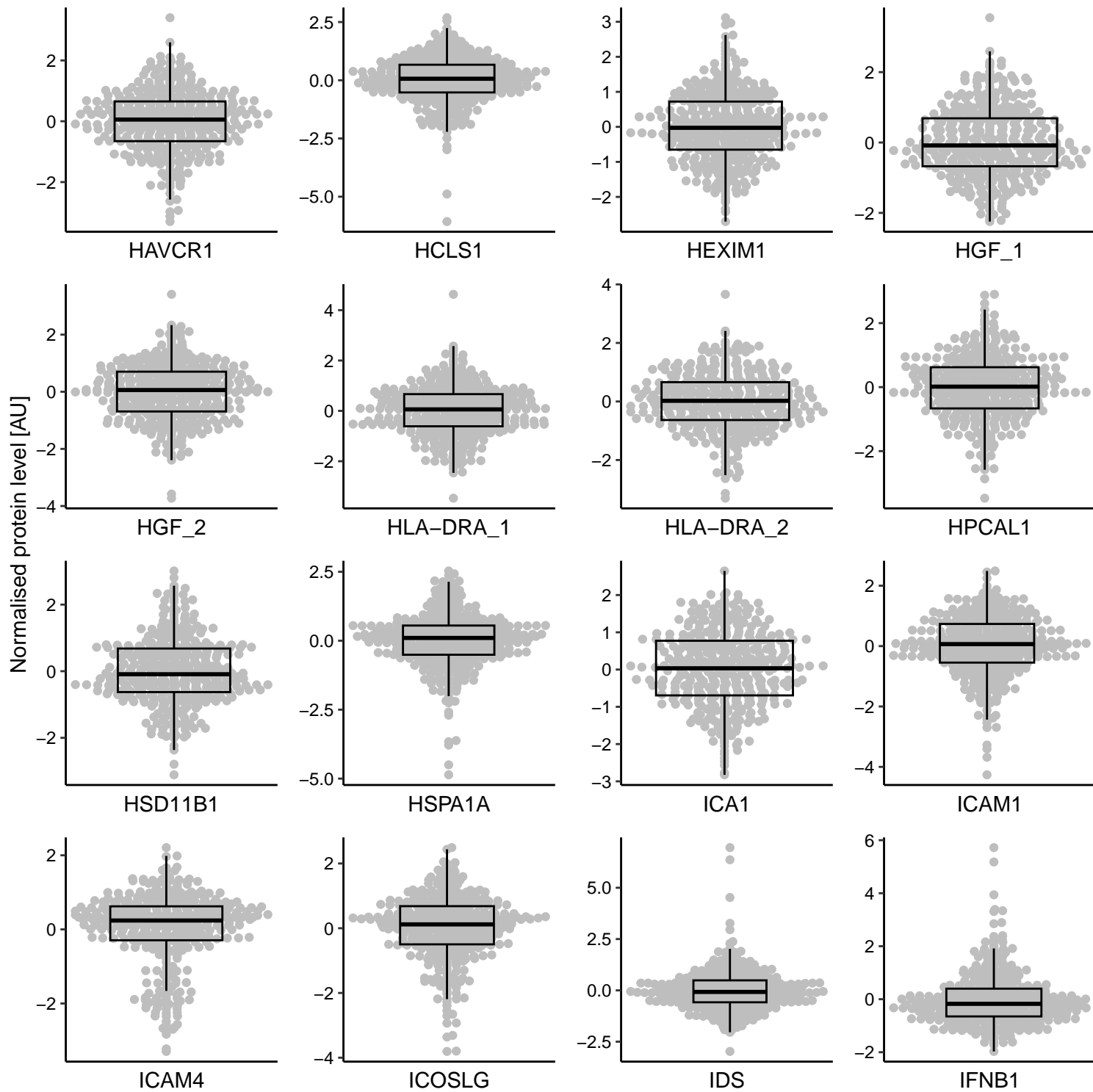

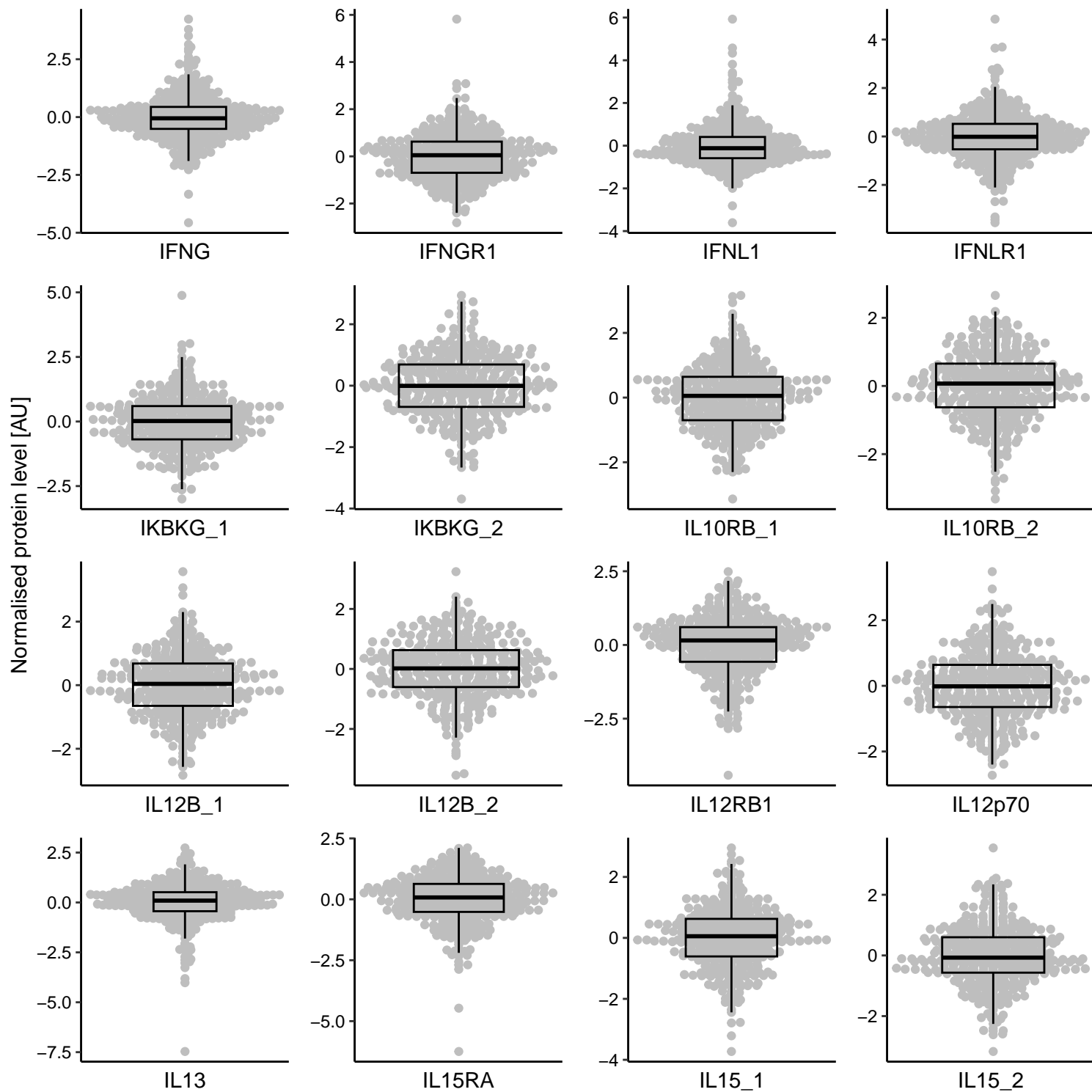

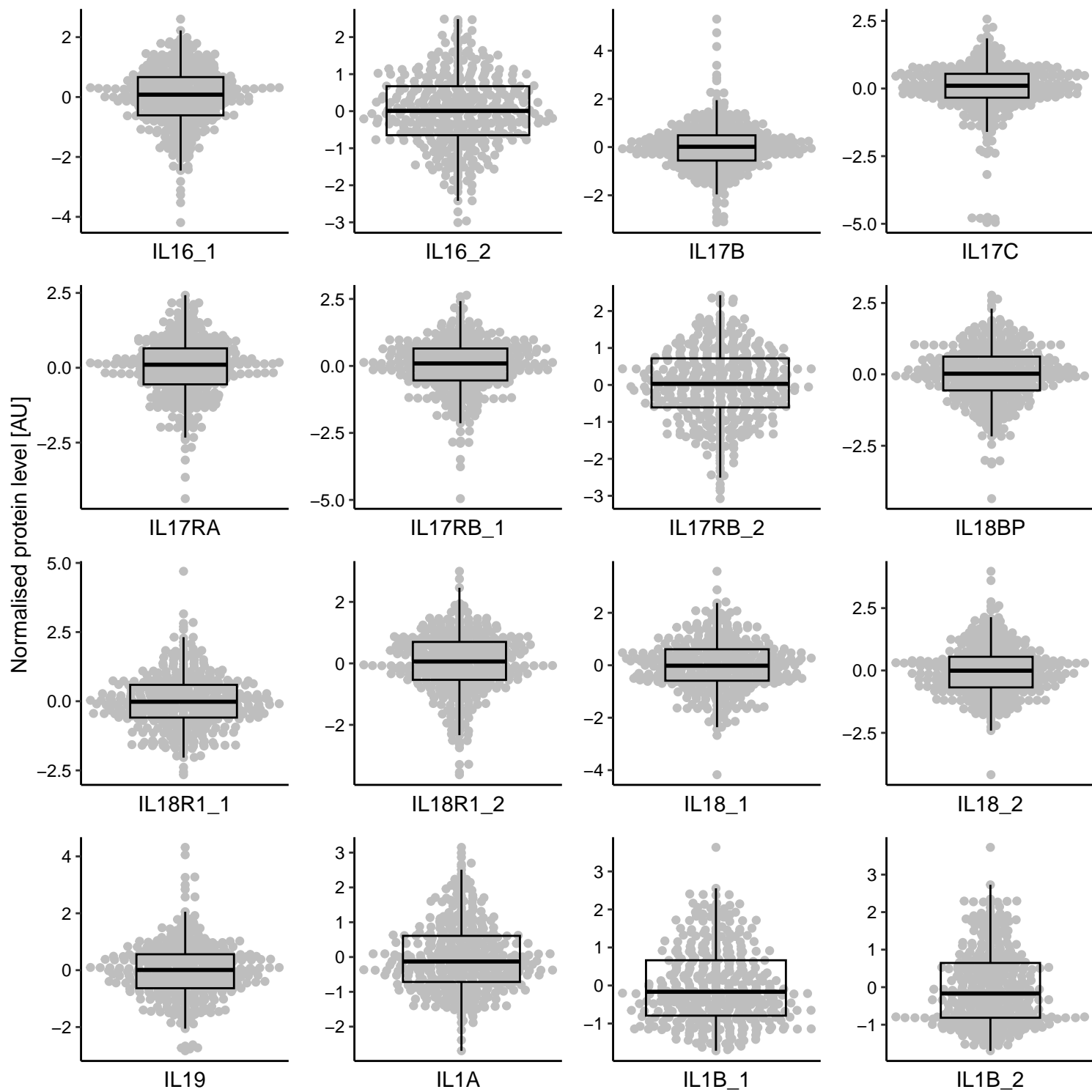

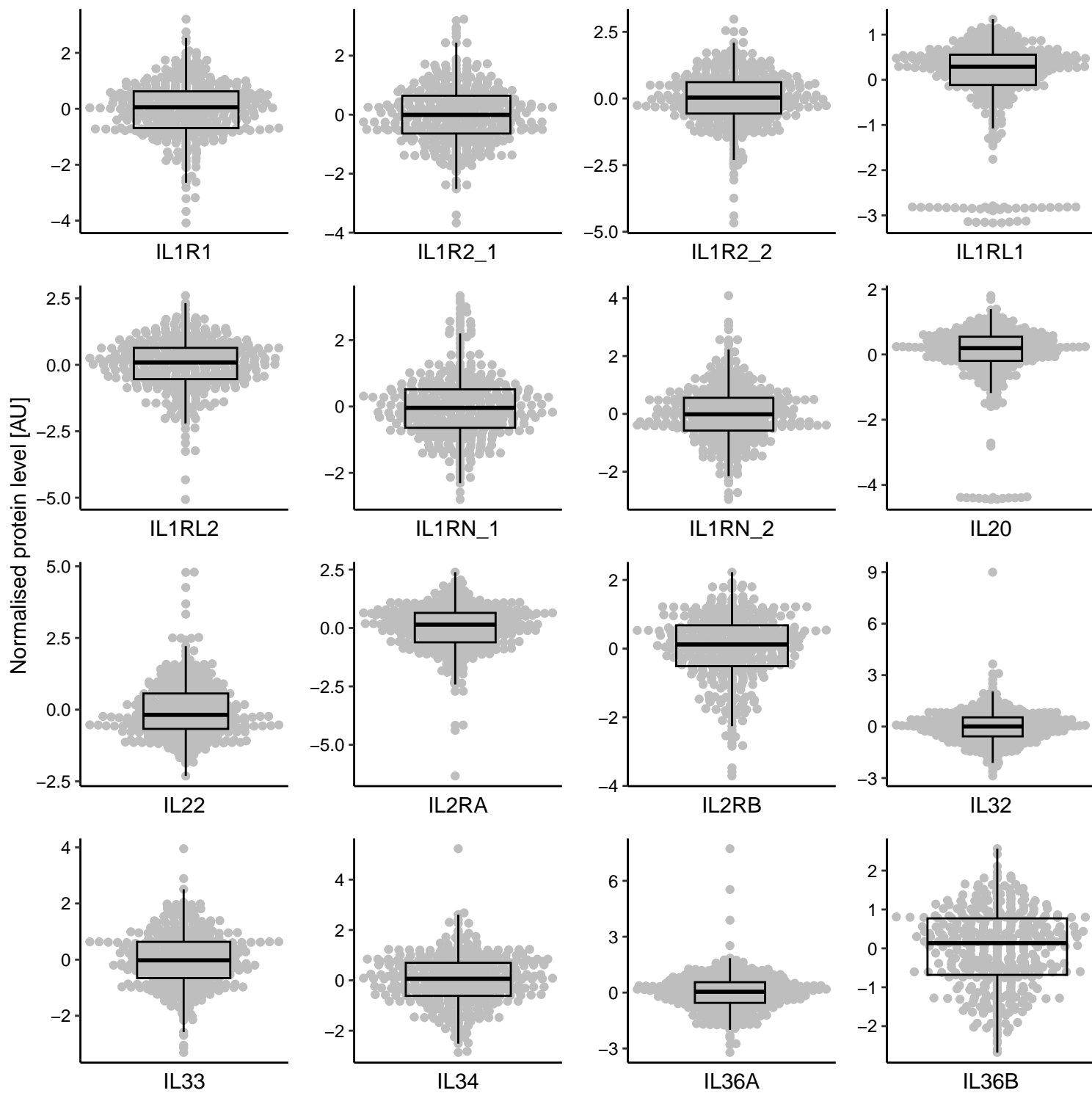

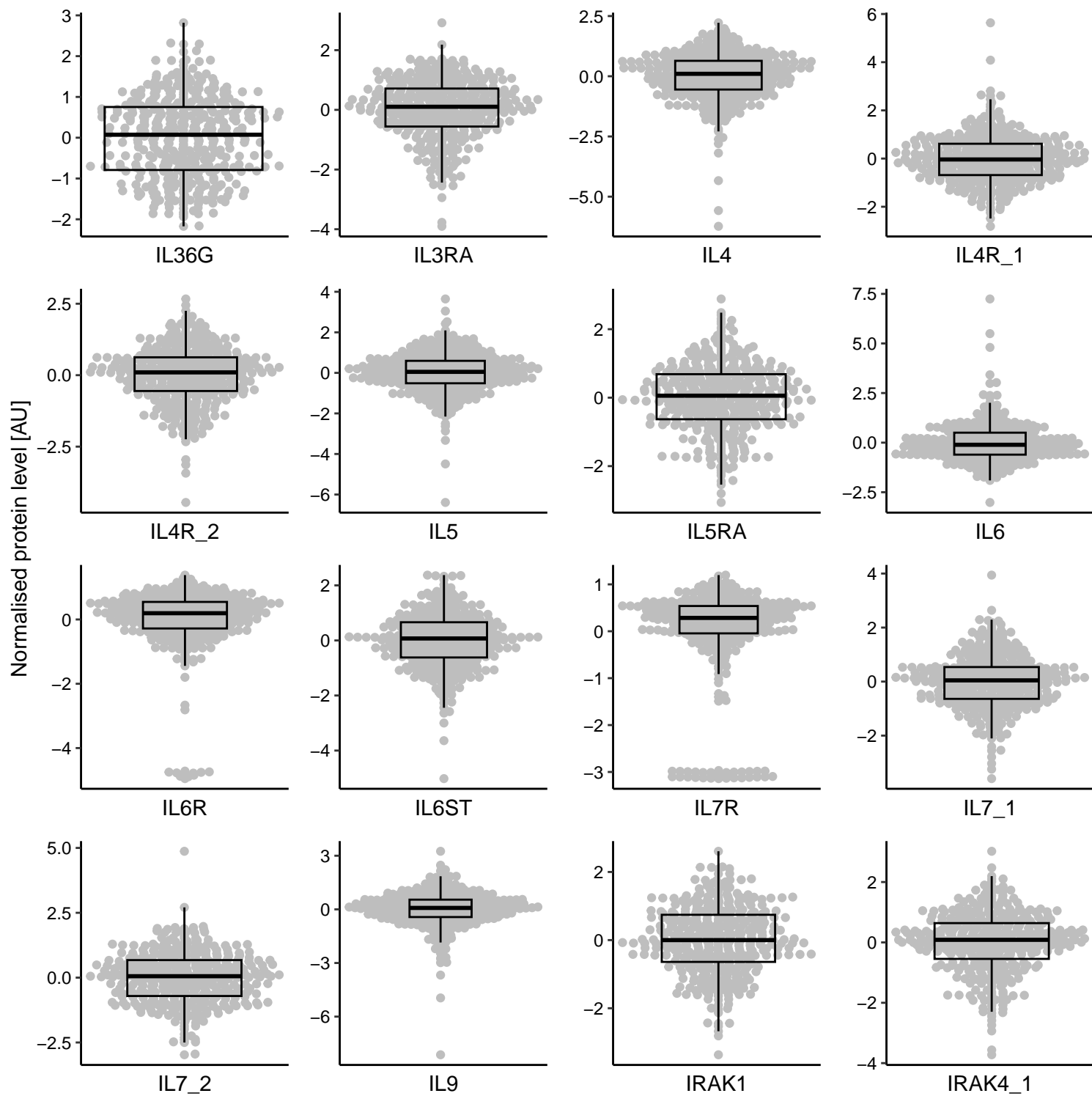

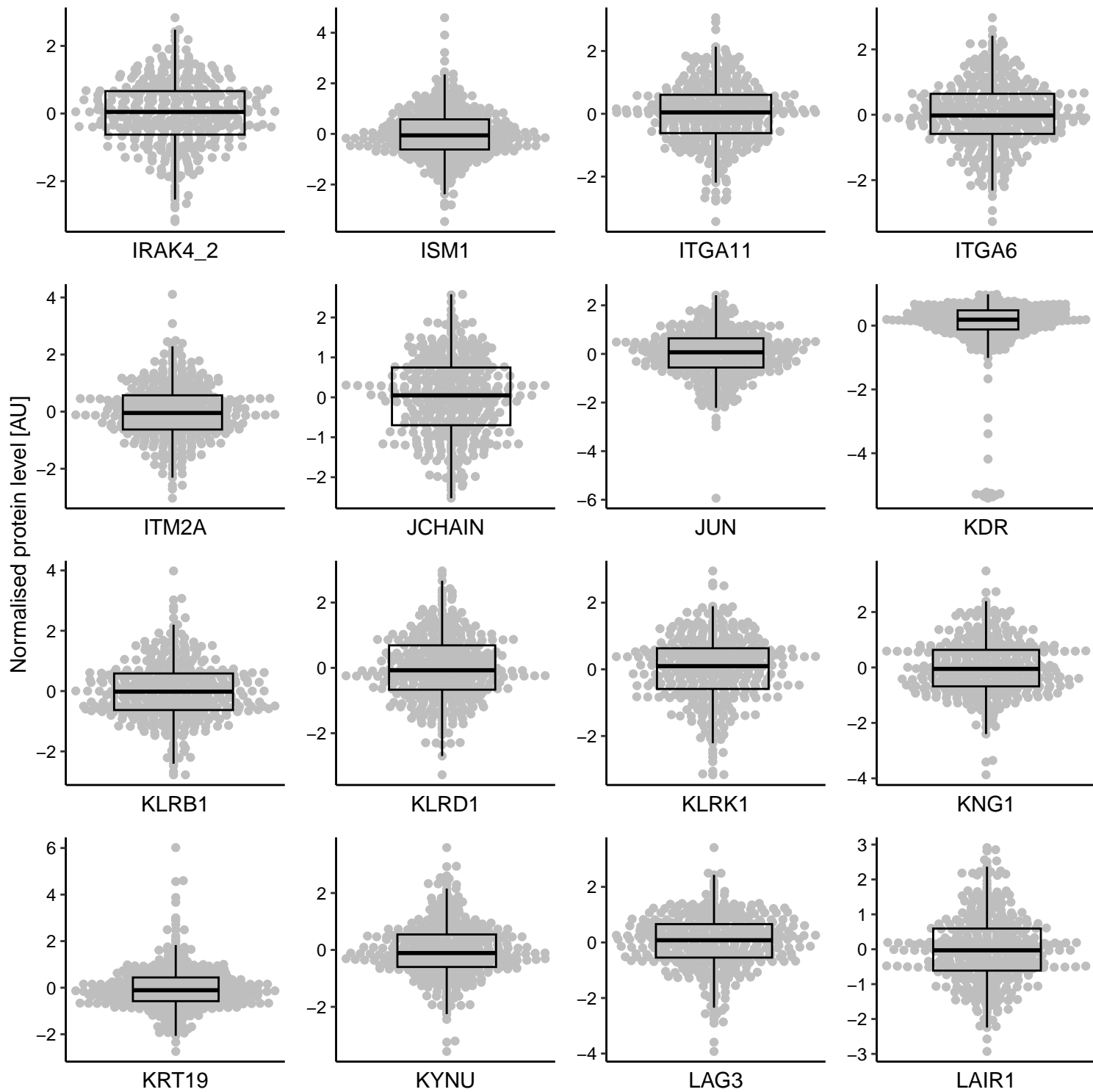

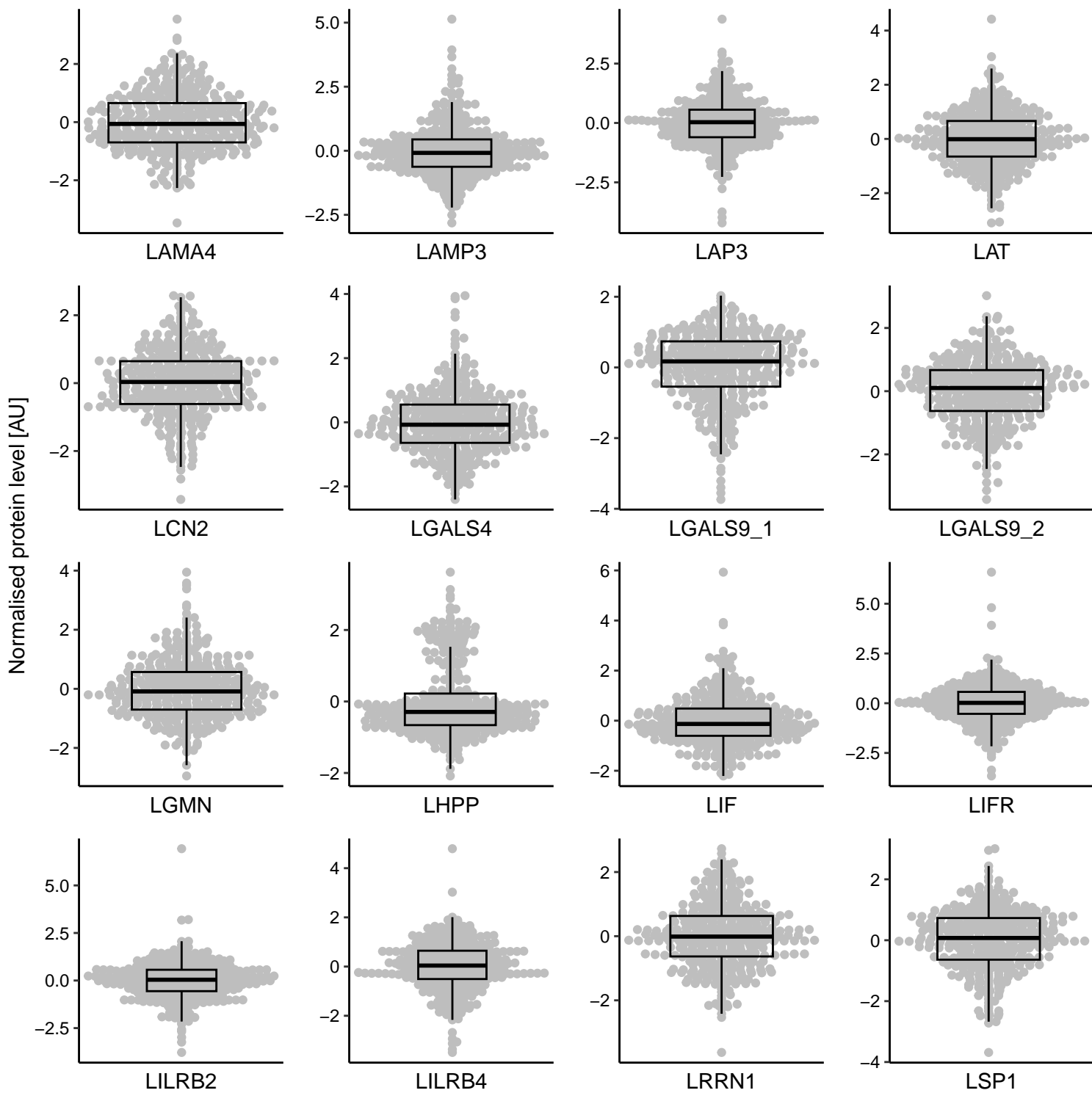

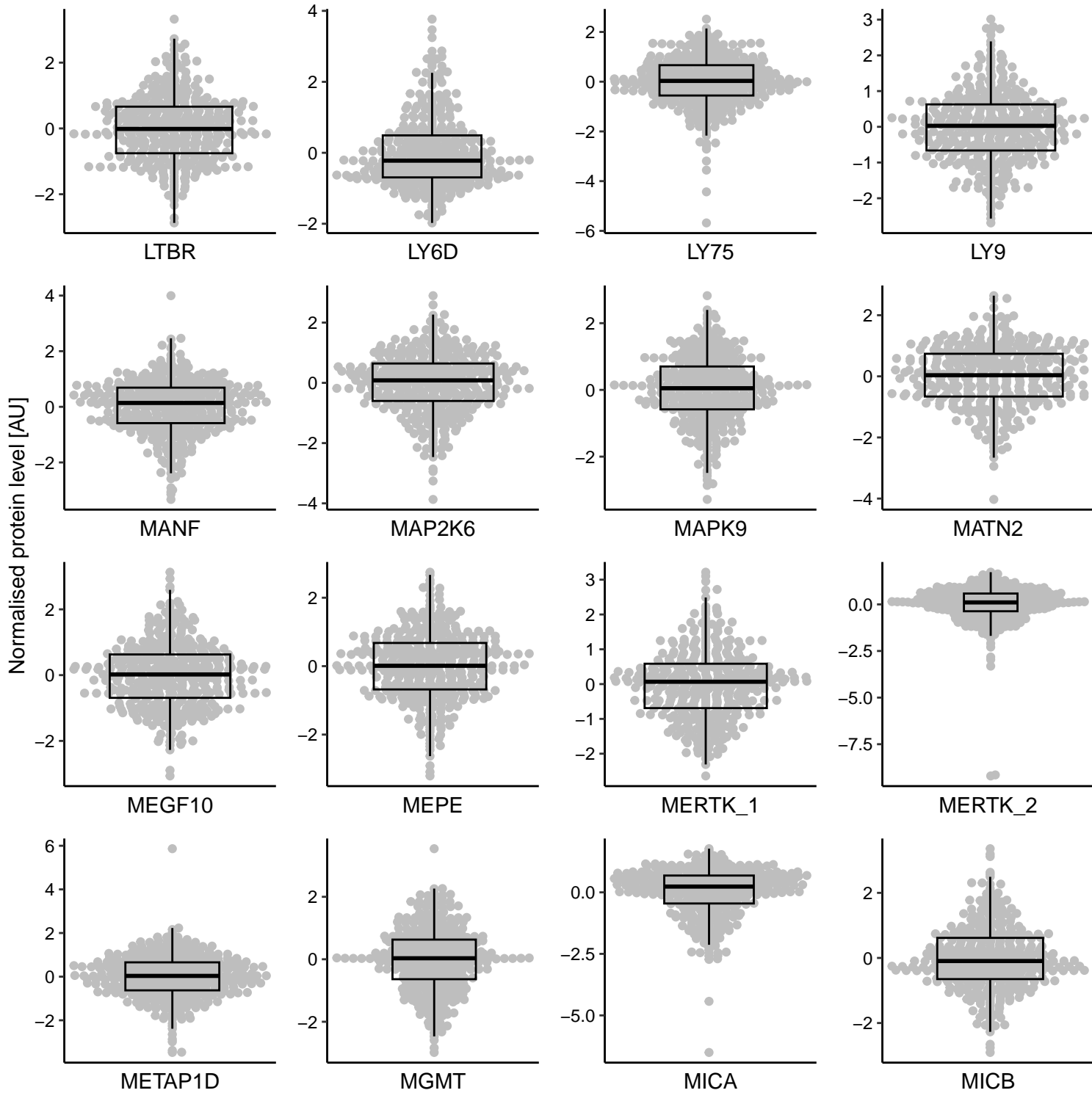

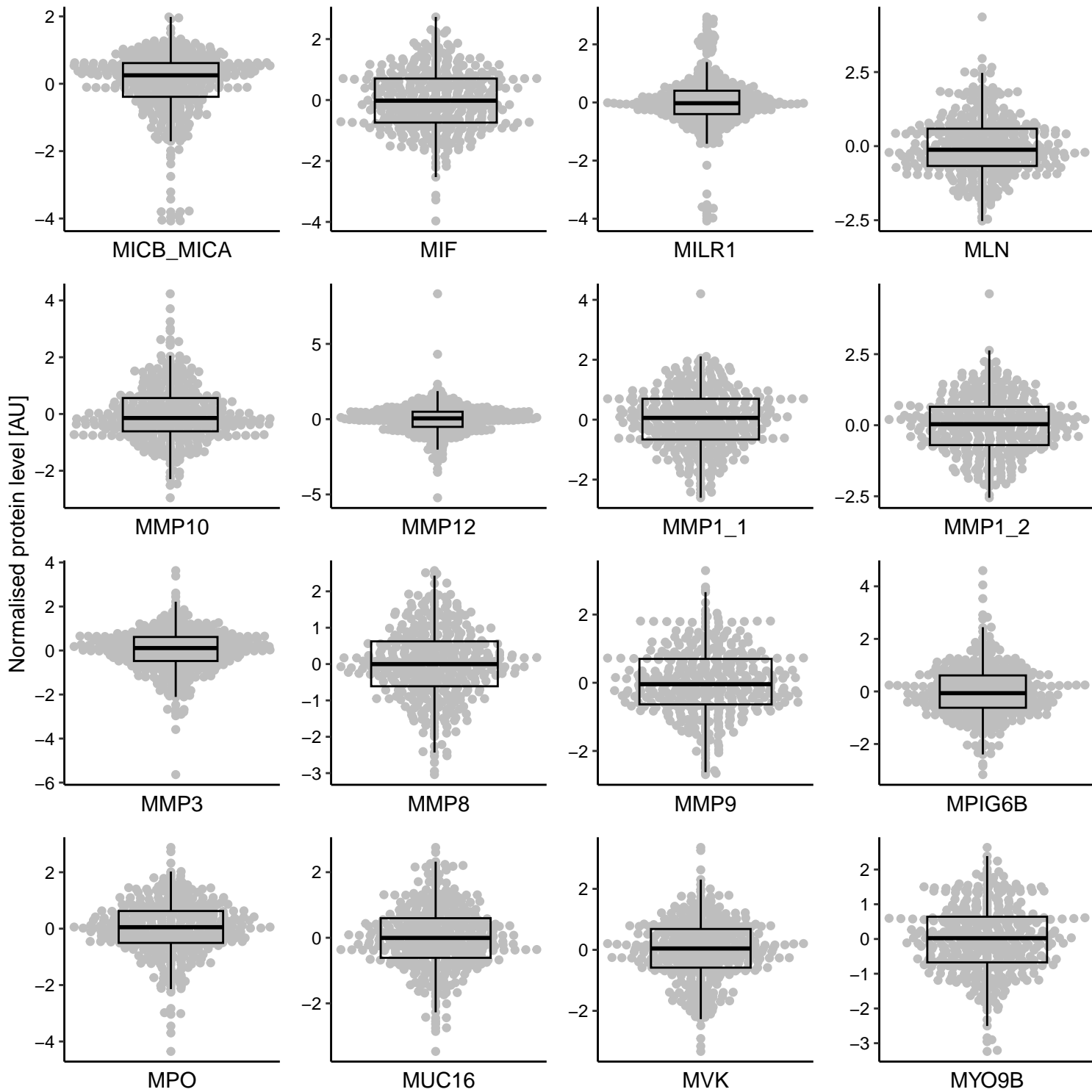

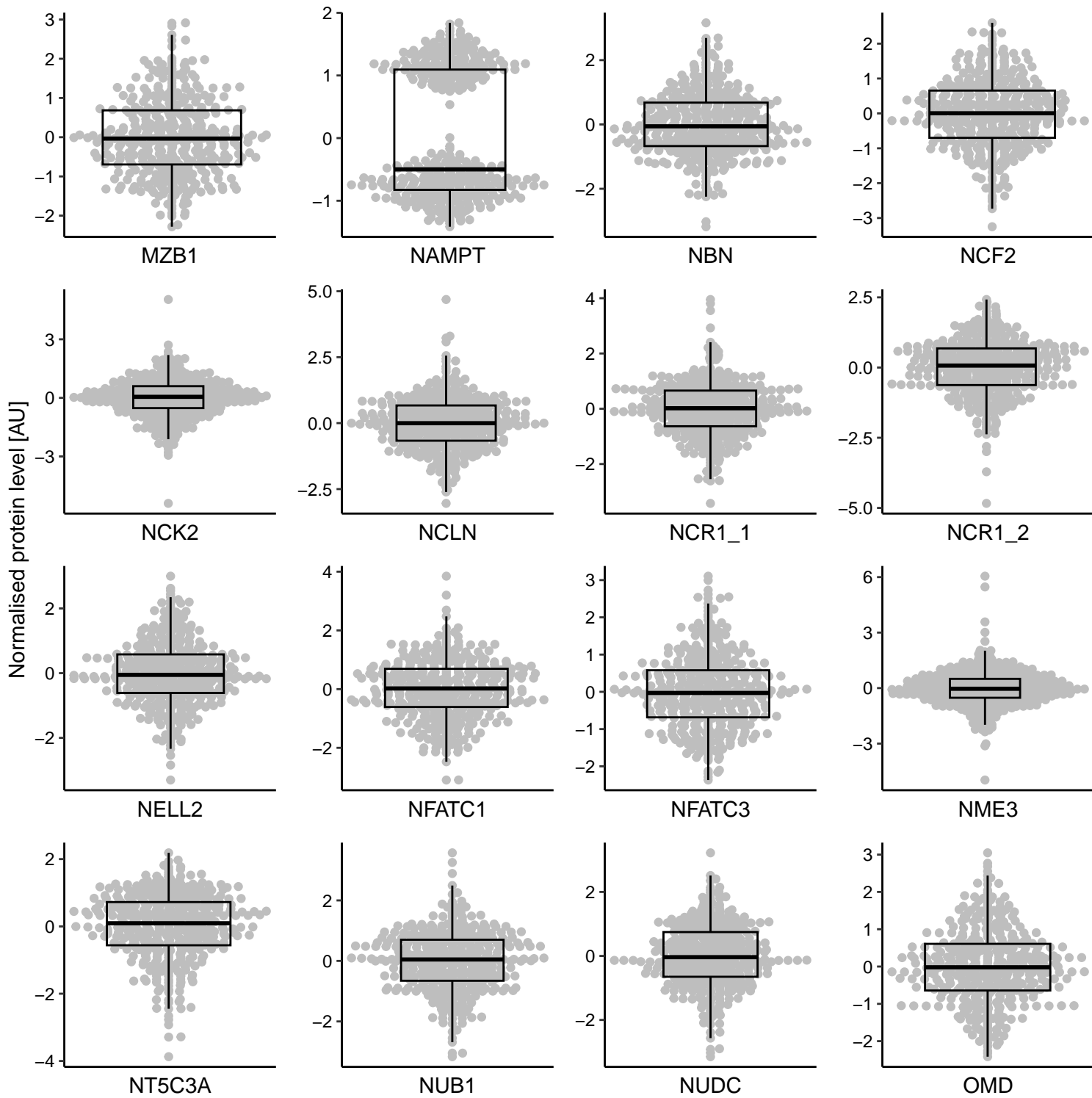

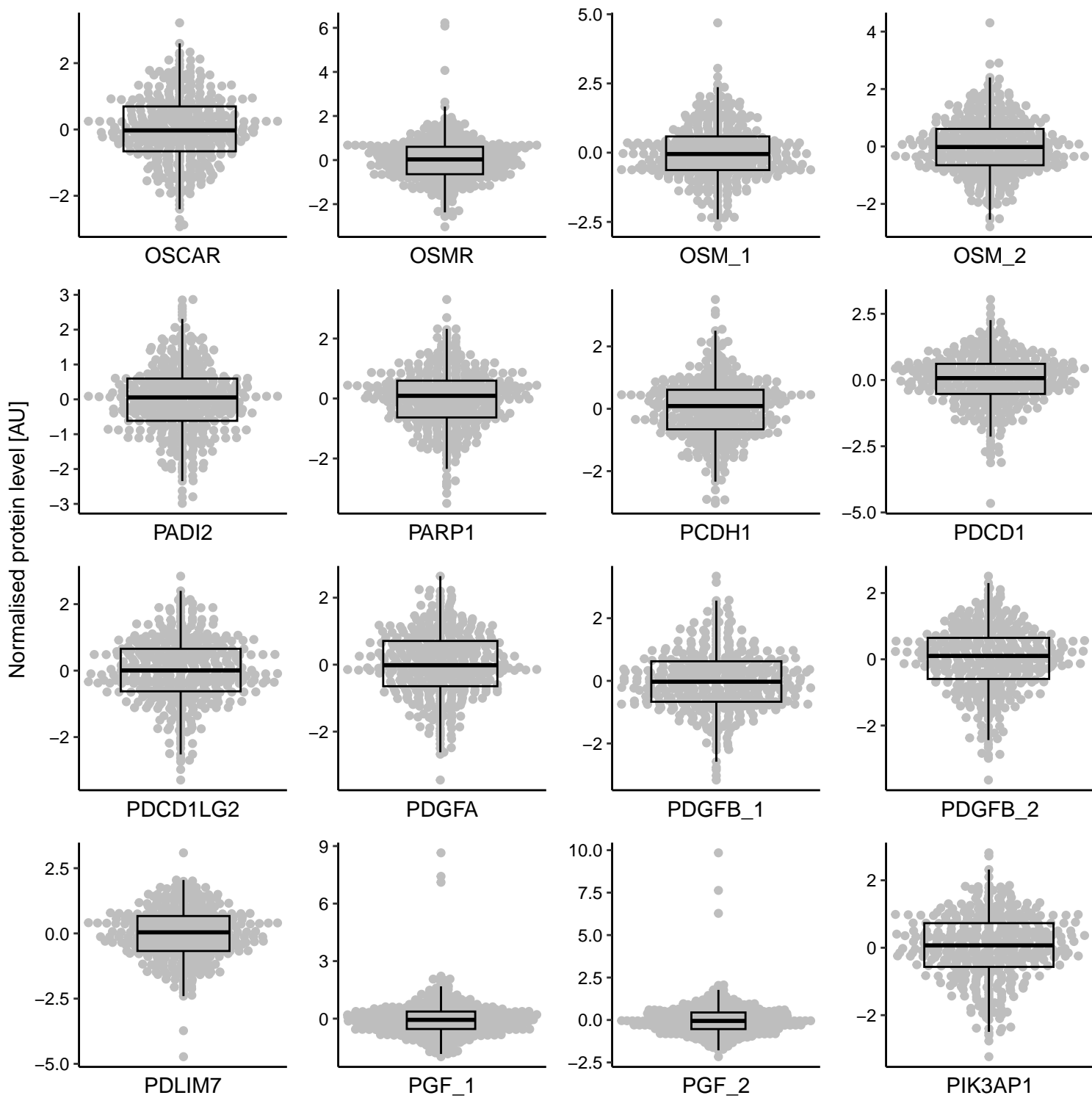

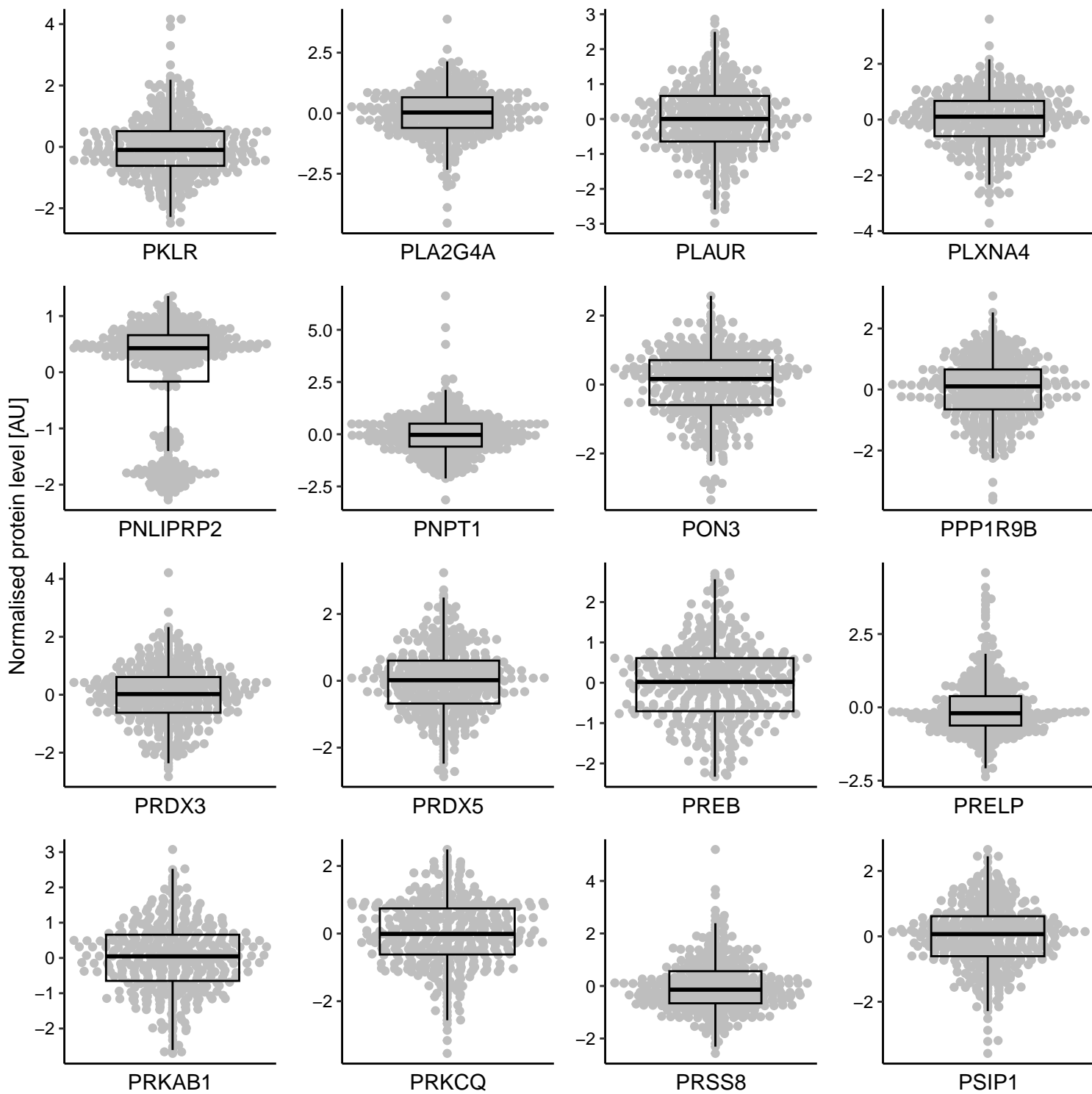

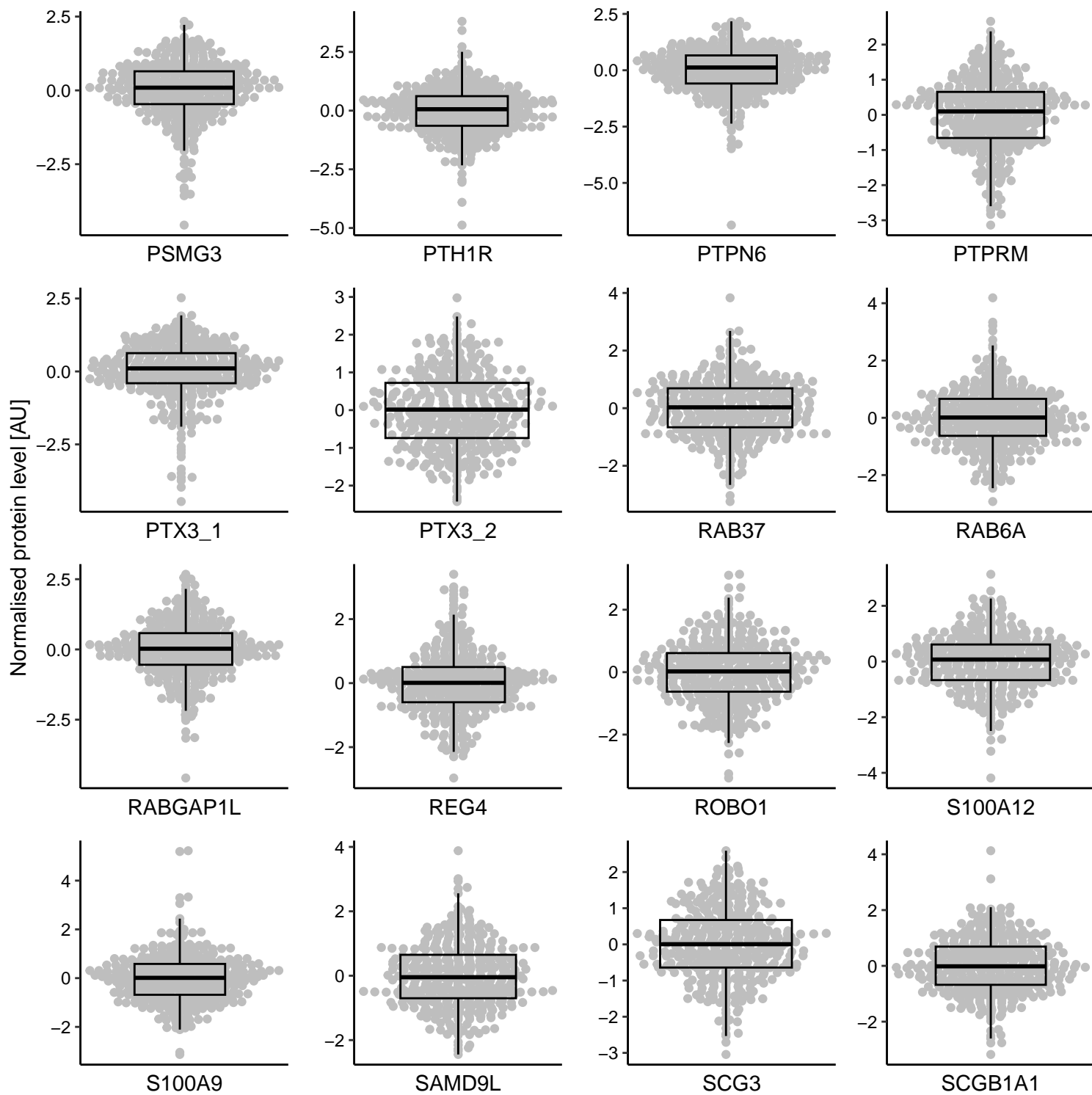

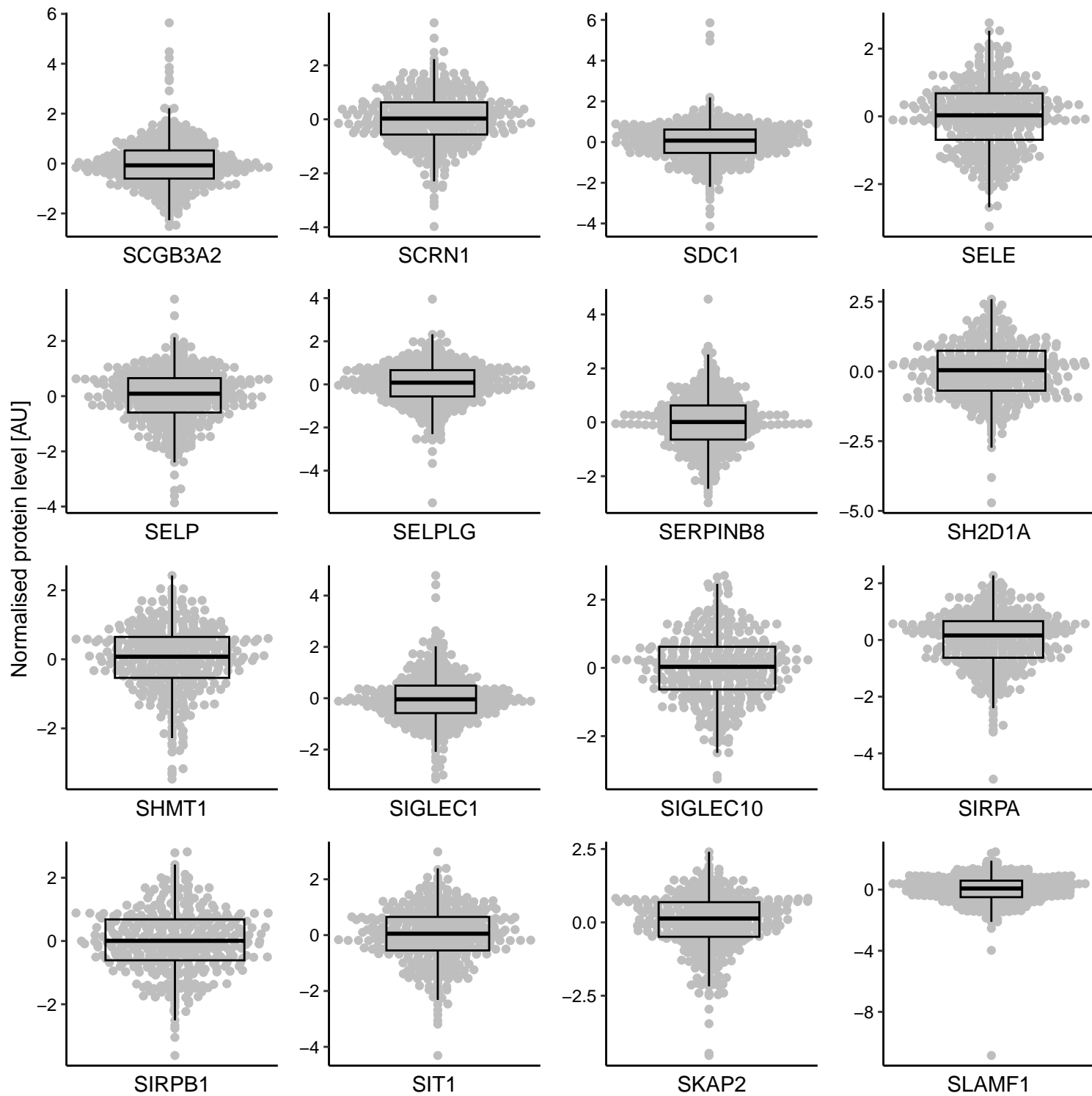

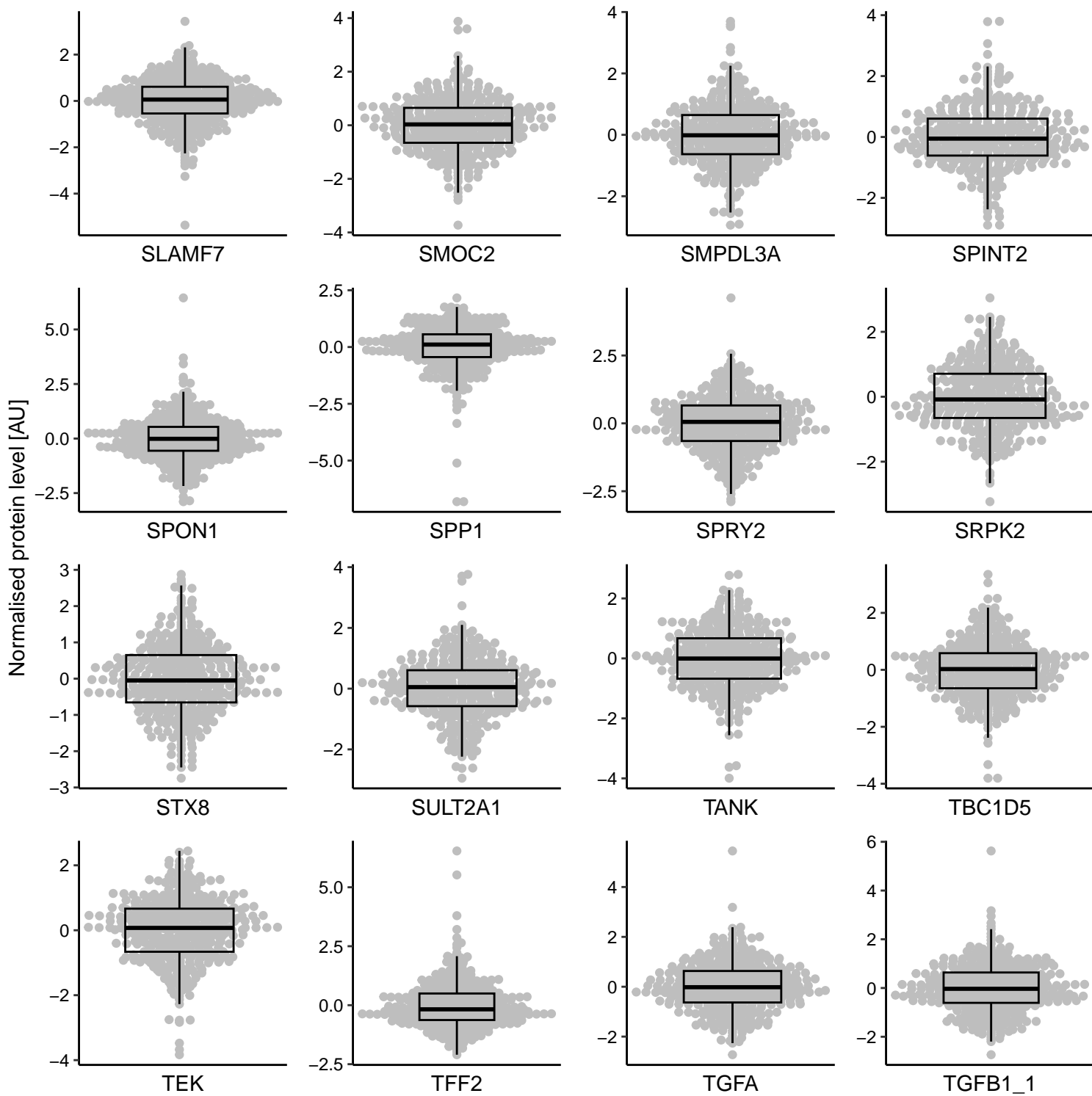

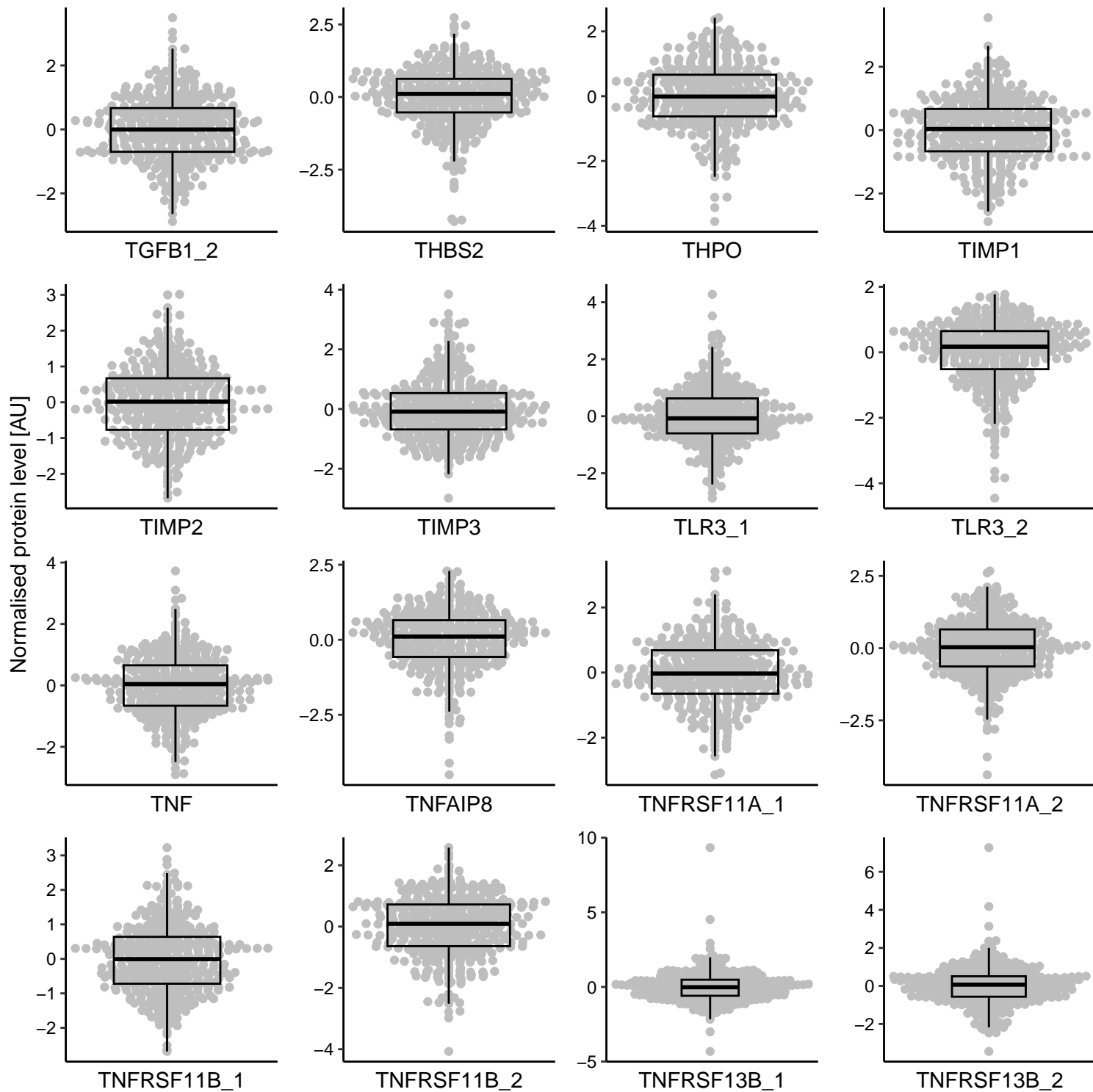

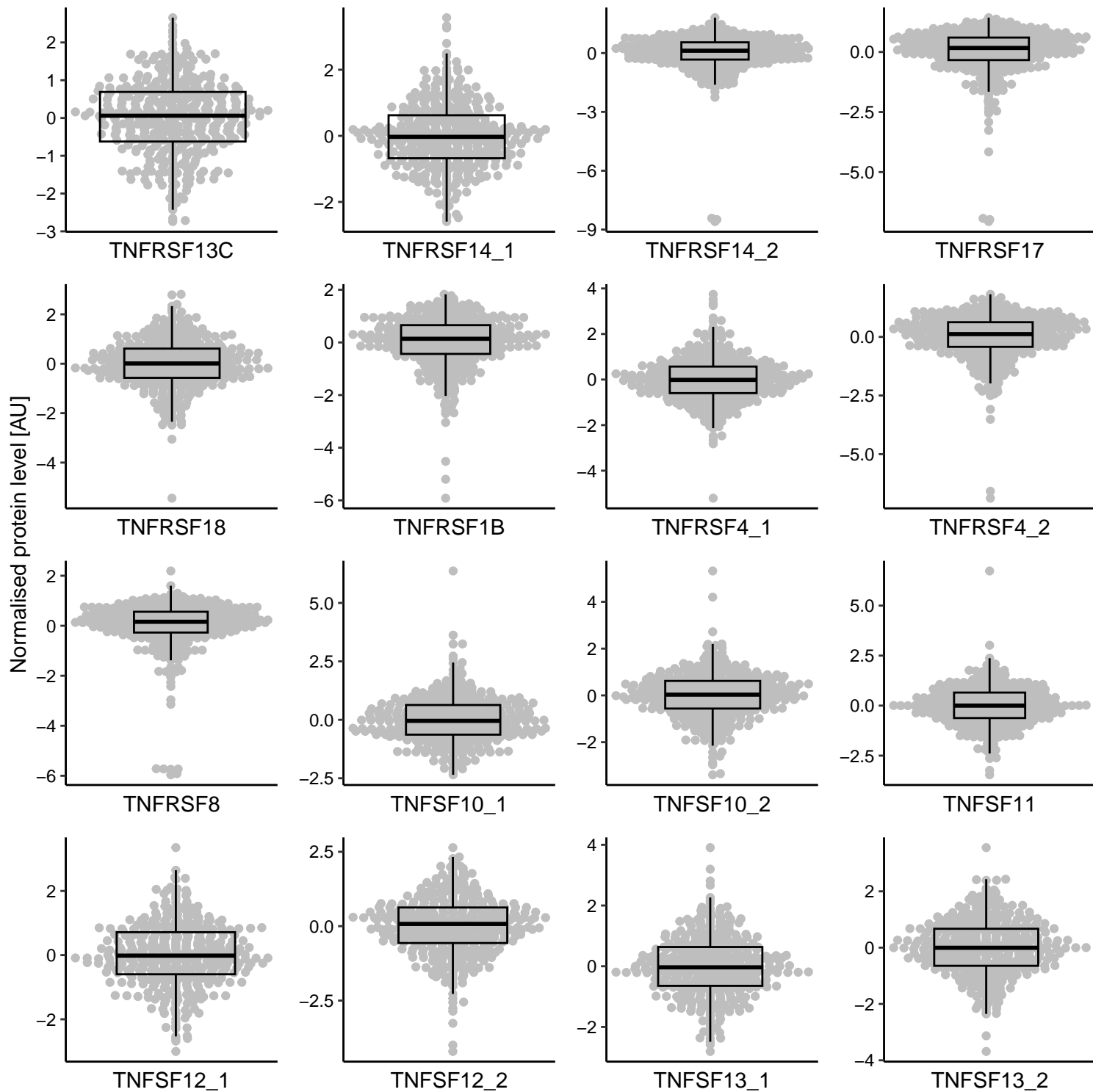

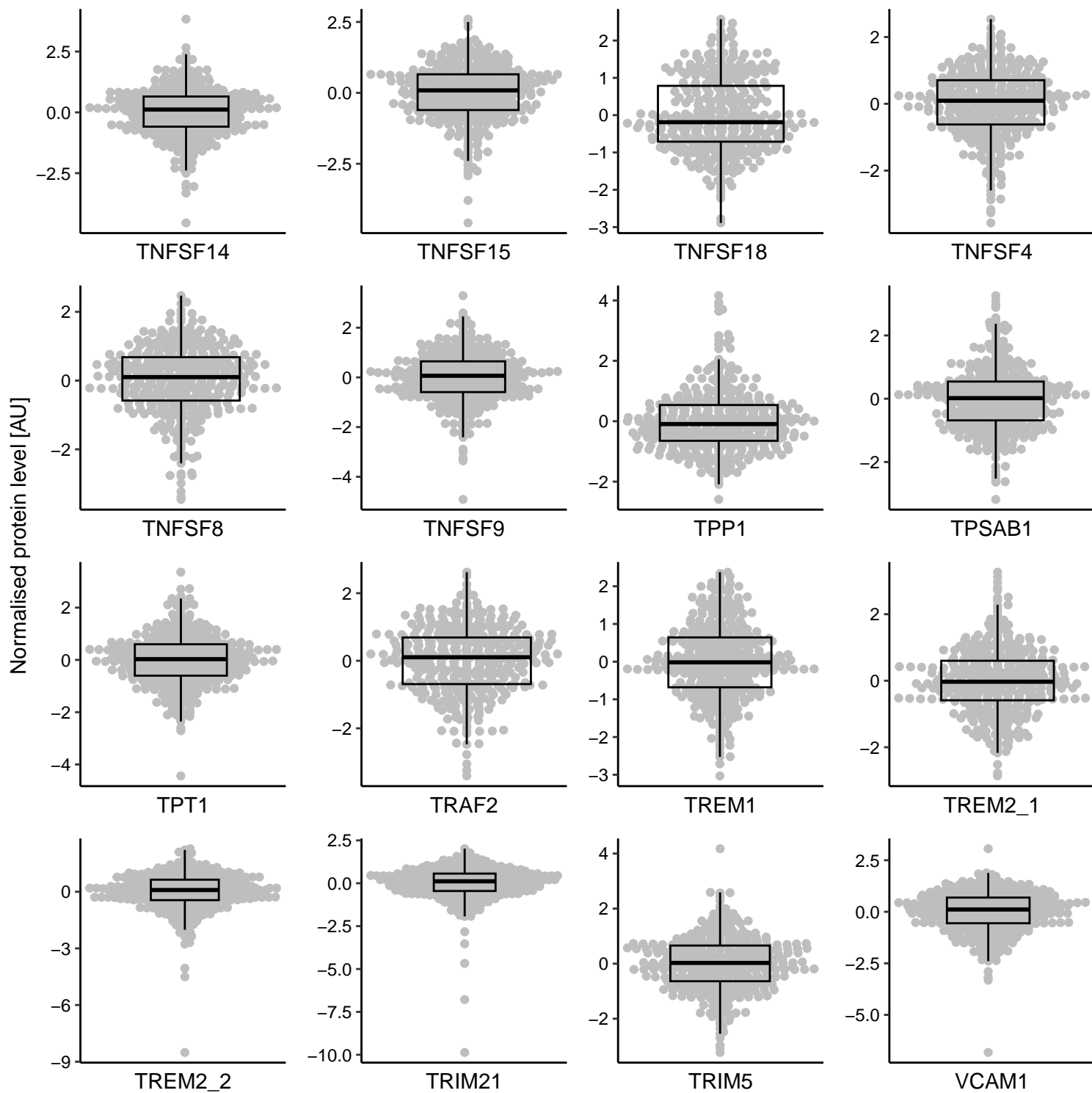

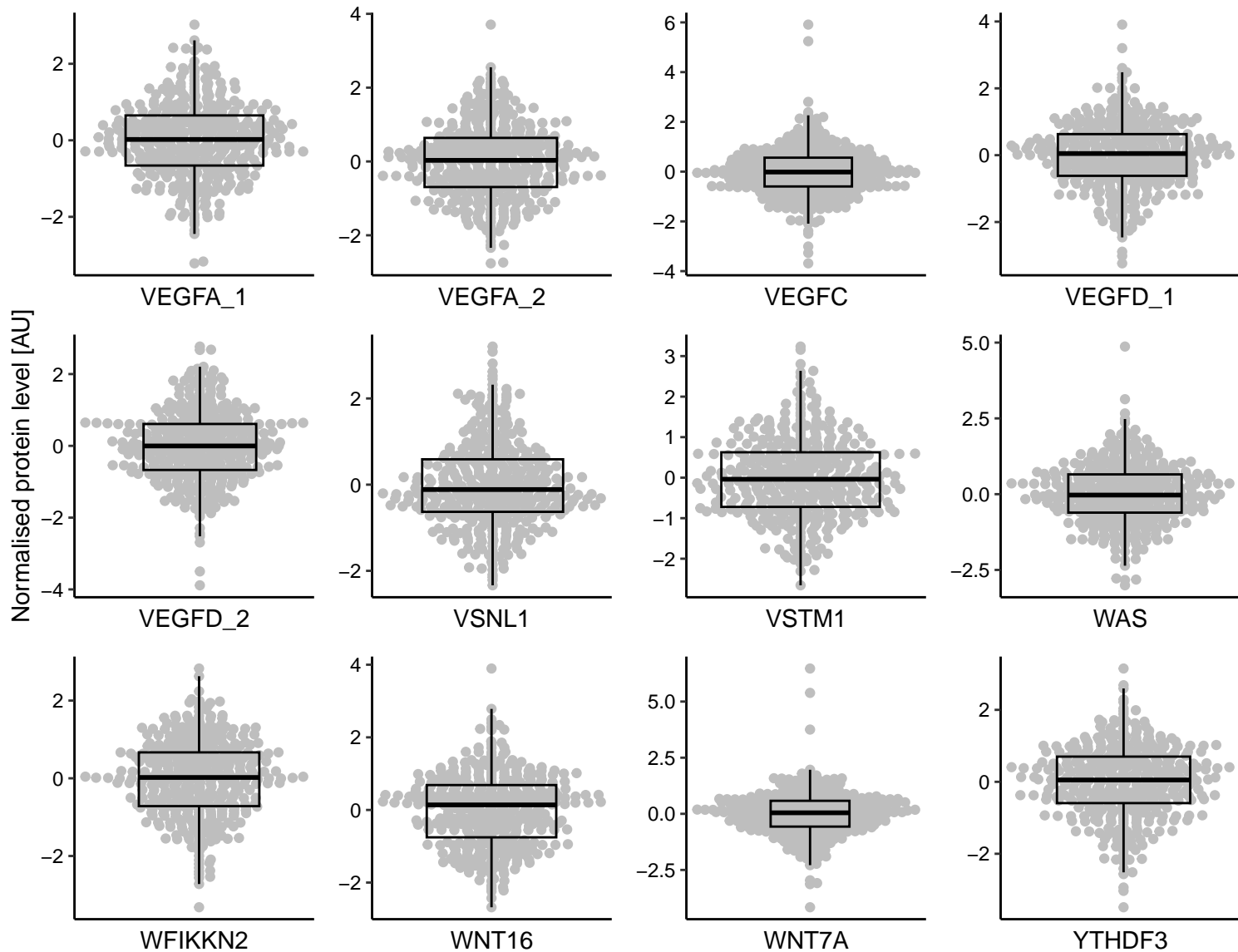

Supplement: Data S3. Boxplots showing the distributions of normalized protein levels from affinity proteomics assays [file mmc4.pdf]
